# Colorectal Cancer Screening Decision Based on Predicted Risk: Protocol for a Pilot Randomized Controlled Trial

**DOI:** 10.2196/46865

**Published:** 2023-09-07

**Authors:** Ekaterina Plys, Jean-Luc Bulliard, Aziz Chaouch, Marie-Anne Durand, Luuk A van Duuren, Karen Brändle, Reto Auer, Florian Froehlich, Iris Lansdorp-Vogelaar, Douglas A Corley, Kevin Selby

**Affiliations:** 1 Center for Primary Care and Public Health (Unisanté), University of Lausanne Lausanne Switzerland; 2 Center for Epidemiology and Research in Population Health, UMR1295 Inserm Université Toulouse III Paul Sabatier Toulouse France; 3 Erasmus MC, University Medical Center Rotterdam Netherlands; 4 Institute of Primary Health Care (BIHAM), University of Bern Bern Switzerland; 5 Department of Gastroenterology, University Hospital of Basel Basel Switzerland; 6 Division of Research, Kaiser Permanente Northern California Oakland, CA United States

**Keywords:** colorectal cancer screening, personalized screening, risk communication, shared decision-making, screening behavior, Switzerland

## Abstract

**Background:**

Incidence of and mortality from colorectal cancer (CRC) can be effectively reduced by screening with the fecal immunochemical test (FIT) or colonoscopy. Individual risk to develop CRC within 15 years varies from <1% to >15% among people aged 50 to 75 years. Communicating personalized CRC risk and appropriate screening recommendations could improve the risk-benefit balance of screening test allocations and optimize the use of limited colonoscopy resources. However, significant uncertainty exists regarding the feasibility and efficacy of risk-based screening.

**Objective:**

We aim to study the effect of communicating individual CRC risk and a risk-based recommendation of the FIT or colonoscopy on participants’ choice of screening test. We will also assess the feasibility of a larger clinical trial designed to evaluate the impact of personalized screening on clinical outcomes.

**Methods:**

We will perform a pilot randomized controlled trial among 880 residents aged 50 to 69 years eligible to participate in the organized screening program of the Vaud canton, Switzerland. Participants will be recruited by mail by the Vaud CRC screening program. Primary and secondary outcomes will be self-assessed through questionnaires. The risk score will be calculated using the open-source QCancer calculator that was validated in the United Kingdom. Participants will be stratified into 3 groups—low (<3%), moderate (3% to <6%), and high (≥6%) risk—according to their 15-year CRC risk and randomized within each risk stratum. The intervention group participants will receive a newly designed brochure with their personalized risk and screening recommendations. The control group will receive the usual brochure of the Vaud CRC screening program. Our primary outcome, measured using a self-administered questionnaire, is appropriate screening uptake 6 months after the intervention. Screening will be defined as appropriate if participants at high risk undertake colonoscopy and participants at low risk undertake the FIT. We will also measure the acceptability of the risk score and screening recommendations and the psychological factors influencing screening behavior. We will also assess the feasibility of a full-scale randomized controlled trial.

**Results:**

We expect that a total sample of 880 individuals will allow us to detect a difference of 10% (α=5%) between groups. The main outcome will be analyzed using a 2-tailed chi-squared test. We expect that appropriate screening uptake will be higher in the intervention group. No difference in overall screening uptake is expected.

**Conclusions:**

We will test the impact of personalized risk information and screening recommendations on participants’ choice of screening test in an organized screening program. This study should advance our understanding of the feasibility of large-scale risk-based CRC screening. Our results may provide insights into the optimization of CRC screening by offering screening options with a better risk-benefit balance and optimizing the use of resources.

**Trial Registration:**

ClinicalTrials.gov NCT05357508; https://www.clinicaltrials.gov/study/NCT05357508

**International Registered Report Identifier (IRRID):**

DERR1-10.2196/46865

## Introduction

### Background

Colorectal cancer (CRC) is the third most common cancer after breast and lung cancers [1]. In 2020, CRC affected approximately 2 million people worldwide and caused 900,000 deaths. In Switzerland, every year, 4500 people are diagnosed with CRC, of whom 1760 die from CRC [2]. However, CRC can be effectively prevented by screening that allows detection and removal of early-stage cancers and precancerous lesions [3-5]. The 2 most frequently offered screening options are colonoscopy and the fecal immunochemical test (FIT). Colonoscopy is more sensitive, especially for polyps [6,7], but it carries risks of bleeding and perforation [8,9]. It also requires an onerous bowel preparation; can be embarrassing for patients; and, as a population-level screening test, requires a large number of gastroenterologists. The FIT is less costly and noninvasive, can be performed at home without preparation, and has higher acceptance [6,10]. The impact of the FIT on CRC mortality over multiple rounds of screening is likely similar to colonoscopy [7,10], and it seems to decrease CRC incidence by approximately 33% [11].

Major risk factors for CRC are advanced adenoma, increasing age, and a small number of rare genetic syndromes [5]. Multiple other factors related to environment and lifestyle influence CRC incidence and can also contribute to one’s risk of developing CRC [12]. One of the best-performing risk calculators is the open-source tool QCancer [12], developed in the United Kingdom and validated on 2 large cohorts of 3.6 million and 2.1 million patients [13,14]. It performed well in comparison with other algorithms [14,15].

Currently, in Switzerland and many other countries, the population is divided into 2 groups: people at average risk of CRC and those at very high risk of CRC because of a personal history of advanced adenomas or CRC, a familial history of CRC, symptoms concerning for cancer, or rare genetic syndromes [5]. Individuals at very high risk are offered regular surveillance colonoscopies and are not part of organized screening programs. However, the population at average risk is not homogeneous and could be broken up into at least 3 groups: high, moderate, and low risk. In 2019, the BMJ Rapid Recommendations team [12] made a weak recommendation against screening for individuals with a CRC risk of <3% in the next 15 years using the QCancer calculator [12]. However, because zero risk does not exist, recommendations against screening are questionable. Nevertheless, the threshold of <3% risk can be used to recommend the FIT and not colonoscopy. In clinical practice, patients with a risk of >6% in the next 15 years are generally offered colonoscopy surveillance. Thus, 6% risk can be considered a threshold for the high-risk level.

These cutoffs could optimize the use of colonoscopy resources, which are limited. The majority of people are at low risk (approximately 60%; J Usher-Smith, PhD, email, June 21, 2020). Offering them colonoscopy results in its overuse and risk of complication for the individuals who are less likely to benefit from screening. On the contrary, reorienting individuals at low risk to the FIT would reduce the number of individuals who choose colonoscopy as a screening test and, therefore, reduce waiting time for those at high risk. Given that the ultimate aim of screening is to diminish mortality, colonoscopies should be reserved for people at high risk because differences in risk reduction between colonoscopy and the FIT become more pronounced for this population [16].

However, the impact of personalized screening on the screening test choice remains unclear. A meta-analysis has shown that personalized risk communication alone does not improve the overall uptake of screening tests [17]. Indeed, communicating personalized risk without giving clear screening recommendations may have no influence on screening behavior or even decrease it. This is in accordance with the view that providing people with threatening information only can decrease desired health behaviors if people cannot cope with the threatening information. However, the studies included in the meta-analysis did not address the choice between the FIT and colonoscopy and contain some important methodological discrepancies.

### Objectives

In summary, personalized screening may offer people better risk-benefit balance in CRC screening and optimize the use of limited colonoscopy resources. However, there is limited knowledge about how the communication of risk and screening recommendations affects CRC screening behaviors. The primary aim of this trial is to study the effect of communicating individual CRC risk score and screening recommendations on appropriate screening uptake at 6 months in individuals stratified into 3 groups: low, moderate, and high risk of developing CRC in the next 15 years. Screening will be considered appropriate if the participant undertakes the test that is adapted to their risk level. Our hypotheses are as follows:

- Compared with the control group, individuals at low risk receiving the intervention will be more likely to undertake the FIT.
- Compared with the control group, individuals at high risk receiving the intervention will be more likely to undertake colonoscopy.
- Individuals at moderate risk receiving the intervention will have the same screening behavior as the individuals in the control group, that is, approximately 50% will be more likely to undertake the FIT, and approximately 50% will be more likely to undertake colonoscopy [18].

We expect no difference in overall screening uptake.

Moreover, we will calculate the participation rate and proportion of individuals eligible for our study. We will also perform subgroup analyses and explore anxiety related to the information about the participants’ risk level.

## Methods

The trial’s protocol follows the SPIRIT (Standard Protocol Items: Recommendations for Interventional Trials) guidelines and the CONSORT (Consolidated Standards of Reporting Trials) statement for randomized controlled trials, as well as the CONSORT-EHEALTH (CONSORT of Electronic and Mobile Health Applications and Online Telehealth) checklist [19,20] (Multimedia Appendix 1). The trial was registered on ClinicalTrials.gov (NCT05357508) on May 3, 2022, before the enrollment of the first participant.

### Study Design and Setting

We will perform a 2-arm randomized superiority controlled trial with participants randomized 1:1 in 2 arms: intervention and usual care. Participants, but not the investigators, will be blinded. The statistician performing the primary outcome analyses will be blinded to group assignments. The trial will be nested in the Vaud CRC screening program.

We used a *patient and public involvement in research* approach throughout by involving 5 members of the target population (residents of the canton of Vaud, Switzerland, aged between 50 and 69 years) in all aspects of the project. This collaboration allowed us to develop materials for our participants that were acceptable, easily understood, and pretested before the trial started. These 5 members will participate in the interpretation and dissemination of the results.

### Participants

**Figure 1:**
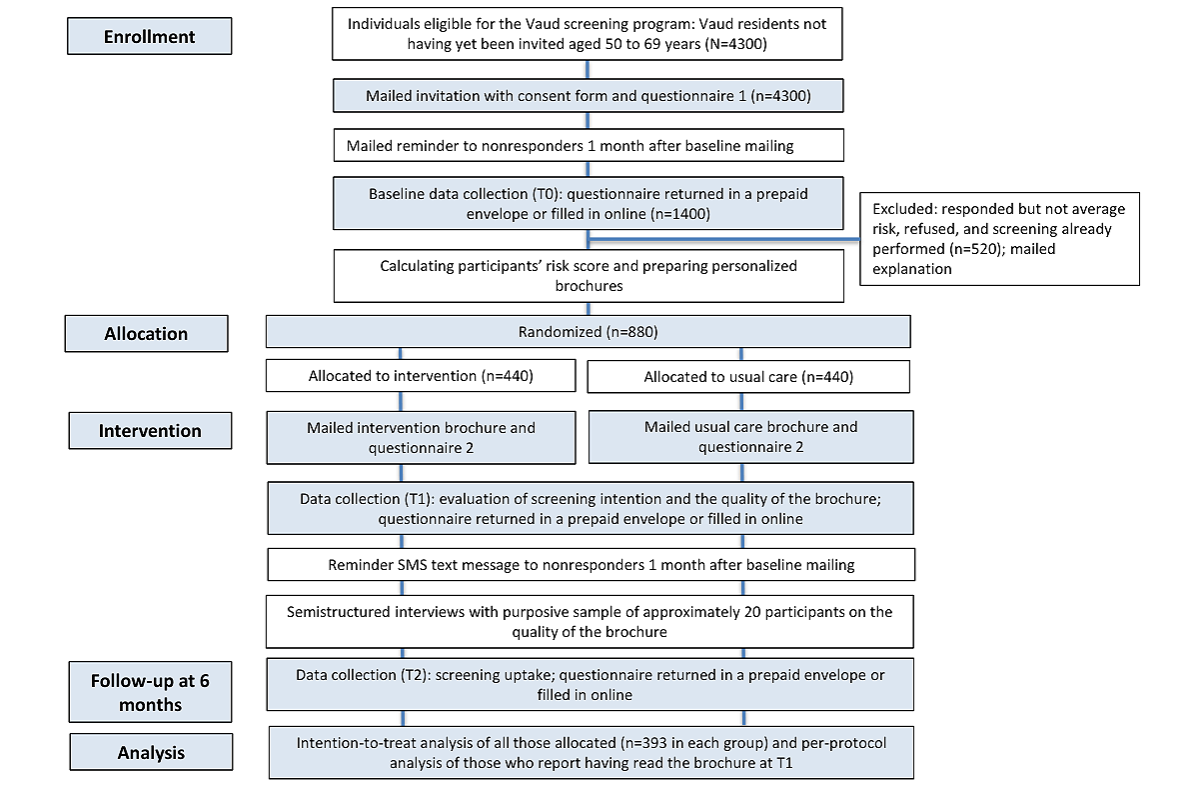
Planned CONSORT (Consolidated Standards of Reporting Trials) diagram for the pilot randomized controlled trial. T1: intervention and secondary outcome data collection; T2: follow-up at 6 months (primary outcome and secondary outcome data collection).

We plan to send out 4300 invitations to recruit 880 eligible participants (Figure 1). Refer to Textbox 1 for the inclusion and exclusion criteria.

Our exclusion criteria allow for the exclusion of individuals at very high risk of CRC (Textbox 1), enabling us to avoid inviting to the study those who are ineligible for the Vaud screening program. Individuals at very high risk are identified using a questionnaire to screen for current symptoms of possible CRC, personal history of CRC or polyp requiring surveillance, inflammatory bowel disease, and genetic syndromes.

Participants can stop their participation at any time without providing any justification. They will be informed that screening uptake is not mandatory and that withdrawal of consent will not affect their subsequent participation in the Vaud screening program.

**Inclusion and exclusion criteria.**

**Inclusion criteria**

- Being a resident of the Vaud canton, Switzerland
- Aged between 50 and 69 years
- Not having yet been invited to the Vaud colorectal cancer (CRC) screening program (this program was gradually rolled out between 2016 and 2022)

**Exclusion criteria**

- Current new symptoms suggestive of CRC
- Personal history of CRC, inflammatory bowel disease, or advanced adenoma or other polyps requiring colonoscopy surveillance at intervals of <10 years
- High-risk familial syndromes for CRC (ie, Lynch syndrome or familial polyposis)
- Having undergone colonoscopy within 9 years or having undertaken the fecal immunochemical test within 1.5 years
- Being unable to participate in follow-up at 6 months

### Procedure

The study will be conducted in the following 3 phases:

- T0: recruitment and baseline data collection
- T1: intervention and secondary outcome data collection
- T2: follow-up at 6 months (primary outcome and secondary outcome data collection)

At T0, an invitation letter, a consent form, and the baseline questionnaire (questionnaire 1) that allows us to verify the eligibility criteria and calculate the CRC risk score will be sent to the potential participants by the Vaud CRC screening program. The ID codes for the participants will be generated using the IDGenerator, a software tool that was specifically developed for epidemiological and clinical trials [21]. The risk score will be calculated for all eligible individuals who return the signed consent form; subsequently, they will be randomized to either the intervention group or the control group. All individuals who do not respond within 1 month after the first mailing will receive a reminder.

At T1, enrolled participants will receive the intervention or a usual care brochure (in accordance with their group allocation), questionnaire 2, and an information sheet that they can provide to their general practitioner and pharmacist regarding their participation in the study. The participants who do not respond to questionnaire 2 within 2 weeks will be sent a reminder. Qualitative interviews will be conducted with willing participants at this time.

Six months after the intervention, at T2, participants will be contacted again to measure their screening behavior using questionnaire 3. Their responses will be accepted up to 8 months after the intervention. In case of nonresponse within 2 weeks, they will receive a reminder.

All 3 questionnaires, information materials for the participants, and the consent form will be available in both paper and electronic formats. Participants can choose the format that is more convenient to them. All data will be entered into the REDCap (Research Electronic Data Capture; Vanderbilt University) platform [22,23].

**Figure 2:**
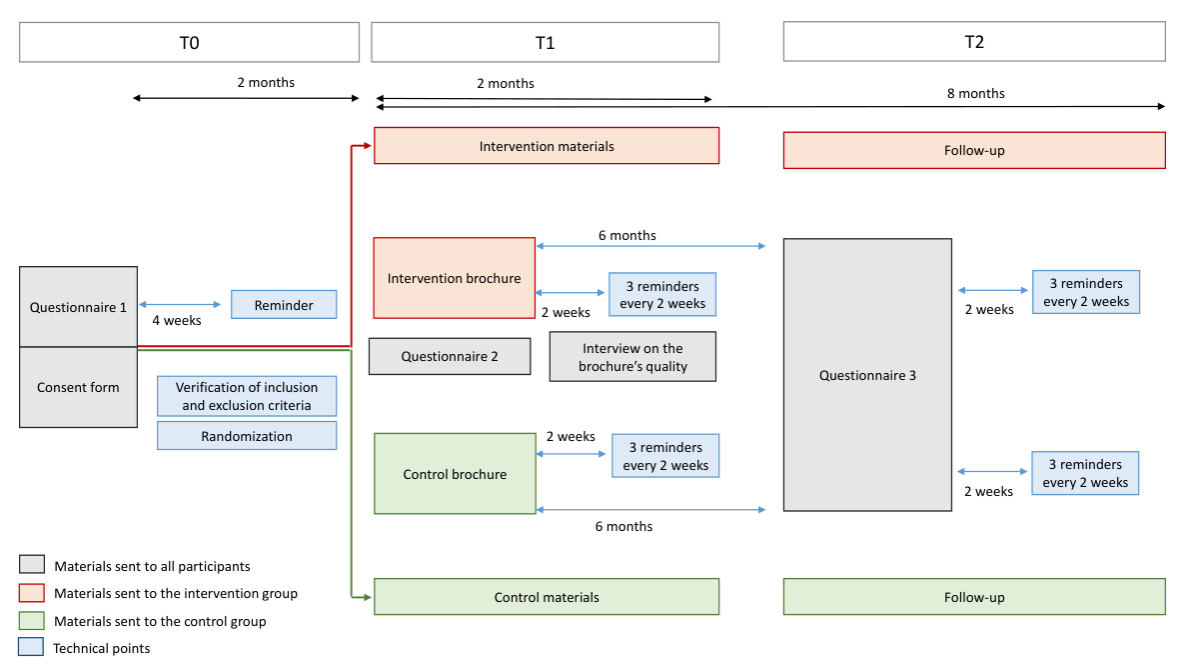
Detailed procedure of the study. T0: recruitment and baseline data collection; T1: intervention and secondary outcome data collection; T2: follow-up at 6 months (primary outcome and secondary outcome data collection).

Refer to Table 1 and Figures 1 and 2 for procedure details.

**Table 1:** Schedule of enrollment, intervention, and assessments.

		Study period			
		Enrollment	Allocation	After the allocation	
		T0^a^	0	T1^b^	T2^c^
**Enrollment**					
	Informed consent	✓			
	Eligibility screening	✓			
	Calculation of the risk for CRC^d^	✓			
	Allocation		✓		
**Intervention**					
	Mailing of the intervention brochure			✓	
	Mailing of the usual care brochure			✓	
**Assessments**					
	Baseline variables: eligibility, risk factors for CRC, previous screening recommendations from a GP^e^, knowledge about screening for CRC, demographics, and contact information	✓			
	Main outcome variables: FIT^f^ uptake, colonoscopy uptake or appointment for colonoscopy				✓
	Secondary outcome variables				
	Intention for screening	✓		✓	✓
	Results of the undertaken screening test				✓
	Self-efficacy for screening tests			✓	
	Emotional reaction to the received risk score			✓	
	Perceived barriers to screening			✓	✓
	Perceived benefits from screening			✓	
	Perceived susceptibility for CRC			✓	
	Quality of the brochure and acceptability of the screening recommendations			✓	
**Qualitative** **interviews**					
	Clarity, completeness, and credibility of the information in the brochure, brochure’s usefulness for decision-making, and suggestions about brochure design			✓	✓
	Motivation for undergoing a specific screening test			✓	✓

^a^T0: recruitment and baseline data collection.

^b^T1: intervention and secondary outcome data collection.

^c^T2: follow-up at 6 months (primary outcome and secondary outcome data collection).

^d^CRC: colorectal cancer.

^e^GP: general practitioner.

^f^FIT: fecal immunochemical test.

### Outcome Measures

The primary outcome measure is the self-reported appropriate screening uptake 6 to 8 months after the intervention (T2), measured using a short self-administered questionnaire. As waiting times can be long for screening colonoscopies [18], participants who have fixed a colonoscopy appointment will be counted as having undergone a colonoscopy. For the secondary outcome measures, refer to Textbox 2, and for the additional measures, refer to Textbox 3.

Refer to Multimedia Appendix 2 for the English version of the questionnaires and Multimedia Appendix 3 for the French version.

**Secondary outcome measures.**

- Self-reported overall screening participation measured at T2 (follow-up at 6 months): on the basis of the participants’ responses at T2, we will calculate the proportion of those who have completed any colorectal cancer (CRC) screening test (fecal immunochemical test or colonoscopy)
- Participation in the study at 6 weeks after the invitation letter was sent out: we will calculate the number of fully completed electronic or paper questionnaires
- Eligibility for the Vaud CRC screening program at 6 weeks after the invitation letter was sent out: we will calculate the proportion of those who are eligible for the Vaud screening program (eg, not up to date with screening, not in colonoscopy surveillance, and no personal CRC history)
- Self-reported anxiety related to the intervention and control materials measured at T1 (intervention) using 6 items inspired by the State-Trait Anxiety Inventory [24]
- Linkage to the Vaud CRC screening program assessed at 3 to 6 months after the primary outcome measurement: we will calculate the proportion of study participants who consent and whose screening status is obtained from the Vaud CRC screening program
- Linkage to the Vaud cancer registry up to 10 years after the primary outcome measurement: we will calculate the proportion of study participants who consent and whose cancer status is obtained from the Vaud cancer registry (a long time frame has been chosen in view of the delay in updating the cancer registry records and interest in cancer outcomes several years after the primary outcome measurement)

**Additional measures.**

**At T0 (baseline)**

- Intention for screening
- Preferences for screening test
- Capacity to participate in screening (no serious health conditions preventing participants from undergoing screening)
- Previous screening recommendations of general practitioner
- Knowledge about screening for colorectal cancer (CRC)
- Demographics

**At T1 (intervention)**

- Perceived susceptibility to CRC
- Perceived benefits of, and barriers to, screening
- Perceived self-efficacy for screening tests
- Participants’ opinions regarding the quality of the brochure
- Acceptance of the provided screening recommendations

**At T2 (follow-up at 6 months)**

- Participants’ barriers to screening (only those who were not screened)

### Theoretical Framework for Understanding the Choice of Screening Test

Several of our outcomes are based on the health belief model [25], which posits that people are more likely to adopt a health-related behavior if they believe that they are vulnerable to the illness (perceived susceptibility) and that the illness is dangerous (perceived severity). Indeed, according to a recent meta-analysis [26], risk appraisals had a small to moderate effect on both the intention to adopt a health-related behavior and the behavior itself. However, the effect sizes for intention and behavior were nearly doubled in size when the interventions aimed at increasing participants’ self-efficacy and perceived efficacy of the recommended behavior [26].

We believe that participants’ screening behavior will largely depend on their perceived susceptibility to CRC as well as their beliefs about their self-efficacy and the efficacy of screening. These elements have been included in our intervention materials.

### CRC Risk Calculation and Risk Levels

The personalized 15-year CRC risk score will be calculated using the QCancer calculator [27]. The QCancer calculator is an open-source algorithm that was developed in the United Kingdom and validated on 2 large cohorts of 3.6 million and 2.1 million patients [28,29]. The QCancer algorithm is based on variables that were available from the patients’ primary care electronic health records. This covers many risk factors for CRC such as age, sex, and personal and family histories of cancers. However, diet and physical activity, which are 2 potentially important CRC risk factors, were not included in the algorithm because the information about them was not routinely collected and included in electronic health records [14]. The QCancer score was chosen because it was developed on a European population, it predicts CRC instead of advanced neoplasia, and it performed well in a large prospective validation study [13]. Despite some limitations, a systematic review showed that the QCancer score allows assigning an individual to the correct risk group with a probability of approximately 70% [14,15]. Although its predictive performance is not perfect, the QCancer risk score gives a good estimation of an individual’s risk level and allows the delivering of personalized recommendations for screening.

For this study, we modified the original algorithm slightly. Our adapted version includes the following factors to calculate the participants’ risk score: age; sex; tobacco and alcohol consumption; family history of CRC; personal history of breast cancer, uterine cancer, cervical cancer, or ovarian cancer for women and personal history of lung cancer, oral cancer, or blood cancer for men; having type 2 diabetes; and BMI values.

Ethnicity, postal code, ulcerative colitis, and colonic polyps will not be used in our adapted algorithm. Ethnicity and postal code have not been validated by studies for use in Switzerland and likely have different effects on CRC risk here than in the United Kingdom. For this reason, the option “White or not stated” will be used by default for all participants, and the field for postal code will be left blank. As our exclusion criteria include advanced adenoma and inflammatory bowel disease, we will indicate that our participants had neither ulcerative colitis nor colonic polyps. We have conducted tests to ensure the robustness of the QCancer score without these elements.

The obtained participant risk scores will be divided into 3 risk levels: low risk (<3%), moderate risk (3% to <6%), and high risk (≥6%).

These cutoffs are related to screening recommendations made to the participants and were based on the evaluation of risk and benefits related to the screening options.

### Intervention and Usual Care Materials

The intervention consists in communicating to the participants their 15-year CRC risk score and recommending the screening test most appropriate for their risk level using specially designed brochures (refer to Multimedia Appendix 4 for the English version of the brochure and Multimedia Appendix 5 for the French version). The intervention brochure is available in the following 3 categories: low CRC risk (<3%), with a recommendation to undertake the FIT; moderate CRC risk (3% to <6%), with a recommendation to either undertake the FIT or colonoscopy; and high CRC risk (≥6%), with a recommendation to undertake colonoscopy.

The intervention brochure provides the 15-year CRC risk score in comparison with that of an *average person* who has a similar profile; for example, an individual who has a CRC risk of 2% (low risk) receives the following message: “According to our estimations, 2 out of 100 people with the same profile as yours will get colon cancer in the next 15 years.”

This message is accompanied by a graphic representation of the risk score and the risk level. Refer to Multimedia Appendix 4 (English version) and Multimedia Appendix 5 (French version) for more information.

Clear screening recommendations presented in the intervention brochure aim to increase participants’ perceived self-efficacy for screening tests. We thus expect that participants at high risk will perceive that they are more susceptible to CRC and, therefore, will be more likely to undertake colonoscopy, which is the most accurate CRC screening test. By contrast, the participants at low risk are expected to perceive their susceptibility to CRC as lower and opt for the noninvasive FIT. To prevent the participants at low risk from declining screening, the brochure informs that zero risk does not exist and strongly recommends undertaking the FIT. No strong recommendations will be made to participants at moderate risk; therefore, the FIT and colonoscopy will be presented in the brochure as equal screening options.

To increase the participants’ perceived response efficacy, our intervention materials emphasize information about the advantages of the recommended test for their risk level. However, the intervention brochure does present an alternative to screening, thus offering a choice to the participants. Moreover, all intervention group participants will receive an information sheet suggesting that they discuss their risk level with their general practitioner.

The intervention brochure will contain recommendations corresponding to the risk level of each participant only. We expect that this will facilitate the understanding of the provided information, minimize reading effort, and enhance adherence to our recommendations. Refer to Table 2 for more information about the content of each brochure category. For the sake of brevity, the intervention brochures will not communicate the side effects or sensitivity of the tests. Both the FIT and colonoscopy are screening tests commonly used in Switzerland and are considered safe and efficient to significantly reduce CRC mortality if undergone within the recommended period (the FIT every 2 years and colonoscopy every 10 years).

The control brochure represents the usual brochure that the Vaud screening program provides to individuals eligible for CRC screening. This brochure recommends screening to all individuals beginning at age 50 years and presents both the FIT and colonoscopy as equal options. This brochure does not include either a personalized risk score or specific screening recommendations. This suggests that, in the control group, the screening tests will be chosen according to the participants’ preferences or after discussion with their primary care physician rather than in accordance with their risk level.

**Table 2:** The different kinds of information presented in the printed materials.

Information presented in the brochure	Intervention brochure				Control brochure
	High risk	Low risk	Moderate risk		
Information about CRC^a^	Yes	Yes	Yes	Yes	
Information about screening benefits	Yes	Yes	Yes	Yes	
Explanation concerning risk levels and how they can be calculated	Yes	Yes	Yes	No	
Personalized risk score	Yes	Yes	Yes	No	
Screening recommendations	Personalized: colonoscopy recommended; the FIT^b^ as an alternative	Personalized: the FIT is recommended; colonoscopy as an alternative	Personalized: the FIT and colonoscopy are equal options	General: the FIT and colonoscopy are equal options	
Explanations why recommended tests are appropriate	Yes	Yes	Yes	No	
Short instructions about how to undertake the FIT and how to prepare bowel for colonoscopy	Yes, instructions for colonoscopy only	Yes, instructions for the FIT only	Yes, instructions for both tests	Yes, instructions for both tests	
Warning that risk increases with age and encouraging statements to maintain a healthy lifestyle	Yes	Yes	Yes	No	
Suggestion to consult a physician if cancer symptoms occur	Yes	Yes	Yes	Yes	
Information about health insurance coverage of the screening tests	Yes	Yes	Yes	Yes	

^a^CRC: colorectal cancer.

^b^FIT: fecal immunochemical test.

### Qualitative Interviews

Semistructured qualitative interviews will be conducted by telephone with a purposive sample of approximately 20 participants using a previously elaborated interview guide until we achieve thematic saturation. The interviews are based on the user-experience “honeycomb” framework described by Morville [30], and they aim to obtain detailed information about the quality of the brochure and the acceptability of the provided screening recommendations. We will be especially interested in the following information: (1) whether the brochure is useful for decision-making about the screening tests; (2) whether participants feel that the brochure improved their knowledge about CRC and screening; (3) whether the information is clear, easy to understand, and credible; and (4) whether the brochure satisfies the participants or whether they have suggestions to improve it.

Purposive sampling will be based on study arm, sex, age, risk level, knowledge of the French language, and reported preferences for the screening test.

### Randomization and Blinding

Randomization will be carried out automatically via the REDCap randomization module programmed by the trial statistician. We will apply a stratified block randomization strategy with a block factor of 8 to prevent a possible imbalance between the control and intervention groups, especially among participants at high risk. The participants will be blinded to the group allocation. They will be told that the study compares 2 slightly different brochures about CRC screening options; however, the nature of the intervention will not be disclosed. The investigators will not be blinded, but, owing to the automatic randomization, they will not be able to influence the participants’ group allocation. The analysis of the main study outcome will be performed by the trial statistician who will be blinded to the participants’ allocation.

### Ethics Approval

The study protocol (project ID 2021-02431) was approved by the ethics committee of the canton of Vaud on March 2, 2022. All the important protocol modifications will be documented and communicated to the ethics committee along with the intermediate and final reports.

### Steering Committee and Trial Monitoring

The trial is supervised by a steering committee composed of a gastroenterologist, a general practitioner, an epidemiologist, and psychologists, as well as experts in shared decision-making and statistical modeling. The members of the steering committee are in charge of approving the trial’s methodology and data analysis plan, and they also contribute in the preparation of oral and written communication materials.

The trial is monitored by an institutional team for trial monitoring that is independent from the trial sponsor and competing interests. The monitoring team will check the quality of collected data as well as conformity with the study protocol. At least 3 site visits are planned, following recommendations from the Swiss Clinical Trial Organization for low-risk trials.

### Statistical Analyses

#### Sample Size Estimation

We expect that 60% of the eligible individuals will be at low risk for CRC, 30% at moderate risk, and 10% at high risk, based on approximate calculations carried out with the UK biobank cohort. The actual distribution of CRC risk factors, and hence 15-year risk scores, in the Vaud population eligible for the screening program is not known.

We also expect that in the intervention group, individuals will be more likely to undertake the appropriate screening test, whereas in the control group, the choice of screening test will not differ among the risk levels (Table 3).

Thus, to detect a difference of 10% (ie, between 46% and 56%) at α=5%, a sample size of 392 individuals in each arm is required to reach 80% power. Assuming approximately 10% attrition, we plan to recruit 440 individuals in each arm (a total of 880 individuals). Our estimation of 10% attrition is based on the results of previous longitudinal studies conducted in the canton of Vaud.

We will invite individuals who have not previously been invited to the Vaud CRC screening program. At the time of sending out the invitation, we will not know whether they are eligible for screening, principally because they may already be up to date with screening or may have had a high-risk polyp needing colonoscopy surveillance. Thus, we plan to send out approximately 4300 invitations; we expect 1400 (32.56%) to respond. Of these 1400 participants, we anticipate that 880 (62.86%) will be eligible for the study (Figure 1). In case of a low response rate, we will send out an additional 2000 invitations.

**Table 3:** Hypothesized size of each group as a proportion of the eligible population and predicted changes in screening choices with personalized recommendations. Group sizes have been chosen to provide sufficient statistical power.

Arm and risk level			Eligible population, %		Choose colonoscopy, %		Choose FIT^a^, %		None, %		Colonoscopy, n/N		FIT, n/N		None, n/N		Overall uptake, %		Appropriately screened, % (n/N)		Overall proportions by randomization
**Control** **(n=392)**																					
	Low	60		34.9		34.9		30.2		82/235		82/235		71/235		70		35 (82/235)		Appropriate screening: 46% (179/392)	
	Moderate	30		34.7		35.6		29.7		41/118		42/118		35/118		70		71 (83/118)		Overall uptake: 70%	
	High	10		35		35		30		14/39		14/39		11/39		70		35 (14/39)		—^b^	
**Intervention** **(n=392)**																					
	Low	60		20.4		49.8		30.2		48/235		117/235		71/235		70		50 (118/235)		Appropriate screening: 56% (220/392)	
	Moderate	30		34.7		35.6		29.7		41/118		42/118		35/118		70		70 (82/118)		Overall uptake: 70%	
	High	10		50		25		25		20/39		10/39		9/39		75		50 (20/39)		—	

^a^FIT: fecal immunochemical test.

^b^Not available.

#### Data Collection

Trial personnel will be trained in good clinical practices and data management. The research team will be provided with the precise guidelines for the inclusion and exclusion criteria, data collection and analysis, and the handling of missing data.

For data collection, we will use 2 validated questionnaires or questionnaires pretested in previous studies. All study materials, including questionnaires, have been reviewed and approved by our citizen advisory group.

All answers to the questionnaire in paper format will be manually entered into the REDCap platform, and 20% of the entries will be checked by another member of the research team. Qualitative interviews will be conducted by a trained team member, recorded, and transcribed. For quality assurance, dual independent coding will be used for 20% of the transcripts, purposively selected. Data triangulation will be used as relevant. Investigator triangulation will also be conducted.

Quantitative and qualitative data will be coded using unique participant identifiers previously generated via the IDGenerator [21]. Paper questionnaires and consent forms will be stored in the investigator site file. Coded data and participant identification lists will be stored separately. Participants’ data will be accessible to authorized personnel only.

Although our trial presents low risk for participants, we will collect information about adverse events; for instance, we will collect information about rectal bleeding or hospitalization after colonoscopy and participants’ anxiety related to the intervention and control materials, as well as the adverse events registered by the Vaud screening program. Serious adverse events will be documented and reported within 24 hours to the sponsor-investigator of the study. Serious adverse events that could be attributable to the trial intervention will be reported to the ethics committee within 15 days.

#### Quantitative Data Analysis Plan

The study data will be summarized across all participants by group and by risk level. Continuous and count variables will be summarized as mean (SD) or median (IQR) and categorical variables as number (percentage). To test randomization quality, differences on the baseline variables will be examined using a 2-tailed *t* test for continuous variables and a 2-tailed chi-squared test for categorical variables.

Self-reported appropriate screening uptake will be analyzed using a 2-tailed chi-squared test and controlled for the CRC risk level. Data analysis will be performed using R statistical software (R Foundation for Statistical Computing) [31] and Stata 16 software (StataCorp LLC) [32].

For the primary outcome, participants lost to follow-up will be considered as having refused to participate in screening. If there seems to be differential loss to follow-up between the intervention and control groups, we may perform multiple imputation based on baseline factors as an exploratory analysis. For other secondary outcomes, data will be considered missing completely at random.

#### Qualitative Data Analysis Plan

For qualitative analysis, we will use the user-experience “honeycomb” framework described by Morville [30]. This approach was used to test health education applications, a decision aid tool [33-36], and the systematic review library of the Cochrane Collaboration [37]. For the purposes of our study, we will use the following 5 (of 7) dimensions:

- Useful: whether the brochure is useful for decision-making about the screening test
- Valuable: whether the brochure improves participants’ knowledge about CRC and screening
- Credible: whether the information in the brochure is perceived as credible
- Usable: whether the information is clear and easy to understand
- Desirable: whether the participants are satisfied by the brochure or whether they have suggestions about how to improve it

The qualitative data will be analyzed using MAXQDA software (VERBI Software GmbH) [38].

## Results

### Overview

As of September 2022, a total of 515 participants had been enrolled in the trial and sent intervention or control materials. Six months later, 98.8% (509/515) of the enrolled individuals were sent the follow-up questionnaire. Data collection was completed on July 24, 2023 (6-8 months after the intervention).

We expect that the proportion of participants who complete the screening test appropriate to their risk level will be higher in the intervention group than in the control group, except for the individuals at moderate risk. The proportion of participants at moderate risk in the intervention group is expected to be the same as that of the participants in the control group in screening test choice because both tests are suggested as equal options.

We do not expect any difference in anxiety between the intervention and control groups. A Cochrane review of interventions similar to ours has shown that there was a nonsignificant trend toward decreased anxiety with risk information (standard mean difference in anxiety 0.13, 95% CI −0.29 to 0.03) [17].

Our intervention does not aim to enhance overall screening uptake; therefore, we do not expect any difference between the groups.

### Dissemination of Results

We plan at least 5 open-access publications based on the trial data. Intermediate and final reports will be sent to the funding institution and to the ethics committee. Participants will be informed about the results of the trial on request. We communicate regularly with the Vaud organized CRC screening program and will encourage it to implement components of this approach if it is found effective.

Suggestions for oral communications and publications as well as names of authors will be discussed during the meetings of the steering committee. The principal investigator should be considered for the role of lead author. Disputes regarding authorship will be settled by the principal investigator and the steering committee.

## Discussion

### Testing the Impact of Risk Communication and Recommendations on Screening Behavior

Although many experts have recommended personalized CRC screening as a means of optimizing the risk-benefit balance for individuals and improving the use of scarce gastroenterology resources, this approach has not been implemented to date in an organized screening program. This study aims to demonstrate the effect of personalized information materials on individuals’ choice of screening test to assess the feasibility of a larger trial evaluating the effect of personalized screening recommendations on clinical outcomes such as advanced neoplasia detection rate and CRC screening uptake. The trial addresses a current need of the Vaud screening program to demonstrate to the public and to physicians that individuals at low risk can be safely directed toward the FIT.

This trial is one of the rare studies that tests the impact of risk communication and recommendations on screening behavior. Although it may seem evident that communicating individuals’ risk level would influence their screening behavior, previous studies have shown increased knowledge but little effect on test choice [17]. This presents a potential limitation in the implementation of personalized screening programs: unless these programs restrict the choice of participants, risk calculation may have no effect.

There are other means of personalizing CRC screening. Several strategies rely simply on participants’ sex or race. In Sweden and Norway, different cutoffs are used to define a positive FIT result requiring colonoscopy [39]. In Germany, colonoscopy screening is recommended at age 50 years for men and age 55 years for women. The American Gastroenterology Society recommends screening at a younger age (45 years) for African Americans. Another approach is to use quantitative FIT results below the positivity threshold but above 0 to define shorter intervals between FITs [40]. We chose to use multiple factors to define baseline risk following recommendations from the BMJ Rapid Recommendations team [12] and because of the strains on colonoscopy capacity in the canton of Vaud [18].

The strengths of this study include its *patient and public involvement in research* approach for the development of study materials; nesting within an organized screening program; population-based sampling to obtain the estimates of participation in the general population; both quantitative and qualitative evaluations, which should permit us to obtain a preliminary estimate of not only efficacy but also acceptability and potential improvements; the use of theory (the Health Belief Model [25]) to inform the intervention and evaluation; and randomization and rigorous design for a pilot study.

Potential weaknesses include the need for informed consent beyond what is typically needed in an organized screening program, which could decrease participation and increase selection bias. As such, we might underestimate eventual participation and overestimate acceptance, assuming that we preferentially include persons motivated to be screened. This selection bias could exclude populations considered more vulnerable, such as individuals with poor health literacy. However, the study materials were written, to the extent legally possible, using plain language. We did not translate the study materials into other languages, but all invitations from the screening program are also in French, with other languages only available on request. The Vaud screening program requested that we not invite individuals already participating in screening at regular intervals with the program because screening recommendations made in the study may differ from those made by the program and thus create confusion. We sought to avoid inviting for this study individuals who had not participated after being sent an invitation to the program, fearing that our participation rate would be too low. As a result, our sample population is heavily weighted toward younger age groups (those aged 50-54 years), and we may have a larger-than-expected proportion of participants at low risk of CRC.

### Conclusions

We are evaluating the effect of communicating individualized CRC risk information and screening recommendations on the choice of screening test in an organized screening program that currently performs the FIT and colonoscopy in a ratio of approximately 50:50. We hypothesize that we can increase FIT use among individuals at low risk and increase colonoscopy use among individuals at high risk, improving the risk-benefit balance of CRC screening. These results will be valuable to screening programs around the world with either a high use of colonoscopy, such as in the canton of Vaud, or FIT programs that may fail to recognize individuals at high risk who could derive additional benefit from colonoscopy over the FIT.

## Appendix

Multimedia Appendix 1 CONSORT-EHEALTH (Consolidated Standards of Reporting Trials of Electronic and Mobile Health Applications and Online Telehealth) checklist.

Multimedia Appendix 2 Questionnaires (English version).

Multimedia Appendix 3 Questionnaires (French version).

Multimedia Appendix 4 Intervention and control brochures (English version).

Multimedia Appendix 5 Intervention and control brochures (French version).

## Abbreviations

CONSORT: Consolidated Standards of Reporting Trials
CONSORT-EHEALTH: Consolidated Standards of Reporting Trials of Electronic and Mobile Health Applications and Online Telehealth
CRC: colorectal cancer
FIT: fecal immunochemical test
REDCap: Research Electronic Data Capture
SPIRIT: Standard Protocol Items: Recommendations for Interventional Trials

## Data Availability

The data sets generated and analyzed during this study are available from the corresponding author on reasonable request.
