# Supplementary material for: Colorectal Cancer Screening Decision Based on Predicted Risk: Protocol for a Pilot Randomized Controlled Trial
Source: JMIR Res Protoc. 2023 Sep 7;12:e46865. doi: 10.2196/46865 (PMC10514773; doi:10.2196/46865)
Supplement: Multimedia Appendix 2 [file resprot_v12i1e46865_app2.pdf]

Questionnaire 1

Your participant code:

Your answers are very important.

Please answer each question without leaving any out.

To complete the online version of the questionnaire

pass through the linkhttps://redcap.link/present

OR

scan the QR code

* * *

The following questions are about any cancer screening tests you may have had in your life.

1.1. Have you ever had a test to look for blood that is invisible to the naked eye in the stool (a FIT test)?

☐ Never

☐ Yes, less than 1.5 years ago

☐ Yes, 1.5 years or more ago

1.2. Have you ever had a colonoscopy? A doctor used a thin, flexible tube to look inside your large intestine through your anus.

☐ Non, never

☐ Yes, less than 9 years ago

☐ Yes, 9 years or more ago

1.3. Has your doctor ever recommended you colon cancer screening?

☐ Oui

☐ Non

1.4. In your opinion, for whom should a colon cancer screening test be recommended?

- ☐ To a healthy person
- ☐ To a person who has symptoms of colon cancer (abdominal pain or blood in the stool)
- ☐ I don't know

1.5. Did you get information about colon cancer screening by the following means *(multiple responses possible):*

- ☐ Your entourage
- ☐ A physician
- ☐ Internet
- ☐ Radio
- ☐ Television
- ☐ Newspapers
- ☐ Drugstore
- ☐ Social networks
- ☐ Other ____

The following questions will allow us to check if colon cancer screening is recommende for you.

1.6. Do you have a known genetic risk for colon cancer (e.g. Lynch syndrome)?

☐ Yes ☐ No ☐ I don't know

1.7. Do you have a chronic inflammatory bowel disease (e.g. Crohn's disease)?

☐ Yes ☐ No ☐ I don't know

1.8. Do you have regular colonoscopy for colonic polyp(s)?

☐ Yes ☐ No

1.9. Have you ever had colon cancer?

☐ Yes ☐ No

1.10. Do you currently have a serious illness that prevents you from participating in screening?

☐ Yes ☐ No ☐ I don't know

1.11. Have you had any of the following symptoms for 3 months or more:

unexplained weight loss	☐ Yes	☐ No
presence of blood in the stool	☐ Yes	☐ No
unusual digestive problems (pain, diarrhea or constipation)	☐ Yes	☐ No

If you answered "Yes" at least once to the questions 1.6-1.11 :

- *You do not need to answer the following questions.*
- *Please return the questionnaire in the envelope provided. Thank you for your valuable participation!*
- *f you are not under the care of a physician for the symptoms or illness you mentioned, we advise you to make an appointment for a check-up.*

* * *

To what extent do you agree with the following statement:

1.12. I intend to undergo a colon cancer screening.

1	2	3	4	5
Completely agree				Totally disagree

1.13. If I ever get tested, I would prefer to

☐ A FIT test that looks for blood that is invisible to the naked eye in the stool.
☐ A colonoscopy, a thin, flexible tube to look inside the large intestine through the anus.
☐ I don't have a preference.
☐ I do not intend to get tested.
☐ Other ____

The following questions will help us estimate your risk of getting colon cancer:

2.1. What is your sex?

☐ Woman ☐ Man

2.2. Do you smoke tobacco?

- ☐ No, I have never smoked
- ☐ No, I quit smoking
- ☐ Yes, less than 10 cigarettes per day
- ☐ Yes, between 10 and 19 cigarettes per day
- ☐ Yes, 20 or more cigarettes per day

2.3. Do you drink alcohol?

- ☐ No, never
- ☐ Yes, once a month at most
- ☐ Yes, 2 to 4 times a month
- ☐ Yes, 2 to 3 times a week
- ☐ Yes, 4 to 6 times a week
- ☐ Yes, every day

If you do not drink alcohol, skip to question 2.5.

2.4. How many standard units do you drink on a typical day when you drink alcohol?

Use the picture with the units of alcohol to answer this question.

- ☐ 1 to 2 units
- ☐ 3 to 6 units
- ☐ 7 to 9 units
- ☐ 10 or more units

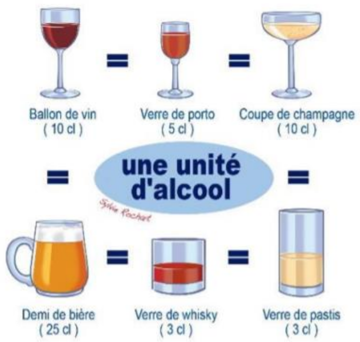

2.5. Has anyone in your immediate family (father, mother, brother, sister, child) had colon cancer?

☐ Yes ☐ No ☐ I don’t know

2.6. Has anyone in your immediate family (father, mother, brother, sister, child) had a polyp in the colon?

☐ Yes	☐ No	☐ I don't know

The following question is <u>for women only</u>. If you are a man, go directly to question 3.2.

3.1. Have you ever had any of the following diseases:

Breast cancer	☐ Yes	☐ No
Uterine cancer	☐ Yes	☐ No
Ovarian cancer	☐ Yes	☐ No
Cervical cancer	☐ Yes	☐ No

3.2. Have you ever had any of the following diseases:

Cancer of the oral cavity (mouth)	☐ Yes	☐ No
Lung cancer	☐ Yes	☐ No
Blood cancer (leukemia, lymphoma or myeloma)	☐ Yes	☐ No

3.3. Has a doctor ever told you that you have diabetes?

- ☐ Yes, I have type 1 diabetes
- ☐ Yes, I have type 2 diabetes
- ☐ No, I do not have diabetes
- ☐ I do not know

In order to carry out our study, we need some additional information.

4.1. What is your year of birth? ____________________

4.2. What is your size (without shoes)? ___________**cm**

4.3. How much do you weigh (without clothes)? _____________**kg**

4.4. What is your current work situation?

- ☐ Full-time employee (32 hours or more per week)
- ☐ Part-time employee (less than 32 hours per week)
- ☐ Homemaker
- ☐ Self-employed
- ☐ Student
- ☐ Unemployed
- ☐ Unable to work
- ☐ Retired
- ☐ I do not wish to answer

4.5. What is your nationality? *(several answers possible):*

- ☐ Switzeland
- ☐ Other : ____

4.6. What is your level of French?

- ☐ Very good
- ☐ Good
- ☐ Not good

4.7. Are you comfortable filling out a medical form on your own (e.g., new doctor's intake questionnaire)?

- ☐ Always
- ☐ Often
- ☐ Sometimes
- ☐ Rarely
- ☐ Never

4.8. What is your couple situation :

- ☐ I live alone
- ☐ I live with a partner
- ☐ I live with my family (children, other family members)
- ☐ Other situation

4.9. What is your highest level of education?

- ☐ Compulsory school or less
- ☐ Apprenticeship
- ☐ High school diploma
- ☐ High school or university
- ☐ I don't know / I don't want to answer

4.10. In the next 6 months, do you plan to leave Switzerland permanently (move permanently to another country)?

- ☐ Yes
- ☐ No
- ☐ Other (please specify) : ____
____

For the next phases of the study, would you like to complete the paper or online questionnaires?

- ☐ I prefer to fill out the paper questionnaires.
- ☐ I prefer to fill out the online questionnaires.

Your contact information

Please provide us with your contact information so that the research team can reach you for the next phases of the study:

☐ Ms. ☐ Mr.

Family name : ____

First name : ____

Telephone number : ____

Address : ____

Postal code: _________ City : ________________________

Email address: ____

We would be grateful if you could return this document using the envelope enclosed with our mail.

If you have any questions, do not hesitate to contact us by e-mail:
etude.present@unisante.ch

We thank you for your answers and your valuable participation!

Questionnaire 2

Your participant code:

The questions below are related to the "Colon Cancer Screening" brochure (you will find the brochure in the same envelope as this questionnaire).

Please read the brochure carefully before answering the questions below. You can of course use the brochure as a guide if necessary.

Your answers are very important. Please answer every question without leaving any out.

To complete this online questionnaire

use the following lind OR scan the QR code

https://redcap.link/present2

* * *

1.1. I confirm that I have read the "Colon Cancer Screening" brochure.

☐ Yes, in full

☐ Yes, in part

☐ No, I haven't read the brochure

Please specify the reason:

☐ The brochure is too long
☐ The brochure is difficult to understand
☐ Other reason: _____________________

After reading the brochure, how much do you agree with the following statements:

2.1. During the next six months, I intend to undergo a FIT test to look for blood that is invisible to the naked eye in the stool.

1 Totally disagree	2	3	4	5 Totally agree

2.2. Within the next six months, I intend to undergo a colonoscopy.

1 Totally disagree	2	3	4	5 Totally agree

2.3. I would be able to do a FIT test for invisible blood at home.

1<br>Totally disagree	2	3	4	5<br>Totally agree

2.4. I would be able to prepare for the colonoscopy, i.e., take a stool softener and eat a 48-hour diet before the exam.

1<br>Totally disagree	2	3	4	5<br>Totally agree

2.5. I can tolerate discomfort related to the colonoscopy, i.e. discomfort and unpleasant sensations that I may experience during the examination.

1<br>Totally disagree	2	3	4	5<br>Totally agree

How did you feel when you read the Colon Cancer Screening brochure?

There is no right or wrong answers. Don't spend too much time on these suggestions and indicate the answer that best describes your feelings.

		Not at all	Somewhat	Moderately	Very much
3.1.	I feel calm	☐	☐	☐	☐
3.2.	I am tense	☐	☐	☐	☐
3.3.	I feel upset	☐	☐	☐	☐
3.4.	I am relaxed	☐	☐	☐	☐
3.5.	I feel content	☐	☐	☐	☐
3.6.	I am worried	☐	☐	☐	☐

If you experience high anxiety, don't hesitate to talk to your doctor.

How much do you agree with the following statements:

		1 Totally desagree	2	3	4	5 Totally agree
4.1.	It is likely that I will get colorectal cancer.	☐	☐	☐	☐	☐
4.2.	My chances of getting colorectal cancer in the next few years are great.	☐	☐	☐	☐	☐
4.3.	I feel I will get colorectal cancer sometime during my life	☐	☐	☐	☐	☐
4.4.	If I find a colorectal cancer through a screening test, my treatment for this illness may not be as bad	☐	☐	☐	☐	☐
4.5.	Having a screening test is the best way for me to find a colorectal cancer early.	☐	☐	☐	☐	☐
4.6.	Having a screening test will decrease my chances of dying from colorectal cancer.	☐	☐	☐	☐	☐
4.7.	Je crois que le dépistage est inutile pour les personnes en bonne santé.	☐	☐	☐	☐	☐
4.8.	I am afraid to have a screening test because I might find out something is wrong.	☐	☐	☐	☐	☐
4.9.	I am disgusted by the test for invisible blood in the stool (FIT).	☐	☐	☐	☐	☐
4.10.	Having a colonoscopy is too embarrassing.	☐	☐	☐	☐	☐

We want to know what you think of the "Colon Cancer Screening" brochure.

To what extent do you agree with the following statements:

5.1. I found the brochure clear and easy to understand.

1<br>Totally agree	2	3	4	5<br>Totally disagree

5.2. I found the brochure useful in making my decision about colon cancer screening.

1<br>Totally agree	2	3	4	5<br>Totally disagree

5.3. I trust the information presented in the brochure.

1<br>Totally agree	2	3	4	5<br>Totally disagree

5.4. If you have any comments or remarks about the brochure, please write them here.

____

____

____

____

In order to learn more about participation in screening, we will be conducting telephone interviews in the near future. This interview will take about 30 minutes and will be held at a time convenient to you.

5.5. Do you agree that the research team should contact you again for a telephone interview?

- ☐ Yes, I would like to participate in the telephone interview.

  Please enter your phone number: ____________________

- ☐ No, I do not wish to participate in this interview.

Additional Questionnaire

Would you like to know your genetic risk?

Another option to find out your risk level for colon cancer is a genetic test. Here are the steps for this test:

Collect some saliva at home using a small jar.

- Mail the jar to a laboratory where the analysis will be done.
- Receive detailed information by mail about your risk of colon cancer with recommendations for screening.
- The test result is stored securely. You choose what to do with the information.

You will only be tested once in your life. The test can also help other members of your family know their risk level for this disease.

We would like to know if you are ever interested in genetic testing.

1. I would like to do the genetic test described above.

1	2	3	4	5
Totally desagree	Disagree	Neither agree nor disagree	Agree	Totally agree

If your answer is 1 or 2, go to question 4 (on the next page).

If your answer is 3, go to question 3.

If your answer is 4 or 5, go to question 2.

2. I would like to be tested for *(several possible answers)*
   - ☐ Know my risk level for colon cancer;
   - ☐ Know which colon cancer screening test is best for me;
   - ☐ Notify other members of my family;
   - ☐ To advance science in this area;
   - ☐ Other *(please specify):* ____________________________________

   ____

3. I'm not sure I want to take this test because *(multiple answers possible)*
   - ☐ I don't want to have this information;
   - ☐ I don't see how this information could be useful to me;
   - ☐ The cost is probably too high;
   - ☐ I am afraid that the results will not be treated confidentially;
   - ☐ Other *(please specify):* ____________________________________

   ____

4. I don't want to be tested because *(multiple answers possible)*

- ☐ I don't want to have this information;
- ☐ I don't see how this information could be useful to me;
- ☐ The cost is probably too high;
- ☐ I am afraid that the results will not be treated confidentially;
- ☐ Other *(please specify):* ____
  ____

We would be grateful if you could return this document in the envelope enclosed with our mail.

If you have any questions, please do not hesitate to contact us by e-mail:
etude.present@unisante.ch

We thank you for your answers and your valuable participation!

Questionnaire 3

Your participant code:

Your answers are very important.
Please answer every question without leaving any out.

To complete this online questionnaire

go through the link OR scan the QR code

https://redcap.unisante.ch/surveys/

* * *

1.1. During the past six months, have you had a colonoscopy or a test to look for blood in the stool that is invisible to the naked eye (a FIT test)?

- ☐ Yes, a colonoscopy
- ☐ Yes, a FIT
- ☐ No, I didn't get tested

1.2. During the past six months, have you made an appointment with a gastroenterologist to have a colonoscopy?

☐ Yes — Go directly to question 1.7.

☐ No — Go directly to question 1.8

1.3. What was your FIT test result

☐ Negative ☐ Positive ☐ I do not know

If you have taken a FIT test, go directly to question 1.6.

1.4. What was the result of your colonoscopy?

- ☐ You do not have a polyp or cancer.
- ☐ You have one or more polyps.
- ☐ You have colon cancer.
- ☐ Other: ______________________________

1.5. Following your colonoscopy, did you experience any complications, including significant bleeding or pain, hospitalization or need for surgery? *(multiple answers are possible)*

- ☐ No
- ☐ Yes, I had significant bleeding or pain following the examination
- ☐ Yes, I had to see a doctor in the office or emergency room
- ☐ Yes, I was hospitalized
- ☐ Yes, I had an operation

1.6. Was your screening test done (will be done) as part of the screening program of the canton of Vaud?

☐	☐	☐
Yes	No	I do not know

1.7. How did you choose the colon cancer screening test *(multiple answers are possible):*

- ☐ I followed the recommendations I received in the "Colon Cancer Screening" brochure.
- ☐ I followed my doctor's advice.
- ☐ I have discussed this with those around me.
- ☐ I used other sources of information (television, internet, newspapers, etc.).
- ☐ Other *(please specify)*: ____

____

You have taken a FIT test.<br>OR<br>You have had a colonoscopy.<br>OR<br>You have made an appointment for a colonoscopy.	You have not had a FIT test.<br><br>You have not had a colonoscopy.<br><br>You have not made an appointment for a colonoscopy.
↓	↓
Go directly to page 4.	Do not answer the questions on pages 3 and 4.

1.8. Over the next six months, I plan to have a FIT test to look for blood in the stool that is invisible to the naked eye.

1<br>Not at all in agreement	2	3	4	5<br>Totally agree

1.9. Within the next six months, I plan to have a colonoscopy.

1<br>Not at all in agreement	2	3	4	5<br>Totally agree

1.10. So far, I have not been tested because *(multiple answers are possible):*

- ☐ I believe screening is recommended for people who have abdominal pain or blood in their stool.
- ☐ I don't see how colon cancer screening can help me.
- ☐ My doctor advised me not to get tested.
- ☐ I see more disadvantages than personal benefits to getting tested.
- ☐ I'm afraid to find out I have cancer.
- ☐ Colonoscopy is too unpleasant for me.
- ☐ I am disgusted by the test for blood in the stool that is invisible to the naked eye (FIT).
- ☐ If I'm going to get colon cancer, screening won't help.
- ☐ I have more important things to worry about than getting tested.
- ☐ I plan to get tested soon.
- ☐ Other. *Précisez, SVP* ____

Additional questionnaire

Would you like to know your genetic risk?

We would like to invite 20-30 participants from the PRESENT study to participate in a second study. This study consists of giving some saliva to do a genetic analysis and calculate the level of risk of colon cancer based on this genetic test. Are you interested in receiving more information about this new study? Receiving more information does not require you to participate in the study.

- ☐ Yes, I would like to have more information about this study

  Thank you for your interest. We will send you more information about the study soon.

  If you prefer to be contacted by email, please provide your email address:

  ____

- ☐ No, I am not interested

We thank you for your answers and your valuable participation!

We would be grateful if you could return this document using the enclosed envelope.

If you have any questions, please do not hesitate to contact us by e-mail:
etude.present@unisante.ch
