# Supplementary material for: Colorectal Cancer Screening Decision Based on Predicted Risk: Protocol for a Pilot Randomized Controlled Trial
Source: JMIR Res Protoc. 2023 Sep 7;12:e46865. doi: 10.2196/46865 (PMC10514773; doi:10.2196/46865)
Supplement: Multimedia Appendix 3 [file resprot_v12i1e46865_app3.pdf]

Questionnaire 1

Votre code de participant.e :

Vos réponses sont très importantes.
Nous vous prions de répondre à chaque question sans en oublier aucune.

Pour remplir ce questionnaire en ligne

passez par le lien	OU	scannez le QR code
https://redcap.link/present		

* * *

Les questions suivantes concernent les tests de dépistage de cancer que vous avez pu faire dans votre vie.

1.1. Avez-vous déjà fait un test pour chercher du sang invisible à l'œil nu dans les selles (un test FIT) ?

- ☐ Jamais
- ☐ Oui, il y a moins de 1 an et demi
- ☐ Oui, il y a 1 an et demi ou plus

1.2. Avez-vous déjà fait une coloscopie ? On utilise un tube fin et souple pour regarder à l'intérieur de votre gros intestin en passant par l'anus.

- ☐ Non, jamais
- ☐ Oui, il y a moins de 9 ans
- ☐ Oui, il y a 9 ans ou plus

1.3. Votre médecin vous a-t-il déjà recommandé de faire le dépistage du cancer du côlon ?

☐ Oui	☐ Non

Avant de passer à la page suivante, veuillez s'il vous plait **vérifier si vous avez répondu à toutes les questions sur cette page. Merci !**

1.4. À votre avis, à qui un test de dépistage du cancer du côlon est-il recommandé ?

- ☐ À une personne en bonne santé
- ☐ À une personne qui a des symptômes de cancer du côlon (douleurs abdominales ou sang dans les selles)
- ☐ Je ne sais pas

1.5. Avez-vous obtenu des informations sur le dépistage du cancer du côlon par les moyens suivants *(plusieurs réponses possibles)* **:**

- ☐ Votre entourage
- ☐ Médecin
- ☐ Internet
- ☐ Radio
- ☐ Télévision
- ☐ Journaux
- ☐ Pharmacie
- ☐ Réseaux sociaux
- ☐ Autre ____

Les questions suivantes servent à savoir si le dépistage du cancer du côlon est conseillé pour vous.

1.6. Avez-vous un risque génétique connu de cancer du côlon (exemple : syndrome de Lynch) ?

☐ Oui ☐ Non ☐ Je ne sais pas

1.7. Avez-vous une maladie inflammatoire chronique du côlon (exemple : maladie de Crohn) ?

☐ Oui ☐ Non ☐ Je ne sais pas

1.8. Avez-vous un contrôle régulier par coloscopie d'un ou plusieurs polype(s) du côlon?

☐ Oui ☐ Non

Avant de passer à la page suivante, veuillez s'il vous plait **vérifier si vous avez répondu à toutes les questions sur cette page. Merci !**

1.9. Avez-vous déjà eu un cancer du côlon ?

☐ Oui ☐ Non

1.10. Souffrez-vous en ce moment d'une maladie grave qui vous empêche de participer au dépistage ?

☐ Oui ☐ Non ☐ Je ne sais pas

1.11. Souffrez-vous depuis 3 mois ou plus d'un des symptômes suivants :

amaigrissement inexpliqué	☐ Oui	☐ Non
présence de sang dans les selles	☐ Oui	☐ Non
troubles digestifs inhabituels (douleurs, diarrhée ou constipation)	☐ Oui	☐ Non

Si vous avez répondu « Oui » au moins une fois aux questions 1.6-1.11 :

- *Vous n'avez pas besoin de répondre aux questions qui suivent.*
- *Veuillez renvoyer le questionnaire à l'aide de l'enveloppe prévue. Merci de votre précieuse participation !*
- *Si vous n'êtes pas suivi par un médecin pour les symptômes ou la maladie que vous avez mentionnés, nous vous conseillons de prendre rendez-vous pour faire un contrôle.*

* * *

A quel degré approuvez-vous l'affirmation suivante :

1.12. J'ai l'intention de faire un dépistage du cancer du côlon.

1	2	3	4	5
Tout à fait d'accord				Pas du tout d'accord

1.13. Si un jour je me fais dépister, je préfèrerais

☐ Un test FIT qui sert à chercher du sang invisible à l'œil nu dans les selles.
☐ Une coloscopie, un tube fin et souple pour regarder à l'intérieur du gros intestin en passant par l'anus.
☐ Je n'ai pas de préférence.
☐ Je n'ai pas l'intention de faire le dépistage.
☐ Autre ______________________________

Avant de passer à la page suivante, veuillez s'il vous plait **vérifier si vous avez répondu à toutes les questions sur cette page. Merci !**

Les questions suivantes nous aideront à estimer votre risque d'avoir un cancer du côlon :

2.1. Quel est votre sexe ?

☐ Femme ☐ Homme

2.2. Fumez-vous du tabac ?

- ☐ Non, je n'ai jamais fumé
- ☐ Non, j'ai arrêté de fumer
- ☐ Oui, moins de 10 cigarettes par jour
- ☐ Oui, entre 10 et 19 cigarettes par jour
- ☐ Oui, 20 cigarettes ou plus par jour

2.3. Buvez-vous de l'alcool ?

- ☐ Non, jamais
- ☐ Oui, une fois par mois au maximum
- ☐ Oui, 2 à 4 fois par mois
- ☐ Oui, 2 à 3 fois par semaine
- ☐ Oui, 4 à 6 fois par semaine
- ☐ Oui, tous les jours

Si vous ne buvez pas d'alcool, passez à la question 2.5.

2.4. Combien d'unités standards buvez-vous au cours d'une journée ordinaire où vous buvez de l'alcool ?

Utilisez l'image avec les unités d'alcool pour répondre à cette question.

- ☐ Moins d'une unité
- ☐ 1 à 2 unités
- ☐ 3 à 6 unités
- ☐ 7 à 9 unités
- ☐ 10 unités ou plus

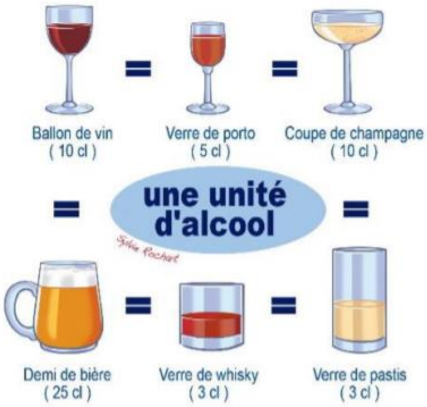

2.5. Une personne de votre famille directe (père, mère, frère, sœur, enfant) a-t-elle eu un cancer du côlon ?

☐ Oui ☐ Non ☐ Je ne sais pas

Avant de passer à la page suivante, veuillez s'il vous plait **vérifier si vous avez répondu à toutes les questions sur cette page. Merci !**

2.6. Une personne de votre famille directe (père, mère, frère, sœur, enfant) a-t-elle eu un polype dans le côlon ?

☐ Oui	☐ Non	☐ Je ne sais pas

La question suivante concerne les femmes uniquement. Si vous êtes un homme, passez directement à la question 3.2.

3.1. Avez-vous déjà eu les maladies suivantes :

Un cancer du sein	☐ Oui	☐ Non
Un cancer de l'utérus	☐ Oui	☐ Non
Un cancer de l'ovaire	☐ Oui	☐ Non
Un cancer du col de l'utérus	☐ Oui	☐ Non

3.2. Avez-vous déjà eu les maladies suivantes :

Un cancer de la cavité buccale (de la bouche)	☐ Oui	☐ Non
Un cancer du poumon	☐ Oui	☐ Non
Un cancer du sang (leucémie, lymphome ou myélome)	☐ Oui	☐ Non

3.3. Un médecin vous a-t-il déjà dit que vous aviez du diabète ?

☐ Oui, j'ai du diabète de type 1
☐ Oui, j'ai du diabète de type 2
☐ Non, je n'ai pas de diabète
☐ Je ne sais pas

Afin de mener à bien notre étude, nous avons besoin de quelques informations supplémentaires.

4.1. Quelle est votre année de naissance ? ____________________

4.2. Quelle est votre taille (sans chaussures) ? ___________cm

4.3. Combien pesez-vous (sans vêtements) ? _____________kg

Avant de passer à la page suivante, veuillez s'il vous plait **vérifier si vous avez répondu à toutes les questions sur cette page. Merci !**

4.4. Quelle est votre situation professionnelle actuelle ?

- ☐ Employé à temps plein (32 heures ou plus par semaine)
- ☐ Employé à temps partiel (moins de 32 heures par semaine)
- ☐ Femme/homme au foyer
- ☐ Indépendant
- ☐ Étudiant
- ☐ Sans emploi
- ☐ Incapacité de travail
- ☐ Retraité
- ☐ Je ne souhaite pas répondre

4.5. Quelle est votre nationalité ? *(plusieurs réponses possibles)*

- ☐ Suisse
- ☐ Autre : ____

4.6. Quel est votre niveau de français ?

- ☐ Très bon
- ☐ Bon
- ☐ Pas bon

4.7. Êtes-vous à l'aise pour remplir vous-même un formulaire médical ? (par exemple : questionnaire d'entrée chez un nouveau médecin) ?

- ☐ Toujours
- ☐ Souvent
- ☐ Parfois
- ☐ Rarement
- ☐ Jamais

4.8. Comment vivez-vous :

- ☐ Je vis seul
- ☐ Je vis avec un.e partenaire
- ☐ Je vis en famille (enfants, autres membres de la famille)
- ☐ Autre situation

4.9. Quel est votre plus haut niveau de formation ?

- ☐ École obligatoire ou moins
- ☐ Apprentissage
- ☐ Maturité gymnasiale
- ☐ Haute école ou université
- ☐ Je ne sais pas / Je ne souhaite pas répondre

Avant de passer à la page suivante, veuillez s'il vous plait **vérifier si vous avez répondu à toutes les questions sur cette page. Merci !**

4.10. Au cours des 6 prochains mois, prévoyez-vous de quitter définitivement la Suisse (déménager définitivement dans un autre pays) ?

- ☐ Oui
- ☐ Non
- ☐ Autre (veuillez préciser) : ____
  ____

Pour les prochaines phases de l'étude, souhaitez-vous remplir les questionnaires papiers ou en ligne ?

- ☐ Je préfère remplir les questionnaires papiers.
- ☐ Je préfère remplir les questionnaires en ligne.

Vos coordonnées de contact

Merci de nous indiquer vos coordonnées pour que l'équipe de recherche puisse vous joindre pour les prochaines phases de l'étude :

☐ Madame ☐ Monsieur

Nom : ____

Prénom : ____

Numéro de téléphone : ____

Adresse : ____

NPA : __________ Localité : ___________________________

Adresse mail : ____

Nous vous serions reconnaissants de retourner ce document à l'aide de l'enveloppe jointe à notre courrier.

Si vous avez des questions, n'hésitez pas à nous contacter par e-mail :
etude.present@unisante.ch

Nous vous remercions pour vos réponses et votre précieuse participation !

Questionnaire 2

Votre code de participant.e : 25470

Les questions présentées ci-dessous sont en lien avec la brochure « Le dépistage du cancer du côlon » (vous trouvez la brochure dans la même enveloppe que ce questionnaire).

Nous vous prions de lire attentivement la brochure avant de répondre aux questions ci-dessous. Vous pouvez bien sûr vous aider de la brochure, si nécessaire.

Vos réponses sont très importantes. Nous vous prions de répondre à chaque question sans en oublier aucune.

Pour remplir ce questionnaire en ligne

passez par le lien	OU	scannez le QR code
https://redcap.link/present2		

* * *

1.1. Je confirme avoir bien lu la brochure « Dépistage du cancer du côlon ».

☐ Oui, en entier

☐ Oui, en partie

☐ Non, je n'ai pas lu la brochure

Merci de préciser la raison :

- ☐ La brochure est trop longue
- ☐ La brochure est difficile à comprendre
- ☐ Autre raison : ___________________

Après avoir lu la brochure, à quel point approuvez-vous les affirmations suivantes :

2.1. Durant les six prochains mois, j'ai l'intention de faire un test FIT pour chercher du sang invisible à l'œil nu dans les selles.

1<br>Pas du tout d'accord	2	3	4	5<br>Tout à fait d'accord

2.2. Durant les six prochains mois, j'ai l'intention de faire une coloscopie.

1<br>Pas du tout d'accord	2	3	4	5<br>Tout à fait d'accord

2.3. J'arriverais à faire un test FIT pour la recherche du sang invisible à l'œil nu chez moi.

1<br>Pas du tout d'accord	2	3	4	5<br>Tout à fait d'accord

2.4. J'arriverais à me préparer à la coloscopie, c'est-à-dire prendre une préparation qui facilite l'évacuation des selles et faire 48 heures de régime alimentaire avant l'examen.

1<br>Pas du tout d'accord	2	3	4	5<br>Tout à fait d'accord

2.5. Je peux supporter l'inconfort lié à la coloscopie, c'est-à-dire des gênes et des sensations désagréables que je pourrais ressentir pendant l'examen.

1<br>Pas du tout d'accord	2	3	4	5<br>Tout à fait d'accord

Qu'avez-vous ressenti quand vous avez lu la brochure sur le dépistage du cancer du côlon ?

Il n'y a pas de bonne ni de mauvaise réponse. Ne passez pas trop de temps sur ces propositions et indiquez la réponse qui décrit le mieux vos sentiments.

		Non	Plutôt non	Plutôt oui	Oui
3.1.	Je me suis senti.e tranquille, bien dans ma peau.	☐	☐	☐	☐
3.2.	Je me suis senti.e tendu.e, crispé.e.	☐	☐	☐	☐
3.3.	Je me suis senti.e ému.e, bouleversé.e, contrarié.e.	☐	☐	☐	☐
3.4.	Je me suis senti.e décontracté.e, détendu.e.	☐	☐	☐	☐
3.5.	Je me suis senti.e satisfait.e.	☐	☐	☐	☐
3.6.	Je me suis senti.e inquiet.e, soucieux.se.	☐	☐	☐	☐

Si vous ressentez une anxiété élevée, n'hésitez pas à en parler avec votre médecin traitant.

A quel point approuvez-vous les affirmations suivantes :

	1<br>Pas du tout d’accord	2	3	4	5<br>Tout à fait d’accord
4.1. Il est probable que j’aurai un cancer du côlon.	☐	☐	☐	☐	☐
4.2. Mes chances d'avoir un cancer du côlon dans les prochaines années sont grandes.	☐	☐	☐	☐	☐
4.3. Je pense que j'aurai un cancer du côlon un jour ou l'autre dans ma vie.	☐	☐	☐	☐	☐
4.4. Si on détecte un cancer du côlon grâce à un test de dépistage, mon traitement pour cette maladie sera peut-être moins lourd.	☐	☐	☐	☐	☐
4.5. Passer un test de dépistage est le meilleur moyen de détecter un cancer du côlon à un stade précoce.	☐	☐	☐	☐	☐
4.6. Le fait de passer un test de dépistage diminuera mes risques de mourir d'un cancer du côlon.	☐	☐	☐	☐	☐
4.7. Je crois que le dépistage est inutile pour les personnes en bonne santé.	☐	☐	☐	☐	☐
4.8. J'ai peur de passer un test de dépistage car je pourrais découvrir que quelque chose ne va pas.	☐	☐	☐	☐	☐
4.9. Le test de recherche de sang invisible à l’œil nu dans les selles (FIT) me dégoûte.	☐	☐	☐	☐	☐
4.10. Faire une coloscopie est trop embarrassant.	☐	☐	☐	☐	☐

Nous souhaitons savoir ce que vous pensez de la brochure « Dépistage du cancer du côlon ».

À quel degré approuvez-vous les affirmations suivantes :

5.1. J'ai trouvé la brochure claire et facile à comprendre.

1<br>Pas du tout d'accord	2	3	4	5<br>Tout à fait d'accord

5.2. J'ai trouvé la brochure utile pour prendre ma décision par rapport au dépistage du cancer du côlon.

1<br>Pas du tout d'accord	2	3	4	5<br>Tout à fait d'accord

5.3. Je fais confiance aux informations présentées dans la brochure.

1<br>Pas du tout d'accord	2	3	4	5<br>Tout à fait d'accord

5.4. Si vous avez des commentaires ou des remarques sur la brochure, n'hésitez pas les écrire ici.

____

____

____

____

Afin d'en savoir plus sur la participation au dépistage, nous organiserons des entretiens téléphoniques prochainement. Cet entretien prendra environ 30 minutes et aura lieu à un moment qui vous conviendra.

5.5. Êtes-vous d'accord pour que l'équipe de recherche vous recontacte pour un entretien téléphonique ?

- ☐ Oui, je souhaite participer à l'entretien téléphonique.
  Indiquez votre numéro de téléphone, s'il vous plaît : ________________________
- ☐ Non, je ne souhaite pas participer à cet entretien.

Questionnaire supplémentaire

Souhaiteriez-vous connaitre votre risque génétique ?

Une autre option pour connaitre votre niveau de risque du cancer du côlon est un test génétique. Voici les étapes de ce test :

- Recueillir un peu de salive à la maison en utilisant un petit pot.
- Envoyer le pot par la poste à un laboratoire où l'analyse sera faite.
- Recevoir une information détaillée par la poste sur votre risque de cancer du côlon avec des recommandations pour le dépistage.
- Le résultat du test est stocké en toute sécurité. C'est vous qui choisissez que faire avec l'information.

Vous ne ferez l'analyse qu'une seule fois dans votre vie. Le test peut également aider d'autres membres de votre famille à connaitre leur niveau de risque pour cette maladie.

Nous aimerions savoir si vous souhaitez un jour faire un test génétique.

1. Je voudrais faire le test génétique décrit ci-dessus.

1	2	3	4	5
Pas du tout d'accord	Pas d'accord	Ni d'accord, ni pas d'accord	D'accord	Tout à fait d'accord

Si votre réponse est 1 ou 2, allez à la **question 4** (sur la page suivante).

Si votre réponse est 3, allez à la **question 3.**

Si votre réponse est 4 ou 5, allez à la **question 2**.

2. Je voudrais me faire tester pour *(plusieurs réponses possibles)*
   - ☐ Connaitre mon niveau de risque du cancer du côlon ;
   - ☐ Savoir quel examen de dépistage du cancer du côlon est le mieux adapté pour moi ;
   - ☐ Aviser d'autres membres de ma famille ;
   - ☐ Faire avancer la science dans ce domaine ;
   - ☐ Autre *(précisez, s'il vous plaît) :* ______________________________

3. Je ne suis pas certain.e de vouloir faire ce test car *(plusieurs réponses possibles)*
   - ☐ Je n'ai pas envie d'avoir cette information ;
   - ☐ Je ne vois pas comment cette information pourrait m'être utile ;
   - ☐ Le coût est sûrement trop élevé ;
   - ☐ J'ai peur que les résultats ne soient pas traités de manière confidentielle ;
   - ☐ Autre *(précisez, s'il vous plaît) :* ______________________________

4. Je ne veux pas me faire tester car *(plusieurs réponses possibles)*
- ☐ Je n'ai pas envie d'avoir cette information ;
- ☐ Je ne vois pas comment cette information pourrait m'être utile ;
- ☐ Le coût est sûrement trop élevé ;
- ☐ J'ai peur que les résultats ne soient pas traités de manière confidentielle ;
- ☐ Autre *(précisez, s'il vous plaît) :* ____
  ____

Nous vous serions reconnaissants de retourner ce document à l'aide de l'enveloppe jointe à notre courrier.

Si vous avez des questions, n'hésitez pas à nous contacter par e-mail :
etude.present@unisante.ch

Nous vous remercions pour vos réponses et votre précieuse participation !

Questionnaire 3

Votre code de participant.e : «ID»

Vos réponses sont très importantes. Nous vous prions de répondre à chaque question sans en oublier aucune.

Pour remplir ce questionnaire en ligne

passez par le lien https://redcap.unisante.ch/surveys/ et entrez le code suivant : HANPTMPPY

OU

scannez le QR code

* * *

1.1. Durant les six derniers mois, avez-vous fait une coloscopie ou un test pour chercher du sang invisible dans les selles à l'œil nu (un test FIT) ?

- ☐ Oui, une coloscopie
- ☐ Oui, un FIT
- ☐ Non, je n'ai pas fait le dépistage

1.2. Durant les six derniers mois, avez-vous pris un rendez-vous avec un médecin gastroentérologue pour faire une coloscopie ?

☐ Oui — Passez directement à la question 1.7.

☐ Non — Passez directement à la question 1.8

1.3. Quel était le résultat de votre test FIT

☐ Négatif ☐ Positif ☐ Je ne sais pas

Si vous avez fait un test FIT, passez directement à la question 1.6.

1.4. Quel était le résultat de votre coloscopie ?

- ☐ Vous n'avez ni polype ni cancer.
- ☐ Vous avez un ou plusieurs polypes.
- ☐ Vous avez un cancer du côlon.
- ☐ Autre : ____

1.5. Suite à votre coloscopie, avez-vous eu des complications, notamment un saignement ou des douleurs importantes, une hospitalisation ou un besoin d'être opéré.e ? *(plusieurs réponses sont possibles)*

- ☐ Non
- ☐ Oui, j'ai eu un saignement ou des douleurs importantes suite à l'examen
- ☐ Oui, j'ai dû consulter un.e médecin en cabinet ou aux urgences
- ☐ Oui, j'ai été hospitalisé.e
- ☐ Oui, j'ai été opéré.e

1.6. Est-ce que votre test de dépistage a été fait (sera fait) dans le cadre du programme de dépistage du canton de Vaud ?

☐	☐	☐
Oui	Non	Je ne sais pas

1.7. Comment avez-vous choisi le test de dépistage du cancer du côlon *(plusieurs réponses sont possibles) :*

- ☐ J'ai suivi les recommandations reçues dans la brochure « Le dépistage du cancer du côlon ».
- ☐ J'ai suivi le conseil de mon médecin.
- ☐ J'en ai discuté avec mon entourage.
- ☐ J'ai utilisé d'autres sources d'information (télévision, internet, journaux, etc.).
- ☐ Autre *(précisez, s'il vous plaît)* : ____

____

Vous avez fait un test FIT.
OU
Vous avez fait une coloscopie.
OU
Vous avez pris un rendez-vous pour une coloscopie.

→ Passez directement à la page 4.

Vous n'avez pas fait un test FIT.

Vous n'avez pas fait une coloscopie.

Vous n'avez pas pris un rendez-vous pour une coloscopie.

→ Répondez pas aux questions sur les pages 3 et 4.

1.8. Durant les six prochains mois, j’ai l'intention de faire un test FIT pour chercher du sang invisible à l'œil nu dans les selles.

1<br>Pas du tout d’accord	2	3	4	5<br>Tout à fait d’accord

1.9. Durant les six prochains mois, j’ai l'intention de faire une coloscopie.

1<br>Pas du tout d’accord	2	3	4	5<br>Tout à fait d’accord

1.10. Jusqu’à présent, je n’ai pas fait le test de dépistage parce que *(plusieurs réponses sont possibles) :*

- ☐ Je crois que le dépistage est recommandé aux personnes qui ont des douleurs abdominales ou du sang dans les selles.
- ☐ Je ne vois pas en quoi le dépistage du cancer du côlon peut être utile pour moi.
- ☐ Mon médecin m’a déconseillé de faire le dépistage.
- ☐ Je vois plus d’inconvénients que de bénéfices personnels à me faire dépister.
- ☐ J’ai peur d’apprendre que j’ai un cancer.
- ☐ La coloscopie m’est trop désagréable.
- ☐ Le test de recherche de sang invisible à l’œil nu dans les selles (FIT) me dégoûte.
- ☐ Si je dois avoir un cancer du côlon, le dépistage ne sera d’aucune utilité.
- ☐ J’ai des soucis plus importants que de faire un dépistage.
- ☐ J’ai l’intention de faire le dépistage prochainement.
- ☐ Autre. *Précisez, SVP* ____

Questionnaire supplémentaire

Souhaiteriez-vous connaitre votre risque génétique ?

Nous souhaitons inviter 20 à 30 participants de l'étude PRESENT à participer à une deuxième étude. Cette étude consiste à donner un peu de salive pour faire une analyse génétique et calculer le niveau de risque de cancer du côlon sur la base de ce test génétique. Est-ce que vous êtes intéressé.e à recevoir plus d'informations sur cette nouvelle étude ? Recevoir plus d'information ne vous oblige pas à participer à l'étude.

☐ Oui, je souhaite avoir plus d'information sur cette étude

Merci pour votre intérêt. Nous vous transmettons prochainement des informations sur l'étude.

Si vous préférez être contacté.e par un message électronique, veuillez indiquer votre adresse e-mail, s'il vous plait :

____

☐ Non, je ne suis pas intéressé.e

Nous vous remercions pour vos réponses et votre précieuse participation !

Nous vous serions reconnaissants de retourner ce document à l'aide de l'enveloppe jointe.

Si vous avez des questions, n'hésitez pas à nous contacter par e-mail :
etude.present@unisante.ch
