# Supplementary material for: Colorectal Cancer Screening Decision Based on Predicted Risk: Protocol for a Pilot Randomized Controlled Trial
Source: JMIR Res Protoc. 2023 Sep 7;12:e46865. doi: 10.2196/46865 (PMC10514773; doi:10.2196/46865)
Supplement: Multimedia Appendix 4 [file resprot_v12i1e46865_app4.pdf]

How to do a FIT test at home?

1 Go to a pharmacy:
A pharmacist explains the test to you.
The pharmacist gives you the test and its instructions.

Or

Make an appointment with your family doctor:
Your doctor gives you a prescription. You then pick up the test and its instructions at a pharmacy.

2 Take my FIT test at home
— First, you collect the stool on a special paper that you stick to the toilet seat.
— You then scrape the stool several times with the stem of the collection tube.
— You send the tube by mail to the laboratory.

3 You and your doctor will receive the test results by mail within a week.

The test is negative

The test showed nothing. This means that there is no sign of cancer in the stool. The test must be done again in 2 years.

▶ If you have any symptoms in the meantime, see your doctor right away.

The test is positive

The test shows blood in the stool. This does not necessarily mean that you have a cancer. But the cause of the blood in the stool must be sought.

▶ Make an appointment with your family doctor to arrange a colonoscopy.

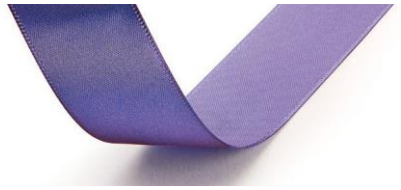

When should you see a doctor?

There is no test that detects all cancers at an early stage.
If you have any symptoms listed below, make an appointment with your family doctor:

- Blood in the stool, digestive disorders,
- Belly pain, diarrhea or constipation, more or less frequent stools than usual, unexplained weight loss,
- Long-lasting fatigue.

How is screening managed?

Both tests are covered by health insurance companies for people between 50 and 69 years old living in the canton of Vaud.
You will not pay a deductible. You will still have to pay the 10% deductible, i.e. about CHF 5 for the FIT test.
If you are over 69, ask your doctor if screening is still right for you.

For more information
Vaud cancer screening programs: www.pvdc.ch Swiss Cancer Screening : www.swisscancerscreening.ch Your family doctor or pharmacist.

v3.0_avril_2022

Unisanté
Route de Berne 113, 1010 Lausanne
Tel: 0848 990 990, Fax: 021 314 14 46
depistage.colon@unisante.ch, wwwpvdc.ch

unisanté
Centre universitaire de médecine générale et santé publique • Lausanne

Dépistage du cancer du côlon
Canton de Vaud

COLON CANCER SCREENING

From 50 years old I am informed

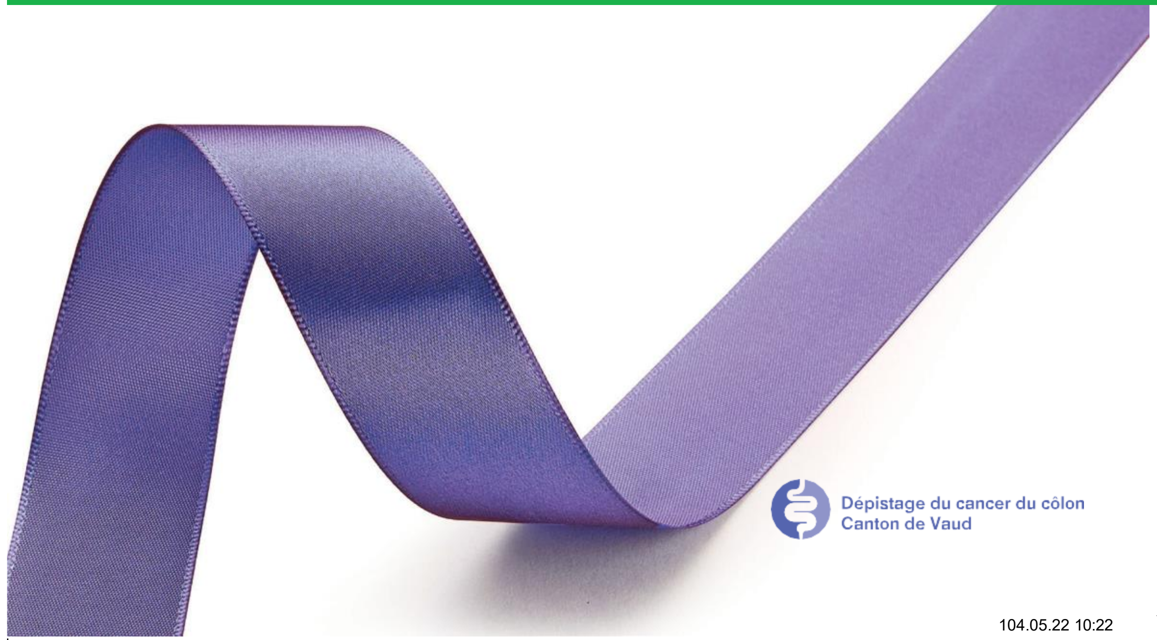

Dépistage du cancer du côlon
Canton de Vaud

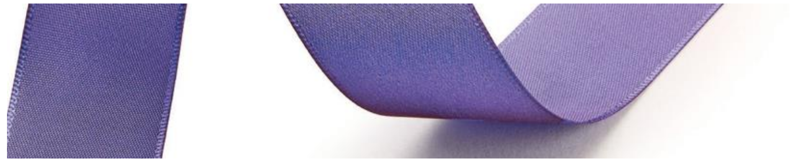

What do I need to know on colon cancer?

Most colon cancers appear after the age of 50. Small lumps (bumps) can appear in the colon: they are called polyps. Most of these are harmless, but a small number of them can slowly develop into cancer.

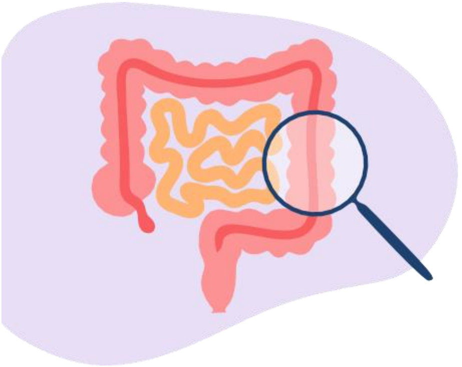

What are the positive effects of screening?

- Discovery of early stage cancers: lighter treatments with better chances of cure.
- Less risk of getting colon cancer.

My risk of developing colon cancer

Currently, the risk of developing colon cancer can be calculated for each individual.

To calculate your risk level, we used the answers to the questionnaire you filled out. This allows us to make a recommendation on which screening method is right for you.

According to our calculations, you are at low risk

We recommend a FIT test

According to our estimates 1 in 100 people with the same profile as you will get colon cancer in the next 15 years.

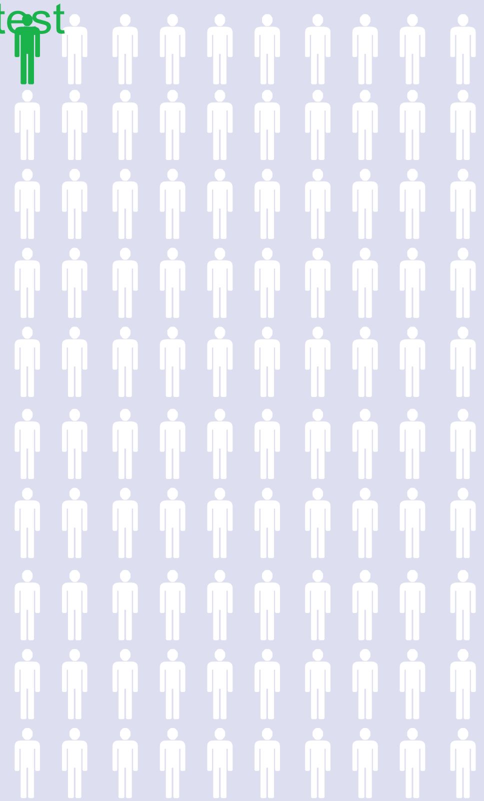

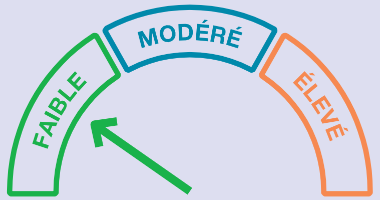

Should you get tested?

Yes, screening is recommended for people at low risk. The risk calculator is not perfect. Although your risk is low, it is not zero. The FIT test can find early cancers and bleeding polyps.

90 out of 100 people are cured if the cancer is found early.

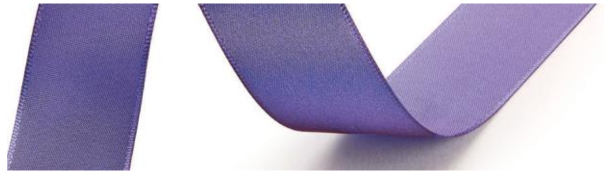

Why is the FIT test right for you?

The appropriate test for people at low risk is the FIT test, which looks for blood in the stool that is invisible to the naked eye. This test is done every two years and can find the vast majority of early cancers. This test does not require any special preparation, you can easily do it at home. It is covered by basic health insurance with no deductible.

What is the other screening option?

The other screening option is a colonoscopy. A doctor (gastroenterologist) explores your entire colon using a flexible tube with a camera. This is a very safe test, but it may cause side effects. We recommend it only in the case of a positive FIT test.

What should you look out for?

The risk of colon cancer increases with age. We recommend that you regularly discuss your risk level with your doctor. However, it is possible to reduce your risk of developing colon cancer by following a healthy lifestyle:

- **Eat fruits and vegetables daily**
- **Limit red meat consumption**
- **Have sufficient and regular physical activity**
- **Controllin g your weight**
- **Avoid tobacco and excess alcohol**

How to do a FIT test at home?

1

Go to a pharmacy:

A pharmacist explains the test to you.
The pharmacist gives you the test and its instructions.

Or

Make an appointment with your family doctor:

Your doctor gives you a prescription. You then pick up the test and its instructions at a pharmacy.

2

Take my FIT test at home

— First, you collect the stool on a special paper that you stick to the toilet seat.
— You then scrape the stool several times with the stem of the collection tube.
— You send the tube by mail to the laboratory.

3

You and your doctor will receive the test results by mail within a week.

The test is negative

The test showed nothing. This means that there is no sign of cancer in the stool. The test must be done again in 2 years.

► If you have any symptoms in the meantime, see your doctor right away.

The test is positive

The test shows blood in the stool. This does not necessarily mean that you have a cancer. But the cause of the blood in the stool must be sought.

► Make an appointment with your family doctor to arrange a colonoscopy.

12479_UNISANTE_Depliant_Colon_risquefaible.indd

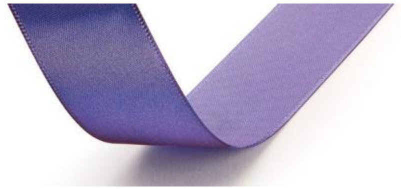

When should you see a doctor?

There is no test that detects all cancers at an early stage.
If you have any symptoms listed below, make an appointment with your family doctor:

- Blood in the stool, digestive disorders,
- Belly pain, diarrhea or constipation, more or less frequent stools than usual, unexplained weight loss,
- Long-lasting fatigue.

How is screening managed?

Both tests are covered by health insurance companies for people between 50 and 69 years old living in the canton of Vaud.
You will not pay a deductible. You will still have to pay the 10% deductible, i.e. about CHF 5 for the FIT test.
If you are over 69, ask your doctor if screening is still right for you.

For more information

Vaud cancer screening programs: www.pvdc.ch Swiss Cancer Screening : www.swisscancerscreening.ch Your family doctor or pharmacist.

v3.0_avril_2022

Unisanté
Route de Berne 113, 1010 Lausanne
Tel: 0848 990 990, Fax: 021 314 14 46
depistage.colon@unisante.ch, wwwpvdc.ch

unisanté
Centre universitaire de médecine générale et santé publique · Lausanne

Dépistage du cancer du côlon
Canton de Vaud

COLON CANCER SCREENING

From 50 years old I am informed

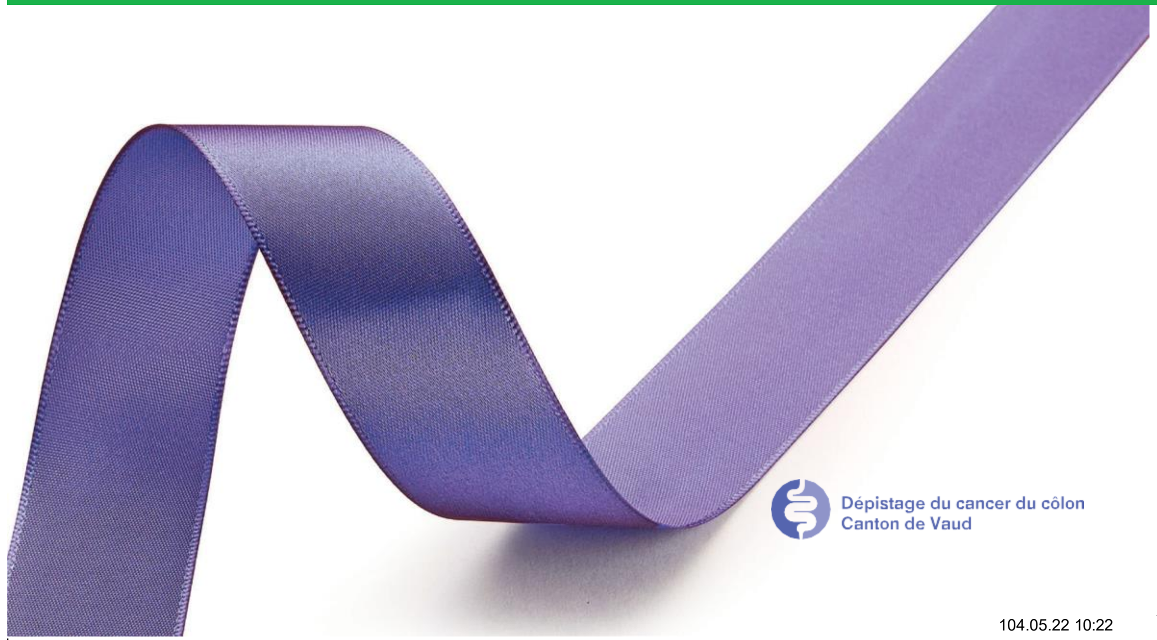

Dépistage du cancer du côlon
Canton de Vaud

104.05.22 10:22

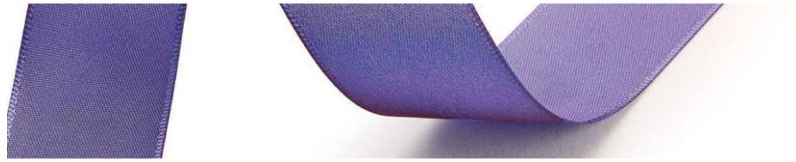

What do I need to know on colon cancer?

Most colon cancers appear after the age of 50. Small lumps (bumps) can appear in the colon: they are called polyps. Most of these are harmless, but a small number of them can slowly develop into cancer.

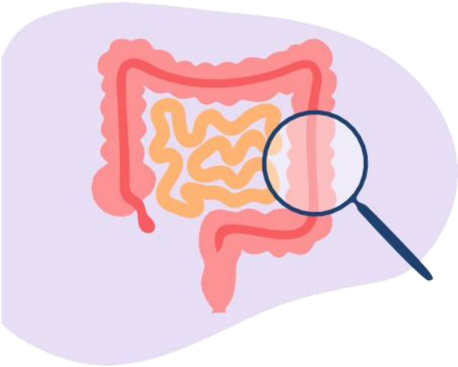

What are the positive effects of screening?

- Discovery of early stage cancers: lighter treatments with better chances of cure.
- Less risk of getting colon cancer.

My risk of developing colon cancer

Currently, the risk of developing colon cancer can be calculated for each individual.

To calculate your risk level, we used the answers to the questionnaire you filled out. This allows us to make a recommendation on which screening method is right for you.

According to our calculations, you are at low risk

We recommend a FIT test.

According to our estimates **2 out of 100 people** with the same profile as you will get colon cancer in **the next 15 years.**

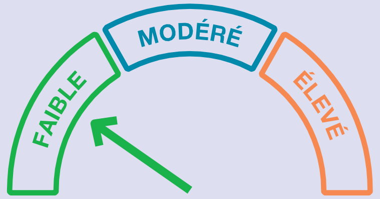

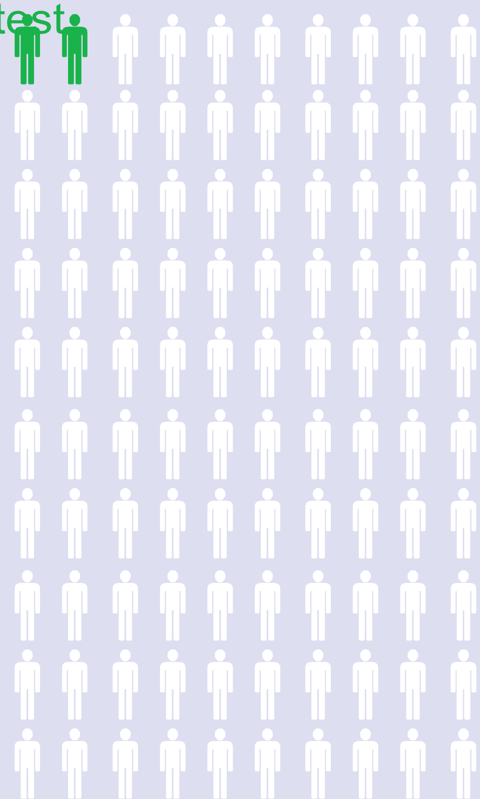

Should you get tested?

Yes, screening is recommended for people at low risk. The risk calculator is not perfect. Although your risk is low, it is not zero. The FIT test can find early cancers and bleeding polyps.

90 out of 100 people are cured if the cancer is found early.

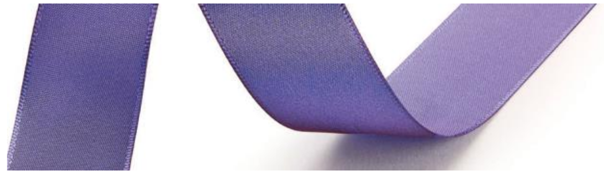

Why is the FIT test right for you?

The appropriate test for people at low risk is the FIT test, which looks for blood in the stool that is invisible to the naked eye. This test is done every two years and can find the vast majority of early cancers.
This test does not require any special preparation, you can easily do it at home. It is covered by basic health insurance with no deductible.

What is the other screening option?

The other screening option is a colonoscopy. A doctor (gastroenterologist) explores your entire colon using a flexible tube with a camera. This is a very safe test, but it may cause side effects. We recommend it only in the case of a positive FIT test.

What should you look out for?

The risk of colon cancer increases with age. We recommend that you regularly discuss your risk level with your doctor. However, it is possible to reduce your risk of developing colon cancer by following a healthy lifestyle:

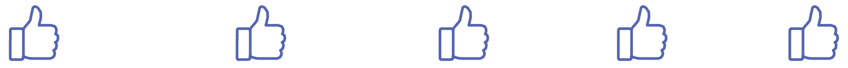

Eat fruits and vegetables daily

Limit red meat consumption

Have sufficient and regular physical activity

Controllin g your weight

Avoid tobacco and excess alcohol

You decide to do :

A FIT testA colonoscopy

1

Go to a pharmacy:
A pharmacist explains the test to you. The pharmacist gives you the test and its instructions f o r use. or

Make an appointment with your family doctor:
Your doctor gives you a prescription. You then pick up the test and its instructions at a pharmacy.

2

Take my FIT test at home
— First, you collect the stool on a special paper that you stick to the toilet seat.
— You then scrape the stool several times with the stem of the collection tube.
— You send the tube by mail to the laboratory.

3

You and your doctor will receive the test result by mail in less than a week.

1

Make an appointment with your family doctor.
He or she will advise you and prescribe a colonoscopy.

2

AGENDA

You will then be given an appointment with a gastroenterologist.

3

Before the exam, prepare carefully your intestine. This is not a pleasant step, but it is very important.
— Follow the recommended diet.
— Drink the recommended liquid (purge) to cleanse the bowel (usually the night before the exam and the day of the exam).

4

The gastroenterologist will the exam and will give you an oral result the same day.

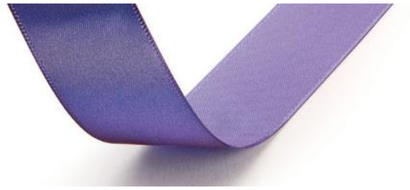

When should you see a doctor?

There is no test that detects all cancers at an early stage.
If you have any of the symptoms listed below, make an appointment with your family doctor:

- Blood in the stool, digestive disorders,
- Belly pain, diarrhea or constipation, more or less frequent stools than usual, unexplained weight loss,
- Long-lasting fatigue.

How is screening managed?

Both tests are covered by health insurance companies for people between 50 and 69 years old living in the canton of Vaud.
You will not pay a deductible. You will still have to pay the 10% deductible, i.e. about CHF 5 for the FIT test and between CHF 80 and CHF 160 for the colonoscopy.
If you are over 69, ask your doctor if screening is still right for you.

For more information
Vaud cancer screening programs: www.pvdc.ch Swiss Cancer Screening : www.swisscancerscreening.ch Your family doctor or pharmacist.

v3.0_avril_2022

Unisanté
Route de Berne 113, 1010 Lausanne
Tel: 0848 990 990, Fax: 021 314 14 46
depistage.colon@unisante.ch, wwwpvdc.ch

Dépistage du cancer du côlon
Canton de Vaud

unisanté
Centre universitaire de médecine générale et santé publique · Lausanne

COLON CANCER SCREENING

From 50 years old and over, I want to know more

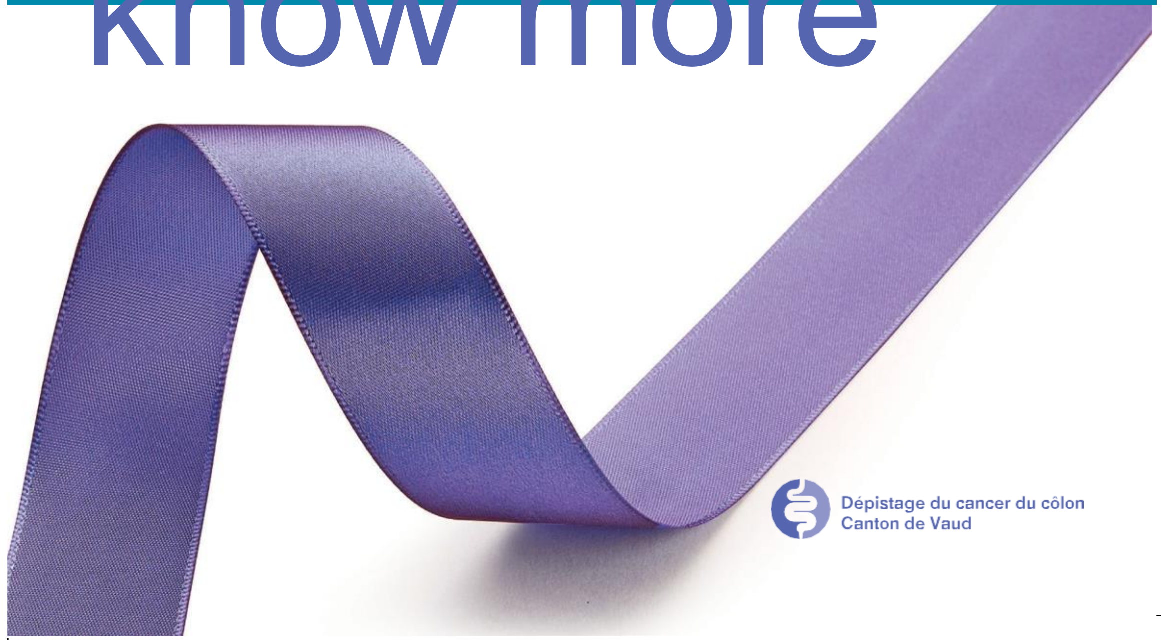

Dépistage du cancer du côlon
Canton de Vaud

12479_UNISANTE_Depliant_Colon_risquemodere.indd 104.05.22 10:24

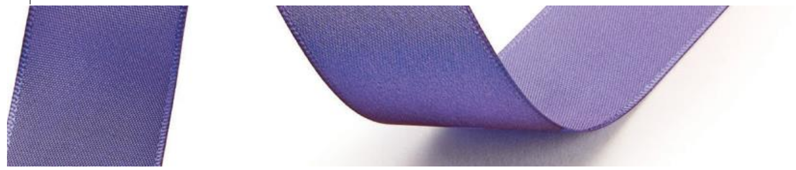

What do I need to know on colon cancer?

Most colon cancers appear after the age of 50. Small lumps (bumps) can appear in the colon: they are called polyps. Most of these are harmless, but a small number of them can slowly develop into cancer.

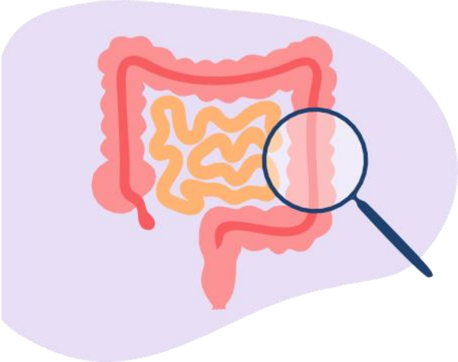

What are the positive effects of screening?

— Discovery of early stage cancers: lighter treatments with better chances of cure.
— Less risk of getting colon cancer.

My risk of developing colon cancer

Currently, the risk of developing colon cancer can be calculated for each individual.
To calculate your level of risk, we used the responses to the questionnaire you filled out. This allows us to make a recommendation on which screening method is right for you.

According to our calculations, you are at moderate risk

We recommend a FIT test or a colonoscopy

According to our estimates
3 out of 100 people with the same profile as you will get colon cancer in the next 15 years.

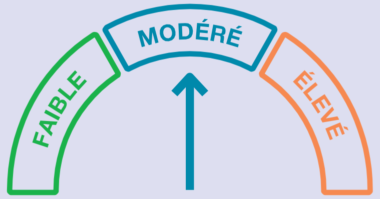

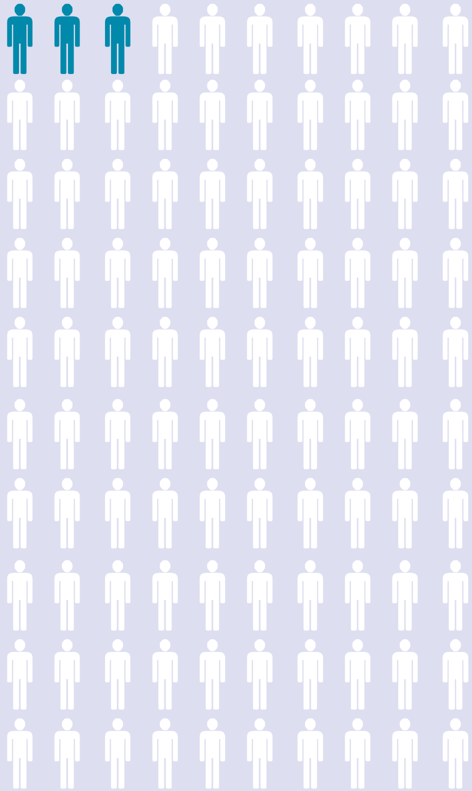

How can you lower your risk of getting colon cancer?

Make an appointment with your doctor to talk about it and get screened regularly.

90 out of 100 people are cured if the cancer is found early.

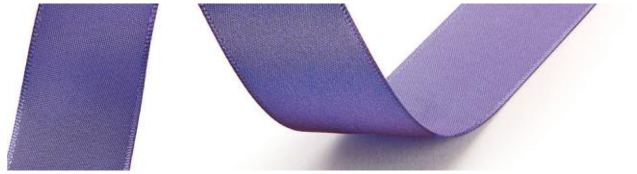

2 options are suitable for you

FIT Test

To look for blood in the stool that is invisible to the naked eye.
— Can be done easily and quickly at home.
— No preparation required.
— If the result is positive, a colonoscopy will be necessary.
— Waiting for a colonoscopy can be stressful.

Analysis to be repeated every 2 years.

Colonoscopy

To examine your intestine with a tube through the anus.
— This is the reference method for finding polyps.
— The doctor can remove polyps during the examination.
— A preparation of the intestine is necessary before the examination.
— About 2 in 1000 people will have a bowel perforation or major bleeding.
— If you are given a sedative (painkiller), you cannot drive.

Examination to be repeated every 10 years.

What should you look out for?

The risk of colon cancer increases with age. We recommend that you regularly discuss your risk level with your doctor. However, it is possible to reduce your risk of developing colon cancer by following a healthy lifestyle:

- **Eat daily fruits and vegetables**
- **Limit the red meat consumption**
- **Have a sufficient and regular physical activity**
- **Check your weight**
- **Avoid tobacco and alcohol abuse**

You decide to do :

A FIT testA colonoscopy

1

Go to a pharmacy:
A pharmacist explains the test to you. The pharmacist gives you the test and its instructions f o r use. or

Make an appointment with your family doctor:
Your doctor gives you a prescription. You then pick up the test and its instructions at a pharmacy.

2

Take my FIT test at home
— First, you collect the stool on a special paper that you stick to the toilet seat.
— You then scrape the stool several times with the stem of the collection tube.
— You send the tube by mail to the laboratory.

3

You and your doctor will receive the test result by mail in less than a week.

1

Make an appointment with your family doctor.
He or she will advise you and prescribe a colonoscopy.

2

AGENDA

You will then be given an appointment with a gastroenterologist.

3

Before the exam, prepare carefully your intestine. This is not a pleasant step, but it is very important.
— Follow the recommended diet.
— Drink the recommended liquid (purge) to cleanse the bowel (usually the night before the exam and the day of the exam).

4

The gastroenterologist will the exam and will give you an oral result the same day.

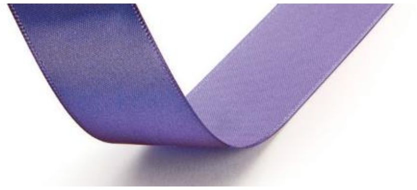

When should you see a doctor?

There is no test that detects all cancers at an early stage.
If you have any of the symptoms listed below, make an appointment with your family doctor:

- Blood in the stool, digestive disorders,
- Belly pain, diarrhea or constipation, more or less frequent stools than usual, unexplained weight loss,
- Long-lasting fatigue.

How is screening managed?

Both tests are covered by health insurance companies for people between 50 and 69 years old living in the canton of Vaud.
You will not pay a deductible. You will still have to pay the 10% deductible, i.e. about CHF 5 for the FIT test and between CHF 80 and CHF 160 for the colonoscopy.
If you are over 69, ask your doctor if screening is still right for you.

For more information
Vaud cancer screening programs: www.pvdc.ch Swiss Cancer Screening : www.swisscancerscreening.ch Your family doctor or pharmacist.

v3.0_avril_2022

Unisanté
Route de Berne 113, 1010 Lausanne
Tel: 0848 990 990, Fax: 021 314 14 46
depistage.colon@unisante.ch, wwwpvdc.ch

Dépistage du cancer du côlon
Canton de Vaud

unisanté
Centre universitaire de médecine générale et santé publique · Lausanne

COLON CANCER SCREENING

From 50 years old and over, I want to know more

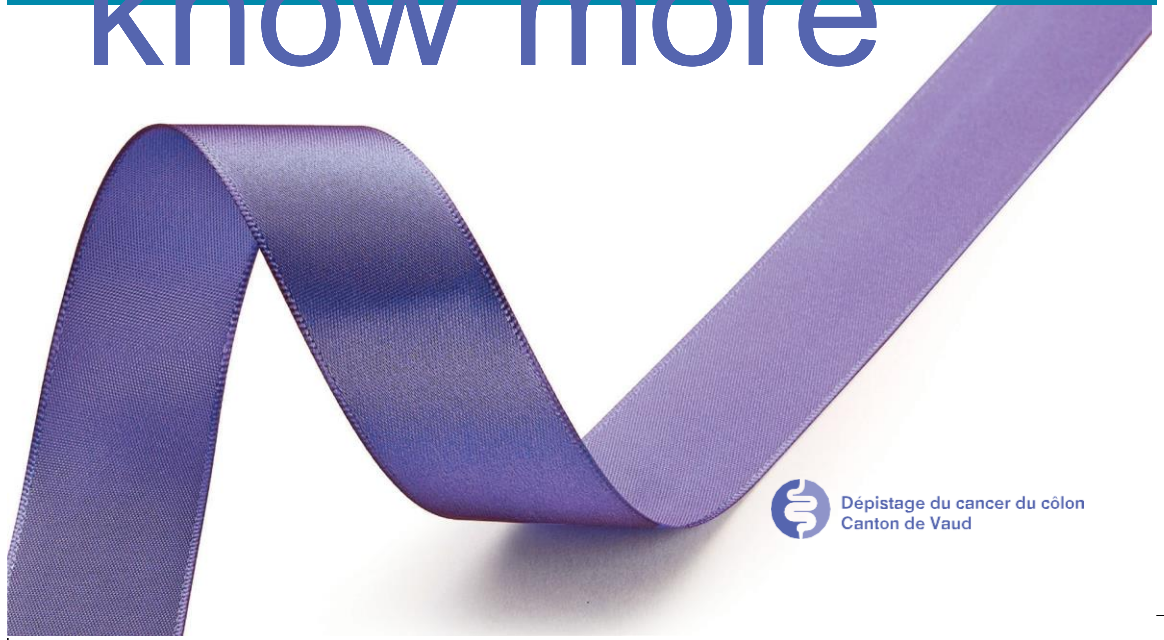

Dépistage du cancer du côlon
Canton de Vaud

12479_UNISANTE_Depliant_Colon_risquemodere.indd

104.05.22 10:24

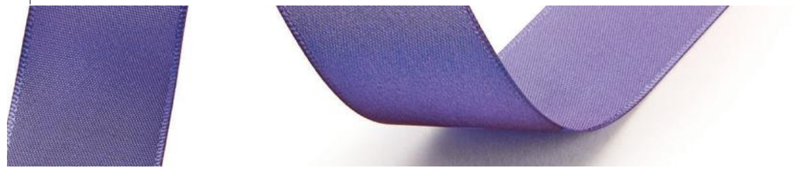

What do I need to know on colon cancer?

Most colon cancers appear after the age of 50. Small lumps (bumps) can appear in the colon: they are called polyps. Most of these are harmless, but a small number of them can slowly develop into cancer.

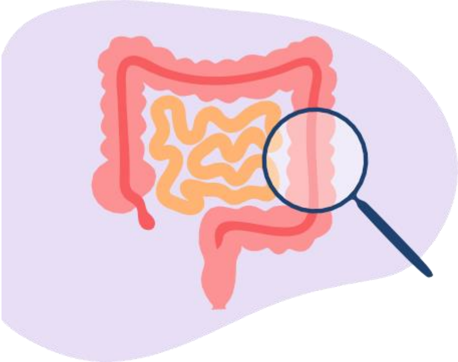

What are the positive effects of screening?

- Discovery of early stage cancers: lighter treatments with better chances of cure.
- Less risk of getting colon cancer.

My risk of developing colon cancer

Currently, the risk of developing colon cancer can be calculated for each individual.
To calculate your level of risk, we used the responses to the questionnaire you filled out. This allows us to make a recommendation on which screening method is right for you.

According to our calculations, you are at moderate risk

We recommend a FIT test or a colonoscopy

According to our estimates 4 out of 100 people with the same profile as you will get colon cancer in the next 15 years.

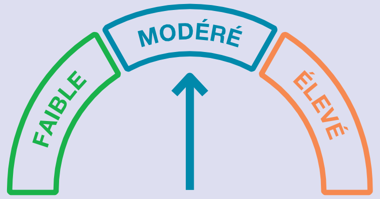

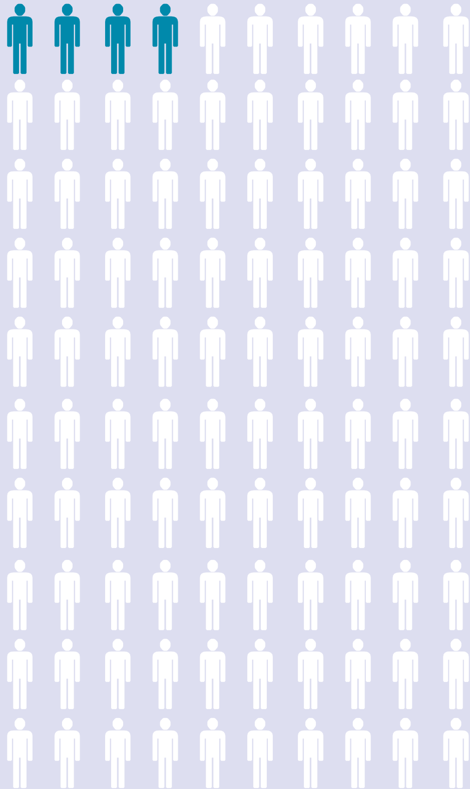

How can you lower your risk of getting colon cancer?

Make an appointment with your doctor to talk about it and get screened regularly.

90 out of 100 people are cured if the cancer is found early.

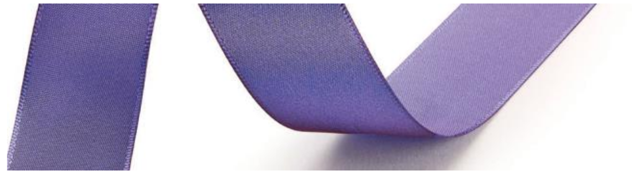

2 options are suitable for you

FIT Test

To look for blood in the stool that is invisible to the naked eye.
- Can be done easily and quickly at home.
- No preparation required.
- If the result is positive, a colonoscopy will be necessary.
- Waiting for a colonoscopy can be stressful.

Analysis to be repeated every 2 years.

Colonoscopy

To examine your intestine with a tube through the anus.
- This is the reference method for finding polyps.
- The doctor can remove polyps during the examination.
- A preparation of the intestine is necessary before the examination.
- About 2 in 1000 people will have a bowel perforation or major bleeding.
- If you are given a sedative (painkiller), you cannot drive.

Examination to be repeated every 10 years.

What should you look out for?

The risk of colon cancer increases with age. We recommend that you regularly discuss your risk level with your doctor. However, it is possible to reduce your risk of developing colon cancer by following a healthy lifestyle:

- **Eat daily fruits and vegetables**
- **Limit the red meat consumption**
- **Have a sufficient and regular physical activity**
- **Check your weight**
- **Avoid tobacco and alcohol abuse**

You decide to do :

A FIT testA colonoscopy

1

Go to a pharmacy:
A pharmacist explains the test to you. The pharmacist gives you the test and its instructions f o r use. or

Make an appointment with your family doctor:
Your doctor gives you a prescription. You then pick up the test and its instructions at a pharmacy.

2

Take my FIT test at home
— First, you collect the stool on a special paper that you stick to the toilet seat.
— You then scrape the stool several times with the stem of the collection tube.
— You send the tube by mail to the laboratory.

3

You and your doctor will receive the test result by mail in less than a week.

1

Make an appointment with your family doctor.
He or she will advise you and prescribe a colonoscopy.

2 AGENDA

You will then be given an appointment with a gastroenterologist.

3

Before the exam, prepare carefully your intestine. This is not a pleasant step, but it is very important.
— Follow the recommended diet.
— Drink the recommended liquid (purge) to cleanse the bowel (usually the night before the exam and the day of the exam).

4

The gastroenterologist will the exam and will give you an oral result the same day.

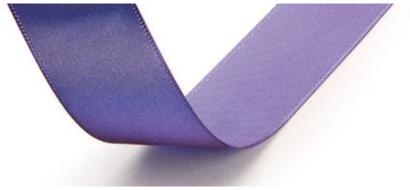

When should you see a doctor?

There is no test that detects all cancers at an early stage.
If you have any of the symptoms listed below, make an appointment with your family doctor:

- Blood in the stool, digestive disorders,
- Belly pain, diarrhea or constipation, more or less frequent stools than usual, unexplained weight loss,
- Long-lasting fatigue.

How is screening managed?

Both tests are covered by health insurance companies for people between 50 and 69 years old living in the canton of Vaud.
You will not pay a deductible. You will still have to pay the 10% deductible, i.e. about CHF 5 for the FIT test and between CHF 80 and CHF 160 for the colonoscopy.
If you are over 69, ask your doctor if screening is still right for you.

For more information
Vaud cancer screening programs: www.pvdc.ch Swiss Cancer Screening: www.swisscancerscreening.ch Your family doctor or pharmacist.

v3.0_avril_2022

Unisanté
Route de Berne 113, 1010 Lausanne
Tel: 0848 990 990, Fax: 021 314 14 46
depistage.colon@unisante.ch, wwwpvdc.ch

Dépistage du cancer du côlon
Canton de Vaud

unisanté
Centre universitaire de médecine générale et santé publique · Lausanne

COLON CANCER SCREENING

From 50 years old and over, I want to know more

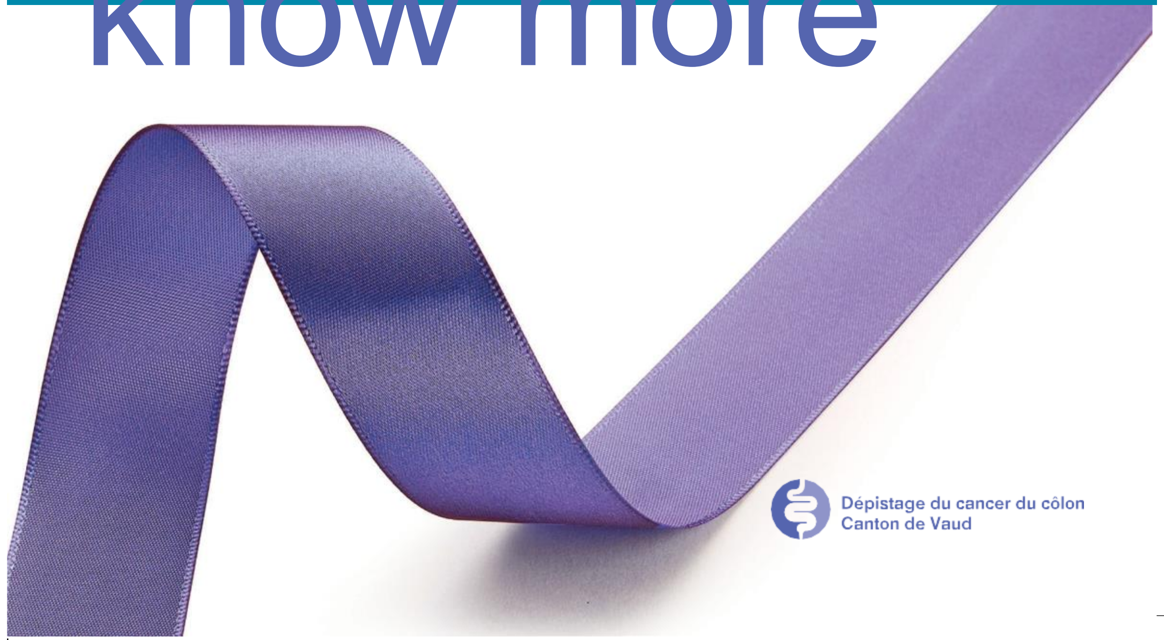

Dépistage du cancer du côlon
Canton de Vaud

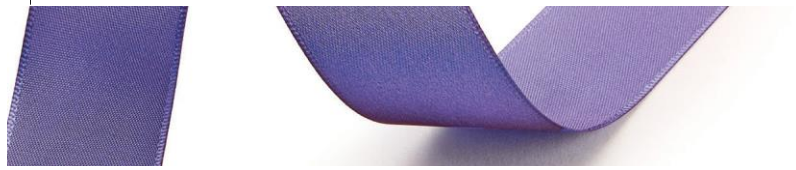

What do I need to know on colon cancer?

Most colon cancers appear after the age of 50. Small lumps (bumps) can appear in the colon: they are called polyps. Most of these are harmless, but a small number of them can slowly develop into cancer.

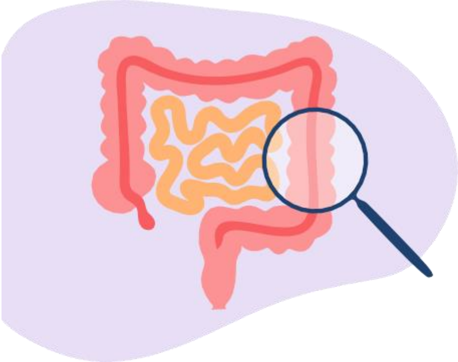

What are the positive effects of screening?

- Discovery of early stage cancers: lighter treatments with better chances of cure.
- Less risk of getting colon cancer.

My risk of developing colon cancer

Currently, the risk of developing colon cancer can be calculated for each individual.
To calculate your level of risk, we used the responses to the questionnaire you filled out. This allows us to make a recommendation on which screening method is right for you.

According to our calculations, you are at moderate risk

We recommend a FIT test or a colonoscopy

According to our estimates
5 out of 100 people with the same profile as you will get colon cancer in the next 15 years.

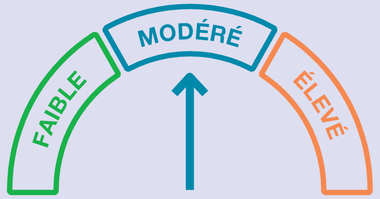

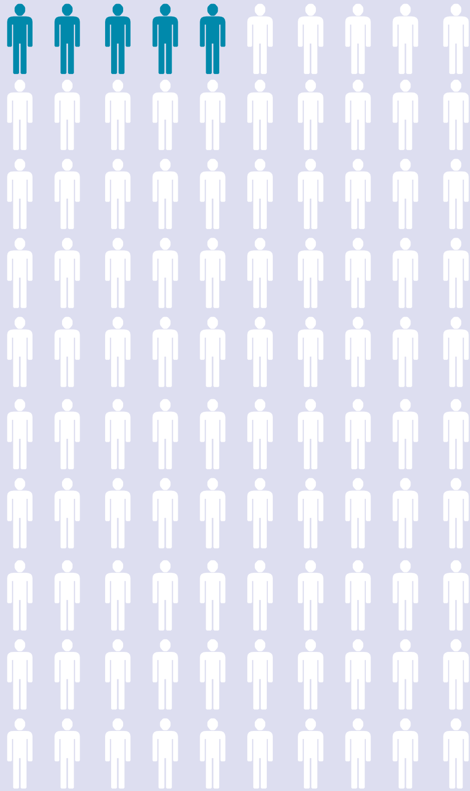

How can you lower your risk of getting colon cancer?

Make an appointment with your doctor to talk about it and get screened regularly.

90 out of 100 people are cured if the cancer is found early.

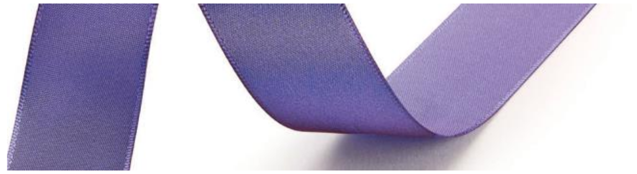

2 options are suitable for you

FIT Test

To look for blood in the stool that is invisible to the naked eye.

- Can be done easily and quickly at home.
- No preparation required.
- If the result is positive, a colonoscopy will be necessary.
- Waiting for a colonoscopy can be stressful.

Analysis to be repeated every 2 years.

Colonoscopy

To examine your intestine with a tube through the anus.

- This is the reference method for finding polyps.
- The doctor can remove polyps during the examination.
- A preparation of the intestine is necessary before the examination.
- About 2 in 1000 people will have a bowel perforation or major bleeding.
- If you are given a sedative (painkiller), you cannot drive.

Examination to be repeated every 10 years.

What should you look out for?

The risk of colon cancer increases with age. We recommend that you regularly discuss your risk level with your doctor. However, it is possible to reduce your risk of developing colon cancer by following a healthy lifestyle:

- **Eat daily fruits and vegetables**
- **Limit the red meat consumption**
- **Have a sufficient and regular physical activity**
- **Check your weight**
- **Avoid tobacco and alcohol abuse**

What are the steps for a colonoscopy?

1 **Make an appointment with your family doctor:**
He or she will advise you and prescribe a colonoscopy.

2 You will then be given an appointment with a gastroenterologist.

AGENDA

3 Before the exam, carefully prepare your bowel. This is not a pleasant step, but it is very important.
— Follow the recommended diet.
— Drink the recommended liquid (purge) to cleanse the bowel (usually the night before the exam and the day of the exam).

4 **The gastroenterologist will perform the examination and give you an oral result the same day.**

Colonoscopy showed nothing

— Important: the colonoscopy must be repeated 10 years later.
— If you have any symptoms in the meantime, see your doctor right away.

Colonoscopy shows polyps

— The doctor usually removes the polyps during the colonoscopy.
— If the doctor suspects cancer, he or she will explain to you on the same day what to do.

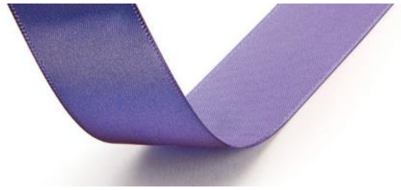

When should you see a doctor?

There is no test that detects all cancers at an early stage.
If you have any of the symptoms listed below, make an appointment with your family doctor:

- Blood in the stool, digestive disorders,
- Belly pain, diarrhea or constipation, more or less frequent stools than usual, unexplained weight loss,
- Long-lasting fatigue.

How is screening managed?

Both tests are covered by the health insurance companies for people between 50 and 69 years old living in the canton of Vaud. You will not pay a deductible. You will have to pay the 10% deductible, i.e. CHF 80 to CHF 160 for the colonoscopy.
If you are over 69, ask your doctor if screening is still right for you.

For more information

Vaud cancer screening programs: www.pvdc.ch Swiss Cancer Screening : www.swisscancerscreening.ch Your family doctor or pharmacist.

v3.0_avril_2022

unisanté
Centre universitaire de médecine générale et santé publique · Lausanne

Unisanté
Route de Berne 113, 1010 Lausanne
Tel: 0848 990 990, Fax: 021 314 14 46
depistage.colon@unisante.ch, wwwpvdc.ch

Dépistage du cancer du côlon
Canton de Vaud

COLON CANCER SCREENING

From 50 years old I am informed

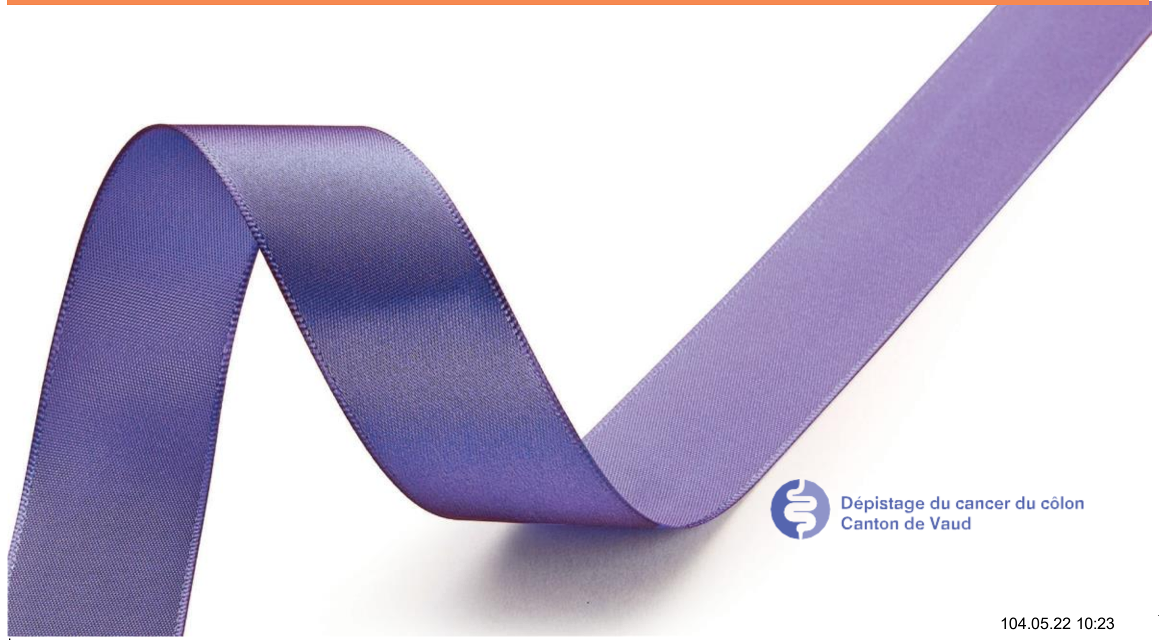

Dépistage du cancer du côlon
Canton de Vaud

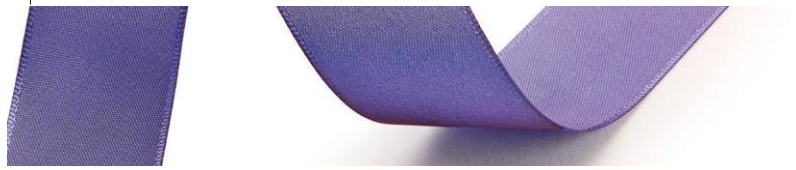

What do I need to know on colon cancer?

Most colon cancers appear after the age of 50. Small lumps (bumps) can appear in the colon: they are called polyps. Most of these are harmless, but a small number of them can slowly develop into cancer.

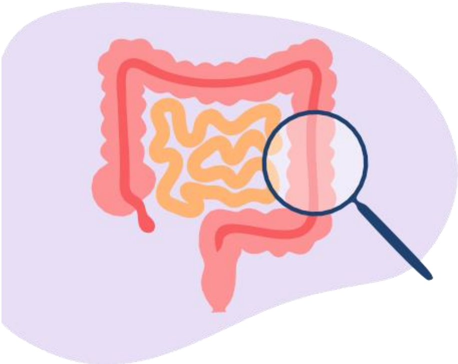

What are the positive effects of screening?

- Discovery of early stage cancers: lighter treatments with better chances of cure.
- Less risk of getting colon cancer.

My risk of developing colon cancer

Currently, the risk of developing colon cancer can be calculated for each individual.

To calculate your risk level, we used the answers to the questionnaire you filled out. This allows us to make a recommendation on which screening method is right for you.

According to our calculations, you are at high risk

We recommend a colonoscopy

According to our estimates 6 out of 100 people with the same profile as you will get colon cancer in the next 15 years.

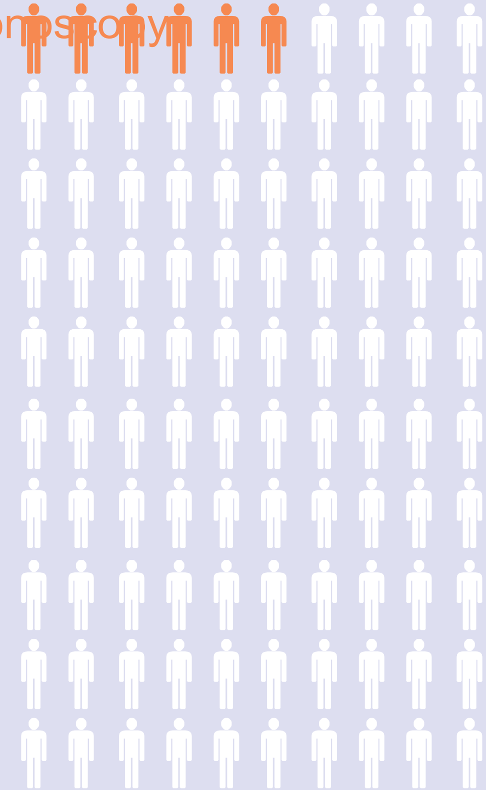

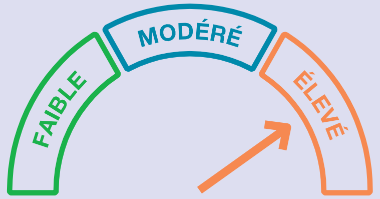

How can you lower your risk of getting colon cancer?

Make an appointment with your doctor to discuss this.
Get regular screening with colonoscopy.

90 out of 100 people are cured if the cancer is found early.

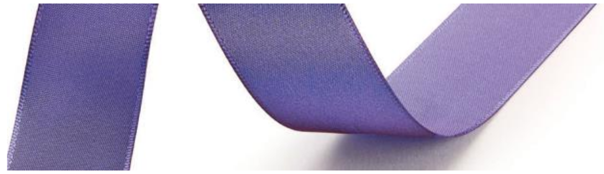

Why is a colonoscopy right for you?

A colonoscopy is performed by a gastroenterologist. During the examination, the doctor inserts a tube into the intestine through the anus. A small camera at the end of the tube allows the doctor to observe the entire intestine. If the doctor finds any polyps, he or she removes them during the examination. This greatly reduces the risk of getting cancer.
Screening with colonoscopy is done every 10 years. It is covered by basic health insurance with no deductible.

What is the other screening option?

The other screening option is the FIT test, which looks for blood in the stool that is invisible to the naked eye. This test is done every two years and can find the vast majority of early cancers. However, it does not find and remove polyps. This test is recommended for people at low risk.

What should you look out for?

The risk of colon cancer increases with age. We recommend that you regularly discuss your risk level with your doctor. However, it is possible to reduce your risk of developing colon cancer by following a healthy lifestyle:

- Eat fruits and vegetables daily
- Limit red meat consumption
- Have sufficient and regular physical activity
- Controlling your weight
- Avoid tobacco and excess alcohol

What are the steps for a colonoscopy?

1 **Make an appointment with your family doctor:**
He or she will advise you and prescribe a colonoscopy.

2 You will then be given an appointment with a gastroenterologist.

AGENDA

3 Before the exam, carefully prepare your bowel. This is not a pleasant step, but it is very important.
— Follow the recommended diet.
— Drink the recommended liquid (purge) to cleanse the bowel (usually the night before the exam and the day of the exam).

4 **The gastroenterologist will perform the examination and give you an oral result the same day.**

Colonoscopy showed nothing

— Important: the colonoscopy must be repeated 10 years later.
— If you have any symptoms in the meantime, see your doctor right away.

Colonoscopy shows polyps

— The doctor usually removes the polyps during the colonoscopy.
— If the doctor suspects cancer, he or she will explain to you on the same day what to do.

12479_UNISANTE_Depliant_Colon_risqueeleve.indd

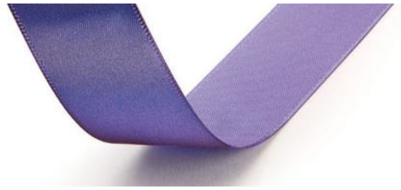

When should you see a doctor?

There is no test that detects all cancers at an early stage.
If you have any of the symptoms listed below, make an appointment with your family doctor:

- Blood in the stool, digestive disorders,
- Belly pain, diarrhea or constipation, more or less frequent stools than usual, unexplained weight loss,
- Long-lasting fatigue.

How is screening managed?

Both tests are covered by the health insurance companies for people between 50 and 69 years old living in the canton of Vaud. You will not pay a deductible. You will have to pay the 10% deductible, i.e. CHF 80 to CHF 160 for the colonoscopy.
If you are over 69, ask your doctor if screening is still right for you.

For more information

Vaud cancer screening programs: www.pvdc.ch Swiss Cancer Screening: www.swisscancerscreening.ch Your family doctor or pharmacist.

v3.0_avril_2022

unisanté
Centre universitaire de médecine générale et santé publique · Lausanne

Unisanté
Route de Berne 113, 1010 Lausanne
Tel: 0848 990 990, Fax: 021 314 14 46
depistage.colon@unisante.ch, wwwpvdc.ch

Dépistage du cancer du côlon
Canton de Vaud

COLON CANCER SCREENING

From 50 years old I am informed

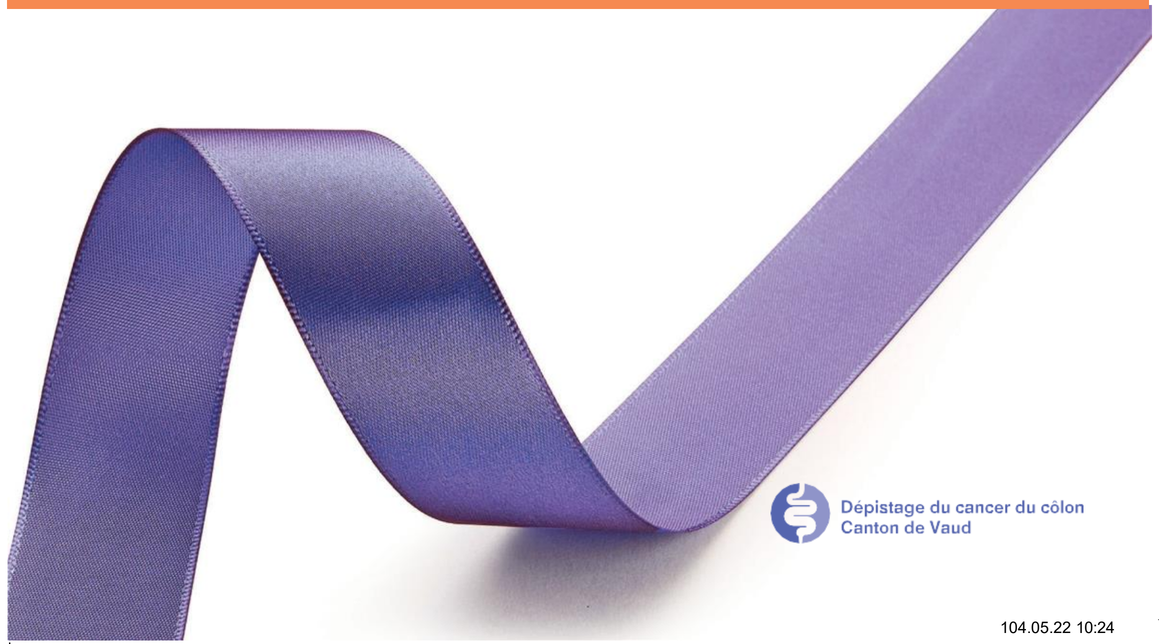

Dépistage du cancer du côlon
Canton de Vaud

104.05.22 10:24

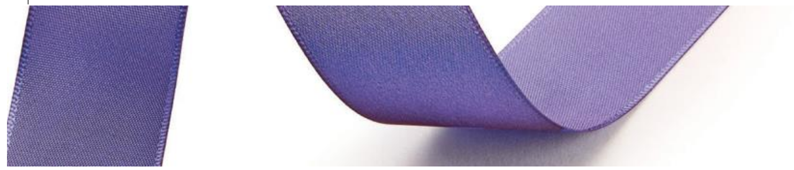

What do I need to know on colon cancer?

Most colon cancers appear after the age of 50. Small lumps (bumps) can appear in the colon: they are called polyps. Most of these are harmless, but a small number of them can slowly develop into cancer.

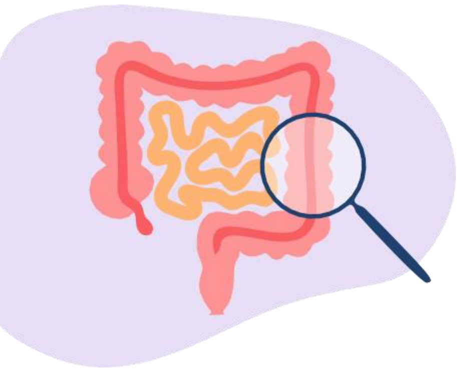

What are the positive effects of screening?

- Discovery of early stage cancers: lighter treatments with better chances of cure.
- Less risk of getting colon cancer.

My risk of developing colon cancer

Currently, the risk of developing colon cancer can be calculated for each individual.

To calculate your risk level, we used the answers to the questionnaire you filled out. This allows us to make a recommendation on which screening method is right for you.

According to our calculations, you are at high risk

We recommend a colonoscopy

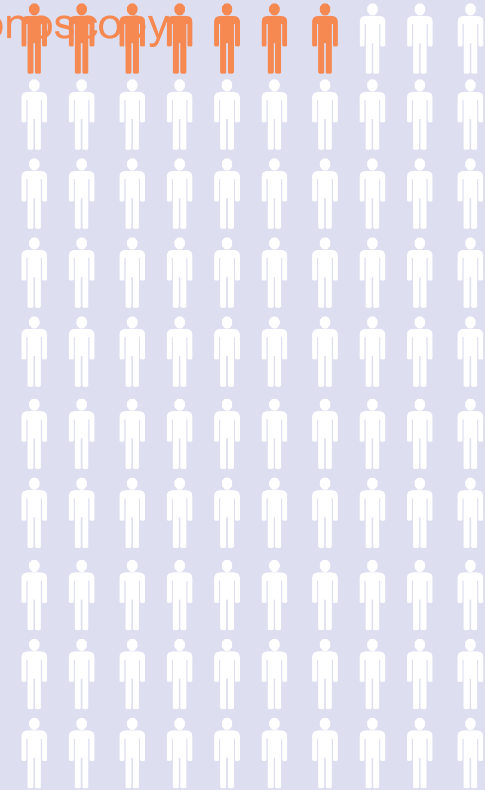

According to our estimates 7 out of 100 people with the same profile as you will get colon cancer in the next 15 years.

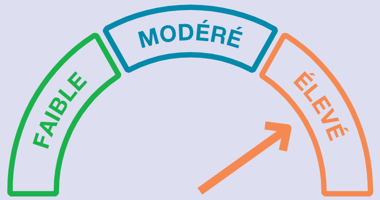

How can you lower your risk of getting colon cancer?

Make an appointment with your doctor to discuss this.
Get regular screening with colonoscopy.

90 out of 100 people are cured if the cancer is found early.

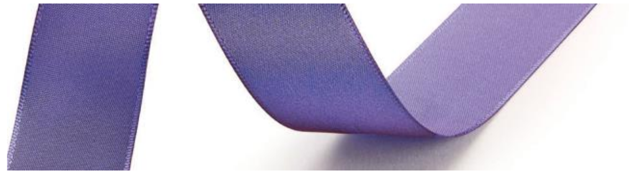

Why is a colonoscopy right for you?

A colonoscopy is performed by a gastroenterologist. During the examination, the doctor inserts a tube into the intestine through the anus. A small camera at the end of the tube allows the doctor to observe the entire intestine. If the doctor finds any polyps, he or she removes them during the examination. This greatly reduces the risk of getting cancer.
Screening with colonoscopy is done every 10 years. It is covered by basic health insurance with no deductible.

What is the other screening option?

The other screening option is the FIT test, which looks for blood in the stool that is invisible to the naked eye. This test is done every two years and can find the vast majority of early cancers. However, it does not find and remove polyps. This test is recommended for people at low risk.

What should you look out for?

The risk of colon cancer increases with age. We recommend that you regularly discuss your risk level with your doctor. However, it is possible to reduce your risk of developing colon cancer by following a healthy lifestyle:

- **Eat fruits and vegetables daily**
- **Limit red meat consumption**
- **Have sufficient and regular physical activity**
- **Controlling your weight**
- **Avoid tobacco and excess alcohol**

Colon cancer screening

Information leaflet

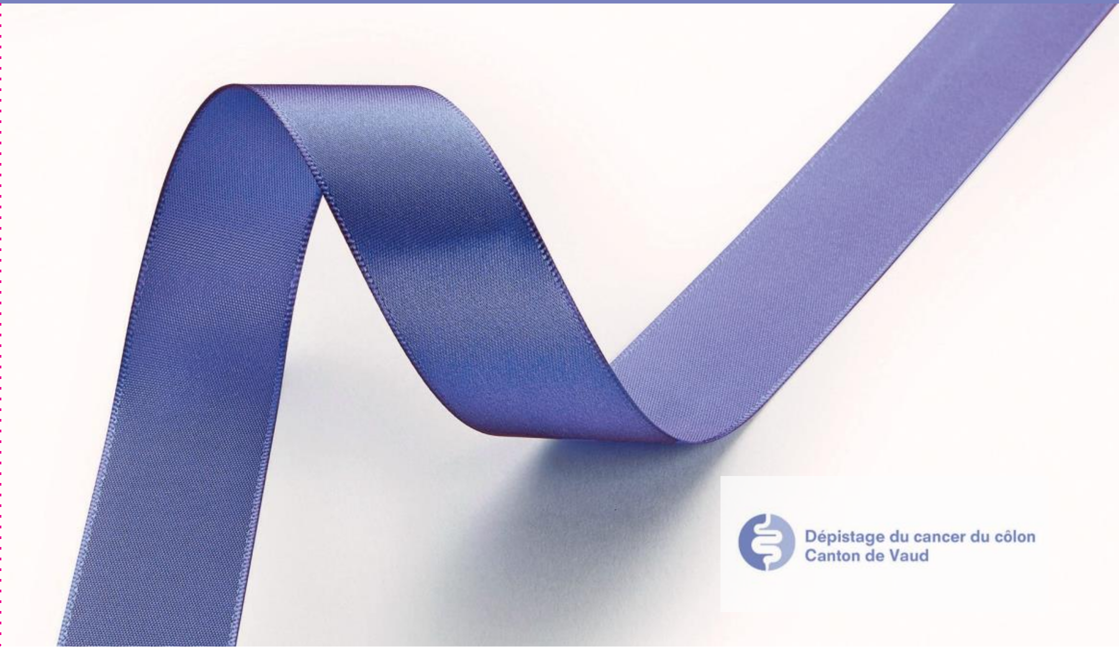

Dépistage du cancer du côlon
Canton de Vaud

What is colon cancer?

Most colon cancers appear after the age of 50.
Small balls (lumps) can appear in the colon: they are called polyps. Most polyps are harmless. A small number of them can slowly develop into cancer.

What are the positive effects of screening?

- Fewer people will get colon cancer.
- Discovery of early stage cancers: less severe treatments with better chances of cure.
- Fewer people will die of colon cancer.

Number of people who die of colon cancer before the age of 80

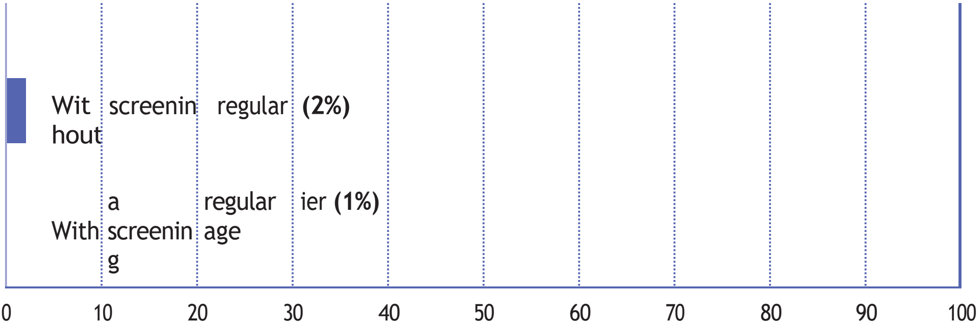

Screening tests

There are two main tests: the FIT test (test for blood in the stool) and the colonoscopy. You can choose to have the FIT test **or** the colonoscopy.

**FIT Test**<br>**Blood stool test**	**Colonoscopy**<br>**Endoscopic examination of the colon**
Early on, colon cancer usually causes small amounts of bleeding in the stool. The blood is not visible to the naked eye. The FIT test can detect these traces of blood in the stool.	A specialized doctor (gastroenterologist) performs the colonoscopy. During the examination, the doctor inserts a tube into the intestine through the anus. A small camera at the end of the tube makes it possible to observe the entire large intestine.

You decide to take the FIT test

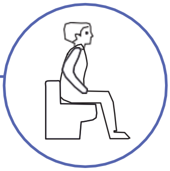

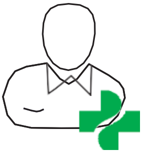

Go to a pharmacy:

- A pharmacist explains the test to you.
- The pharmacist gives you the test and its instructions.

OR

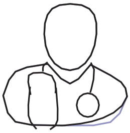

Make an appointment with your family doctor:

- Your doctor gives you a prescription.
- You then go to a pharmacy to get the test and its instructions.

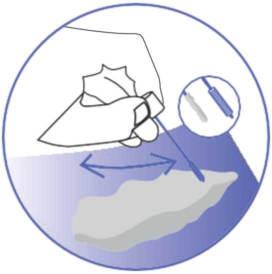

How do I take the FIT test at home?

- First, you collect the stool on a special paper, which you stick to the toilet seat.
- You then scrape the stool several times with the stem of the collection tube.
- You send the tube by mail to the laboratory.

You and your doctor will receive the test results by mail within a week.

The test showed nothing:
The test is negative. This means that there is no sign of cancer in the stool.

- The test must be repeated in 2 years.
- If you have any symptoms in the meantime, see your doctor right away.

The test shows blood in the stool:
The test is positive. This does not necessarily mean that you have cancer. But you should look for the cause of the blood in the stool.

- Make an appointment with your family doctor to arrange a colonoscopy.

You decide to have the colonoscopy

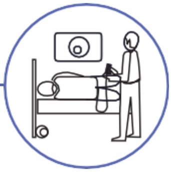

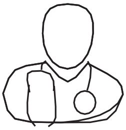

Make an appointment with your family doctor.
He or she will advise you and prescribe a colonoscopy.

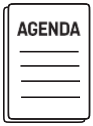

You will then be given an appointment for a colonoscopy with a gastroenterologist.

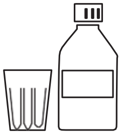

Before the examination, carefully prepare your bowel.
This is a not very pleasant but very important step.

- Follow the recommended diet.
- Drink the recommended liquid (purge) to cleanse the bowel (usually the night before the exam and the day of the exam).

You will receive a sedative medication. The gastroenterologist will perform the examination and give you an oral result the same day.

Colonoscopy showed nothing:

- Important: colonoscopy must be repeated 10 years later.
- If you have any symptoms in the meantime, see your doctor right away.

Colonoscopy shows polyps or cancer:

- The doctor usually removes the polyps during the colonoscopy.
- If the doctor finds cancer, he or she will explain to you on the same day what to do.

What are the advantages and disadvantages of the two tests?

	FIT Test 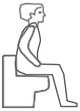	Colonoscopy 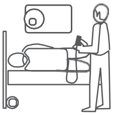
**What are the benefits?** ➕	• The test is done easily and quickly at home.<br>• No preparation required.<br>• You avoid a colonoscopy if the FIT is negative.	• This is the reference method for finding polyps.<br>• The doctor can remove polyps during the examination.
**What are the drawbacks?** ➖	• If the result is positive, a colonoscopy will still be necessary.<br>• Waiting for a colonoscopy can be stressful.	• A diet and bowel preparation are necessary before the examination.<br>• About 2 in 1000 people (0.2%) will have a severe problem (bowel perforation or major bleeding).<br>• If you are given a sedative (painkiller), you cannot drive for 12 to 24 hours.
**How often should you be tested?**	• Every two years	• Every ten years.
**Can we trust this test?**	• The test is reliable if it is repeated every two years.	• The test is reliable if you follow the preparation at home.

Limitations of screening

There is always a risk of cancer between screening tests. It is therefore important to go to your doctor immediately if you have the following symptoms:

- Blood in the stool.
- Digestive disorders (pain, diarrhea or constipation).
- Having more or less frequent bowel movements than usual
- Having a weight loss, for no known reason.
- Feeling tired for no known reason.

How do I get tested?

If you are between the ages of 50 and 69, complete this chart first:

Questions	Yes	No
Do you have any of the following symptoms: blood in the stool, digestive problems, unusual bowel movements (pain, diarrhea or constipation), more or less frequent bowel movements than usual, unexplained weight loss, lasting fatigue?	☐	☐
Have you ever had colon cancer or polyps?	☐	☐
Did anyone in your immediate family (parents, brother, sister) have colon cancer before age 60?	☐	☐
Are you being followed for inflammatory bowel disease?	☐	☐

Did you answer NO to all the questions?

- If you want to take the FIT test, you can get the test from a pharmacist.
- If you would like to have the colonoscopy or have your doctor's advice to decide, make an appointment with him or her.

Did you answer YES to one or more questions?

- Make an appointment with your doctor to find out how to proceed.

How is screening managed?

Both tests are covered by health insurance companies for people between 50 and 69 years old living in the canton of Vaud. You will not pay any deductible. You will still have to pay the 10% deductible.

For more information

Complete brochure: **www.unisante.ch/colon/brochure.pdf**
Vaud cancer screening programs: **www.pvdc.ch** Swiss Cancer Screening: **www.swisscancerscreening.ch**
Your family doctor or pharmacist

Dépistage du cancer du côlon
Canton de Vaud

Unisanté
Route de Berne 113, 1010 Lausanne
Tel: 0848 990 990, Fax: 021 314 14 46
depistage.colon@unisante.ch, www.pvdc.ch

unisanté
Centre universitaire de médecine générale et santé publique • Lausanne

Design/editing of this brochure:
Unisanté - Lausanne in partnership with Swiss Cancer Screening, June 2021
