# Supplementary material for: Colorectal Cancer Screening Decision Based on Predicted Risk: Protocol for a Pilot Randomized Controlled Trial
Source: JMIR Res Protoc. 2023 Sep 7;12:e46865. doi: 10.2196/46865 (PMC10514773; doi:10.2196/46865)
Supplement: Multimedia Appendix 5 [file resprot_v12i1e46865_app5.pdf]

Comment faire un test FIT chez soi ?

1

Allez dans une pharmacie:
Un pharmacien vous explique le test.
Le pharmacien vous donne le test et son mode d'emploi.

Ou

Prenez rendez-vous chez votre médecin de famille:
Votre médecin vous donne une ordonnance.
Vous allez ensuite chercher le test et son mode d'emploi dans une pharmacie.

2

Faire mon test FIT à domicile
— Vous collectez d'abord les selles sur un papier spécial à coller à la lunette des toilettes.
— Vous grattez ensuite les selles plusieurs fois avec la tige du tube de collecte.
— Vous envoyez le tube par la poste au laboratoire.

3

Vous et votre médecin recevrez le résultat du test par courrier en moins d'une semaine.

Le test est négatif Le test n'a rien montré. Cela veut dire qu'il n'y a pas de signe de cancer dans les selles. Le test doit être refait dans 2 ans.

▶ Si vous avez des symptômes entre-temps, consultez votre médecin sans tarder.

Le test est positif Le test montre du sang dans les selles. Ceci ne veut pas forcément dire que vous avez un cancer. Mais il faut rechercher la cause du sang dans les selles.

▶ Prenez rendez-vous chez votre médecin de famille pour organiser une coloscopie.

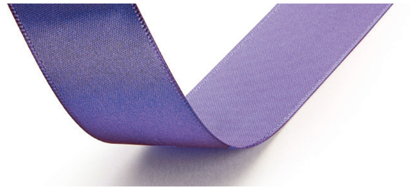

Quand devrez-vous consulter un médecin ?

Il n'existe pas de test qui détecte à 100% tous les cancers à un stade précoce.
Si vous avez un des symptômes listés ci-dessous, prenez un rendez-vous avec votre médecin de famille:

- Sang dans les selles,
- Troubles digestifs,
- Douleurs dans le ventre, diarrhée ou constipation,
- Selles plus ou moins fréquentes que d'habitude,
- Perte de poids inexpliquée,
- Fatigue durable.

Comment le dépistage est-il pris en charge ?

Les deux tests sont pris en charge par les caisses maladie pour les personnes entre 50 et 69 ans habitant dans le canton de Vaud.
Vous ne payerez pas de franchise. Il vous restera à payer la quotepart de 10%, c'est-à-dire environ CHF 5.– pour le test FIT.
Si vous avez plus de 69 ans, demandez à votre médecin pour savoir si le dépistage est toujours adapté pour vous.

Pour plus d'informations
Programmes vaudois de dépistage du cancer: www.pvdc.ch
Swiss Cancer Screening: www.swisscancerscreening.ch
Votre médecin de famille ou votre pharmacien.

v3.0_avril_2022

unisanté
Centre universitaire de médecine générale et santé publique • Lausanne

Unisanté
Route de Berne 113, 1010 Lausanne
Tel: 0848 990 990, Fax: 021 314 14 46
depistage.colon@unisante.ch, wwwpvdc.ch

Dépistage du cancer du côlon
Canton de Vaud

DÉPISTAGE DU CANCER DU CÔLON

À partir de 50 ans je m'informe

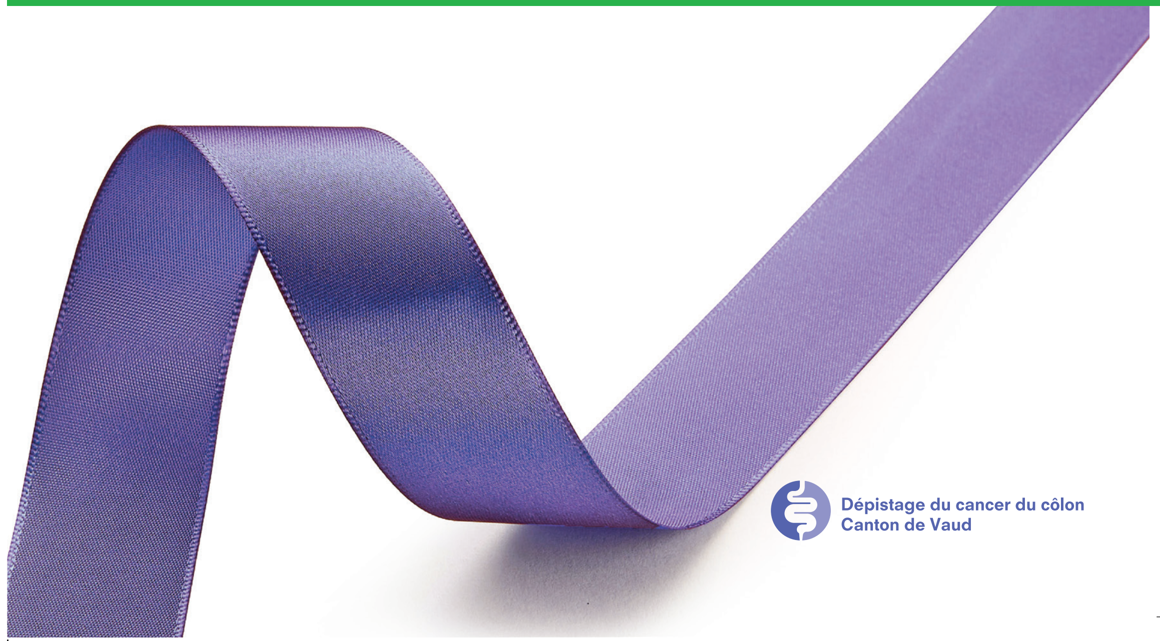

Dépistage du cancer du côlon
Canton de Vaud

12479_UNISANTE_Depliant_Colon_risquefaible.indd 1
04.05.22 10:22

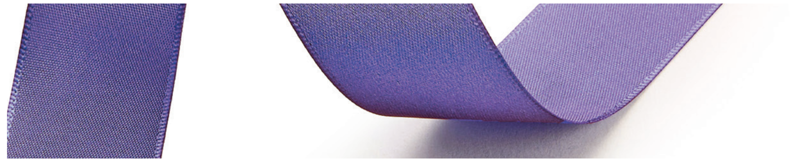

Que dois-je savoir sur le cancer du côlon ?

La plupart des cancers du côlon apparaissent après l'âge de 50 ans. De petites grosseurs (bosses) peuvent apparaître dans le côlon : on les appelle polypes. La plupart de ceux-ci sont sans danger, mais un faible nombre d'entre eux peut lentement évoluer en cancer.

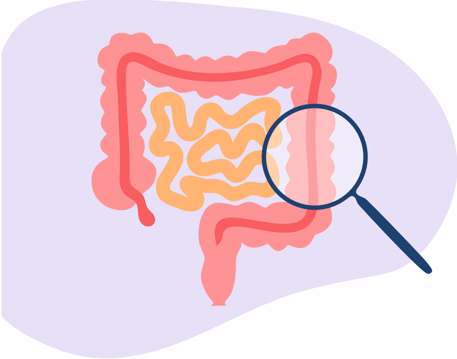

Quels sont les effets positifs du dépistage ?

— Découverte de cancers débutants : traitements allégés avec de meilleures chances de guérison.
— Moins de risque d'avoir un cancer du côlon.

Mon risque de développer un cancer du côlon

Actuellement, le risque d'avoir un cancer du côlon peut être calculé pour chaque personne.
Pour calculer votre niveau de risque, nous avons utilisé les réponses au questionnaire que vous avez rempli. Cela permet de faire une recommandation sur la méthode de dépistage qui est adaptée pour vous.

Selon nos calculs, vous êtes à faible risque

Nous vous recommandons un test FIT

D'après nos estimations **1 personne sur 100 ayant le même profil que vous, aura un cancer du côlon dans les 15 prochaines années.**

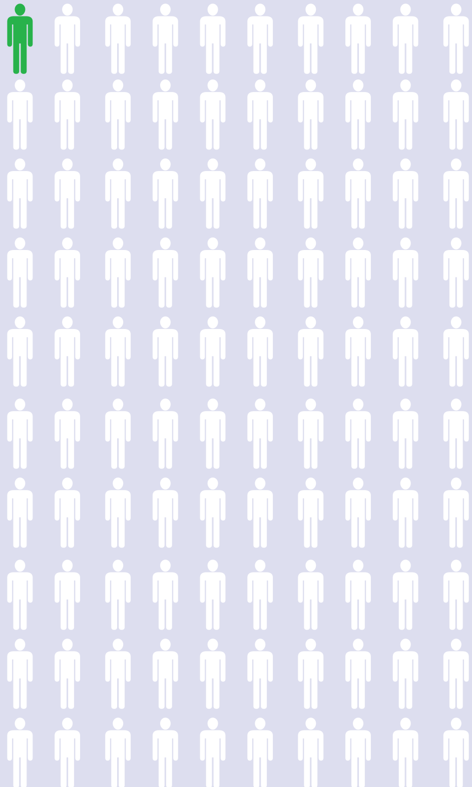

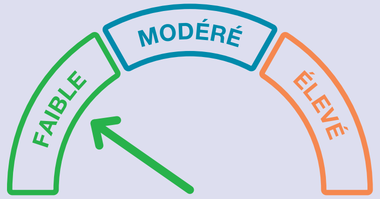

Devez-vous vous faire dépister ?

Oui, le dépistage est recommandé pour les personnes à faible risque. Le calculateur du risque n'est pas parfait. Bien que votre risque soit faible, il n'est pas nul. Le test FIT peut trouver des cancers débutants et des polypes qui saignent.

90 personnes sur 100 guérissent si le cancer est trouvé tôt.

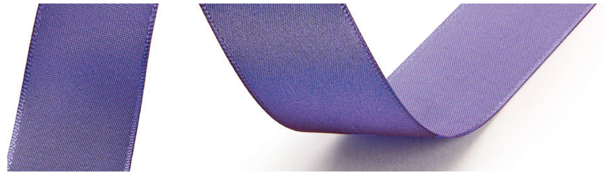

Pourquoi le test FIT est adapté pour vous ?

Le test adapté pour les personnes à faible risque est le test FIT qui sert à chercher du sang invisible à l'œil nu dans les selles. Ce test se fait tous les deux ans et permet de trouver la grande majorité de cancers débutants.
Ce test ne nécessite aucune préparation particulière, vous pouvez le faire facilement chez vous. Il est couvert par l'assurance maladie de base sans franchise.

Quelle est l'autre option de dépistage ?

L'autre option de dépistage est une coloscopie. Un médecin (gastroentérologue) explore votre côlon entier à l'aide d'un tube flexible muni d'une caméra. C'est un test très sûr, mais qui pourrait provoquer des effets secondaires. Nous le recommandons uniquement dans le cas de test FIT positif.

À quoi devez-vous faire attention ?

Le risque du cancer du côlon augmente avec l'âge. Nous vous recommandons de discuter régulièrement de votre niveau de risque avec votre médecin. Il est toutefois possible de réduire le risque de développer le cancer du côlon grâce à un mode vie sain :

- **Manger quotidiennement des fruits et légumes**
- **Limiter la consommation de viande rouge**
- **Avoir une activité physique suffisante et régulière**
- **Contrôler votre poids**
- **Éviter le tabac et l'excès d'alcool**

12479_UNISANTE_Depliant_Colon_risquefaible.indd 2
04.05.22 10:22

Comment faire un test FIT chez soi ?

1 **Allez dans une pharmacie:**
Un pharmacien vous explique le test.
Le pharmacien vous donne le test et son mode d'emploi.

Ou

Prenez rendez-vous chez votre médecin de famille:
Votre médecin vous donne une ordonnance.
Vous allez ensuite chercher le test et son mode d'emploi dans une pharmacie.

---

2 **Faire mon test FIT à domicile**

— Vous collectez d'abord les selles sur un papier spécial à coller à la lunette des toilettes.
— Vous grattez ensuite les selles plusieurs fois avec la tige du tube de collecte.
— Vous envoyez le tube par la poste au laboratoire.

---

3 **Vous et votre médecin recevrez le résultat du test par courrier en moins d'une semaine.**

Le test est négatif Le test n'a rien montré. Cela veut dire qu'il n'y a pas de signe de cancer dans les selles. Le test doit être refait dans 2 ans.

▶ Si vous avez des symptômes entre-temps, consultez votre médecin sans tarder.

Le test est positif Le test montre du sang dans les selles. Ceci ne veut pas forcément dire que vous avez un cancer. Mais il faut rechercher la cause du sang dans les selles.

▶ Prenez rendez-vous chez votre médecin de famille pour organiser une coloscopie.

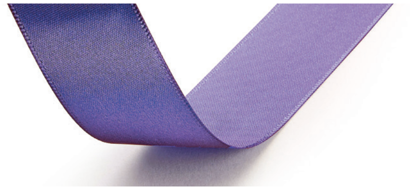

Quand devrez-vous consulter un médecin ?

Il n'existe pas de test qui détecte à 100% tous les cancers à un stade précoce.
Si vous avez un des symptômes listés ci-dessous, prenez un rendez-vous avec votre médecin de famille:

- Sang dans les selles,
- Troubles digestifs,
- Douleurs dans le ventre, diarrhée ou constipation,
- Selles plus ou moins fréquentes que d'habitude,
- Perte de poids inexpliquée,
- Fatigue durable.

Comment le dépistage est-il pris en charge ?

Les deux tests sont pris en charge par les caisses maladie pour les personnes entre 50 et 69 ans habitant dans le canton de Vaud.
Vous ne payerez pas de franchise. Il vous restera à payer la quotepart de 10%, c'est-à-dire environ CHF 5.– pour le test FIT.
Si vous avez plus de 69 ans, demandez à votre médecin pour savoir si le dépistage est toujours adapté pour vous.

Pour plus d'informations
Programmes vaudois de dépistage du cancer: www.pvdc.ch
Swiss Cancer Screening: www.swisscancerscreening.ch
Votre médecin de famille ou votre pharmacien.

v3.0_avril_2022

unisanté
Centre universitaire de médecine générale et santé publique • Lausanne

Unisanté
Route de Berne 113, 1010 Lausanne
Tel: 0848 990 990, Fax: 021 314 14 46
depistage.colon@unisante.ch, wwwpvdc.ch

Dépistage du cancer du côlon
Canton de Vaud

DÉPISTAGE DU CANCER DU CÔLON

À partir de 50 ans je m'informe

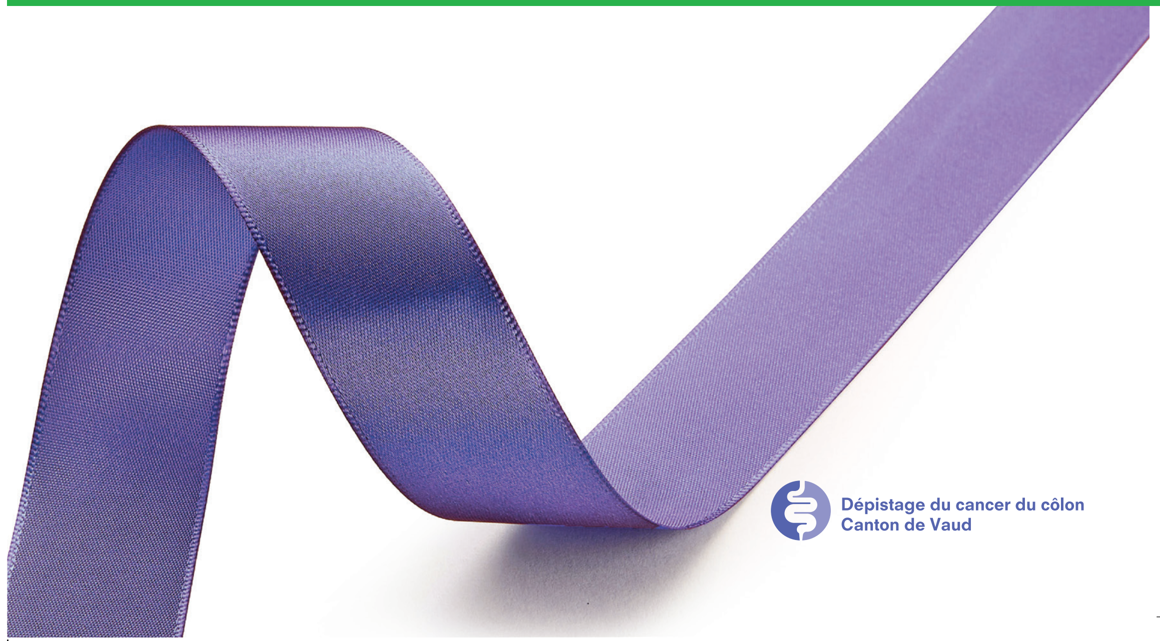

Dépistage du cancer du côlon
Canton de Vaud

12479_UNISANTE_Depliant_Colon_risquefaible.indd 1 04.05.22 10:22

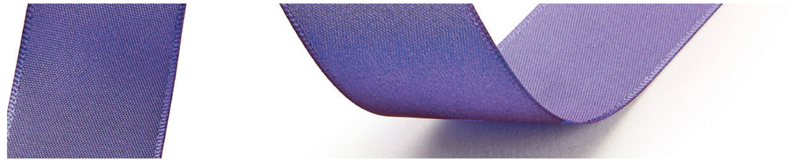

Que dois-je savoir sur le cancer du côlon ?

La plupart des cancers du côlon apparaissent après l'âge de 50 ans. De petites grosseurs (bosses) peuvent apparaître dans le côlon : on les appelle polypes. La plupart de ceux-ci sont sans danger, mais un faible nombre d'entre eux peut lentement évoluer en cancer.

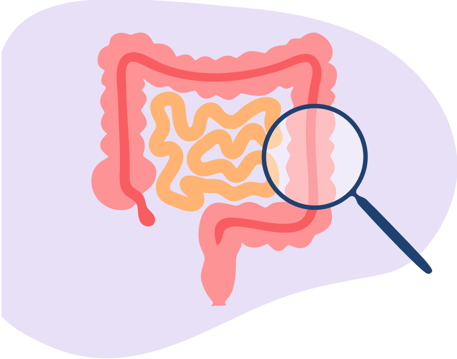

Quels sont les effets positifs du dépistage ?

- Découverte de cancers débutants : traitements allégés avec de meilleures chances de guérison.
- Moins de risque d'avoir un cancer du côlon.

Mon risque de développer un cancer du côlon

Actuellement, le risque d'avoir un cancer du côlon peut être calculé pour chaque personne.
Pour calculer votre niveau de risque, nous avons utilisé les réponses au questionnaire que vous avez rempli. Cela permet de faire une recommandation sur la méthode de dépistage qui est adaptée pour vous.

Selon nos calculs, vous êtes à faible risque

Nous vous recommandons un test FIT

D'après nos estimations 2 personnes sur 100 ayant le même profil que vous, auront un cancer du côlon dans les 15 prochaines années.

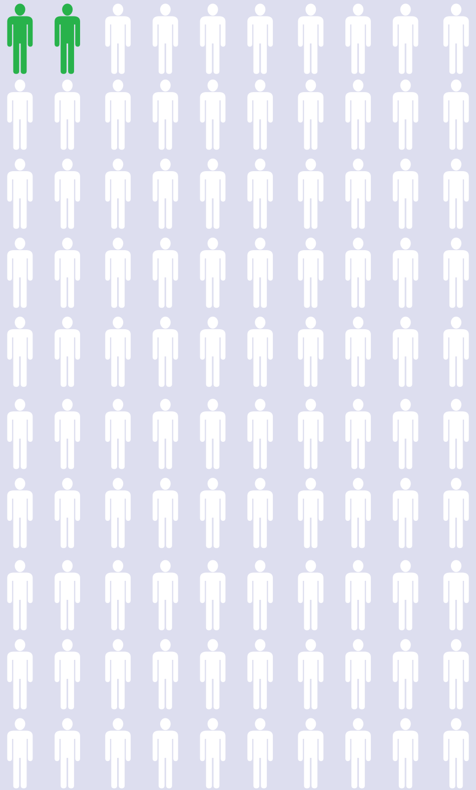

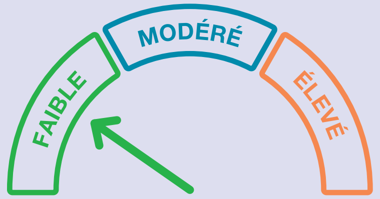

Devez-vous vous faire dépister ?

Oui, le dépistage est recommandé pour les personnes à faible risque. Le calculateur du risque n'est pas parfait. Bien que votre risque soit faible, il n'est pas nul. Le test FIT peut trouver des cancers débutants et des polypes qui saignent.

90 personnes sur 100 guérissent si le cancer est trouvé tôt.

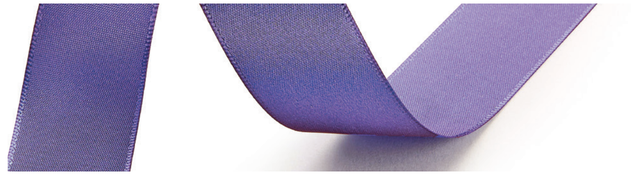

Pourquoi le test FIT est adapté pour vous ?

Le test adapté pour les personnes à faible risque est le test FIT qui sert à chercher du sang invisible à l'œil nu dans les selles. Ce test se fait tous les deux ans et permet de trouver la grande majorité de cancers débutants.
Ce test ne nécessite aucune préparation particulière, vous pouvez le faire facilement chez vous. Il est couvert par l'assurance maladie de base sans franchise.

Quelle est l'autre option de dépistage ?

L'autre option de dépistage est une coloscopie. Un médecin (gastroentérologue) explore votre côlon entier à l'aide d'un tube flexible muni d'une caméra. C'est un test très sûr, mais qui pourrait provoquer des effets secondaires. Nous le recommandons uniquement dans le cas de test FIT positif.

À quoi devez-vous faire attention ?

Le risque du cancer du côlon augmente avec l'âge. Nous vous recommandons de discuter régulièrement de votre niveau de risque avec votre médecin. Il est toutefois possible de réduire le risque de développer le cancer du côlon grâce à un mode vie sain :

- **Manger quotidiennement des fruits et légumes**
- **Limiter la consommation de viande rouge**
- **Avoir une activité physique suffisante et régulière**
- **Contrôler votre poids**
- **Éviter le tabac et l'excès d'alcool**

12479_UNISANTE_Depliant_Colon_risquefaible.indd 3 04.05.22 10:22

Vous décidez de faire :

Un test FIT

1

Allez dans une pharmacie :
Un pharmacien vous explique le test. Le pharmacien vous donne le test et son mode d'emploi.
ou

Prenez rendez-vous chez votre médecin de famille :
Votre médecin vous donne une ordonnance. Vous allez ensuite chercher le test et son mode d'emploi dans une pharmacie.

2

Faire mon test FIT à domicile
— Vous collectez d'abord les selles sur un papier spécial à coller à la lunette des toilettes.
— Vous grattez ensuite les selles plusieurs fois avec la tige du tube de collecte.
— Vous envoyez le tube par la poste au laboratoire.

3

Vous et votre médecin recevrez le résultat du test par courrier en moins d'une semaine.

Une coloscopie

1

Prenez rendez-vous chez votre médecin de famille.
Il ou elle vous conseillera et vous prescrira une coloscopie.

2 AGENDA

On vous donnera ensuite un rendez-vous chez un gastroentérologue.

3

Avant l'examen, préparez soigneusement votre intestin.
C'est une étape peu agréable mais très importante.
— Suivez le régime recommandé.
— Buvez le liquide recommandé (purge) afin de nettoyer l'intestin (généralement le soir avant l'examen et le jour de l'examen).

4

Le gastroentérologue fera l'examen et vous donnera un résultat de vive voix le jour même.

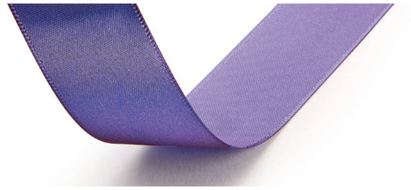

Quand devrez-vous consulter un médecin ?

Il n'existe pas de test qui détecte à 100% tous les cancers à un stade précoce.
Si vous avez un des symptômes listés ci-dessous, prenez un rendez-vous avec votre médecin de famille :

- Sang dans les selles,
- Troubles digestifs,
- Douleurs dans le ventre, diarrhée ou constipation,
- Selles plus ou moins fréquentes que d'habitude,
- Perte de poids inexpliquée,
- Fatigue durable.

Comment le dépistage est-il pris en charge ?

Les deux tests sont pris en charge par les caisses maladie pour les personnes entre 50 et 69 ans habitant dans le canton de Vaud.
Vous ne payerez pas de franchise. Il vous restera à payer la quotepart de 10%, c'est-à-dire environ CHF 5.– pour le test FIT et entre CHF 80.– et CHF 160.– pour la coloscopie.
Si vous avez plus de 69 ans, demandez à votre médecin pour savoir si le dépistage est toujours adapté pour vous.

Pour plus d'informations

Programmes vaudois de dépistage du cancer : www.pvdc.ch
Swiss Cancer Screening : www.swisscancerscreening.ch
Votre médecin de famille ou votre pharmacien.

v3.0_avril_2022

unisanté
Centre universitaire de médecine générale et santé publique • Lausanne

Unisanté
Route de Berne 113, 1010 Lausanne
Tel: 0848 990 990, Fax: 021 314 14 46
depistage.colon@unisante.ch, wwwpvdc.ch

Dépistage du cancer du côlon
Canton de Vaud

DÉPISTAGE DU CANCER DU CÔLON

À partir de 50 ans je m'informe

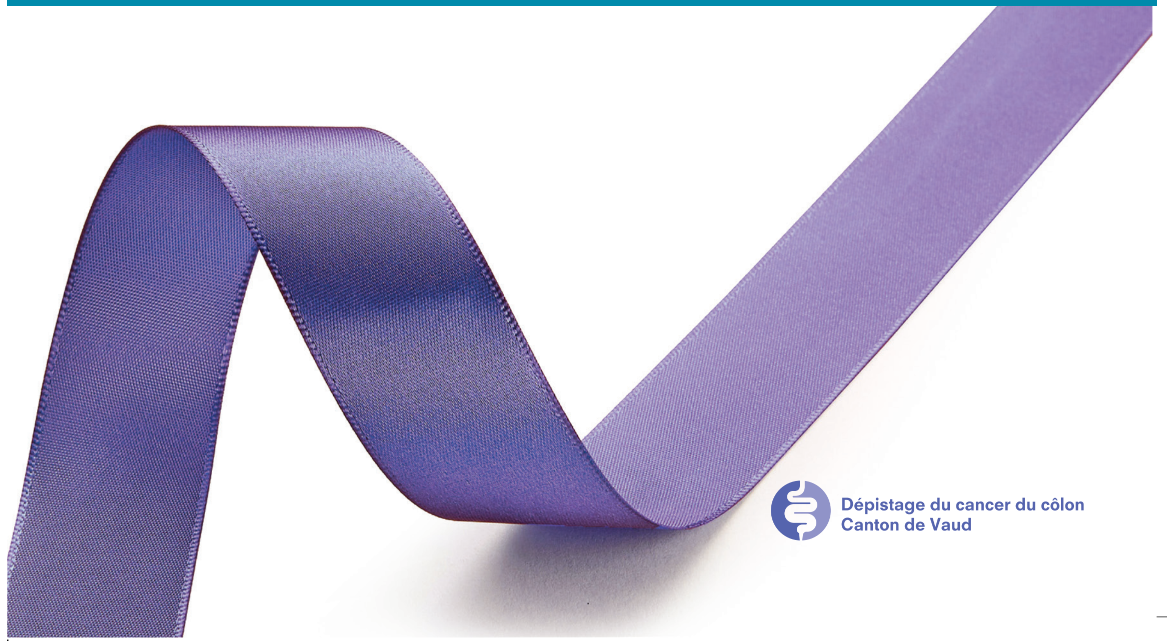

Dépistage du cancer du côlon
Canton de Vaud

12479_UNISANTE_Depliant_Colon_risquemodere.indd 1
04.05.22 10:24

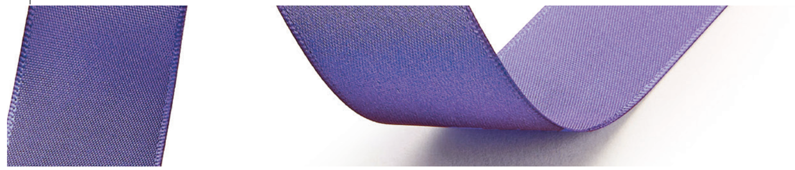

Que dois-je savoir sur le cancer du côlon ?

La plupart des cancers du côlon apparaissent après l'âge de 50 ans. De petites grosseurs (bosses) peuvent apparaître dans le côlon : on les appelle polypes. La plupart de ceux-ci sont sans danger, mais un faible nombre d'entre eux peut lentement évoluer en cancer.

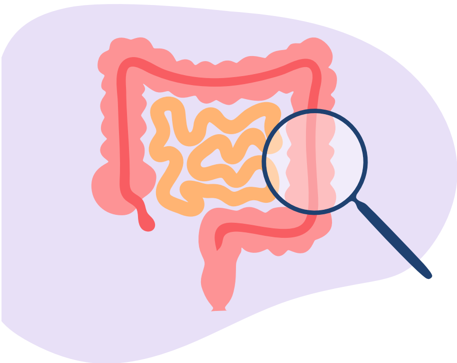

Quels sont les effets positifs du dépistage ?

- Découverte de cancers débutants : traitements allégés avec de meilleures chances de guérison.
- Moins de risque d'avoir un cancer du côlon.

Mon risque de développer un cancer du côlon

Actuellement, le risque d'avoir un cancer du côlon peut être calculé pour chaque personne.
Pour calculer votre niveau de risque, nous avons utilisé les réponses au questionnaire que vous avez rempli. Cela permet de faire une recommandation sur la méthode de dépistage qui est adaptée pour vous.

Selon nos calculs, vous êtes à risque modéré

Nous vous recommandons un test FIT ou une coloscopie

D'après nos estimations 3 personnes sur 100 ayant le même profil que vous, auront un cancer du côlon dans les 15 prochaines années.

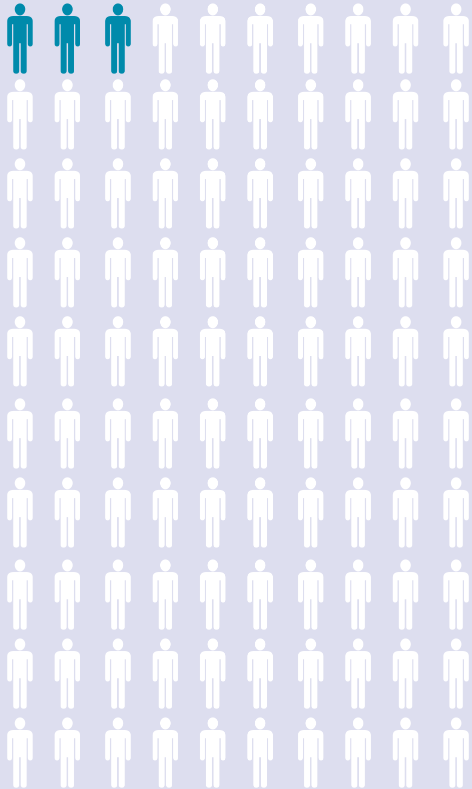

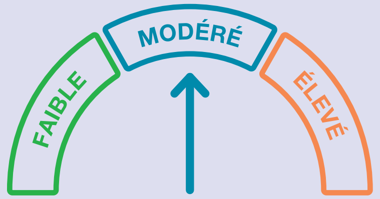

Comment diminuer votre risque d'avoir un cancer du côlon ?

Prenez un rendez-vous avec votre médecin pour en parler et faites-vous régulièrement dépister.

90 personnes sur 100 guérissent si le cancer est trouvé tôt.

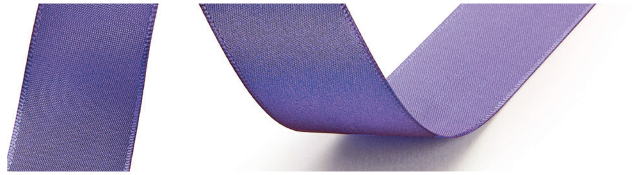

2 options sont adaptées pour vous

Test FIT
Pour chercher le sang invisible à l'œil nu dans les selles.
- Se fait facilement et rapidement à la maison.
- Pas de préparation nécessaire.
- Si le résultat est positif, il faudra faire une coloscopie.
- L'attente de la coloscopie peut être stressante.

Analyse à refaire tous les 2 ans.

Coloscopie
Pour examiner votre intestin avec un tube en passant par l'anus.
- C'est la méthode de référence pour trouver les polypes.
- Le médecin peut enlever les polypes pendant l'examen.
- Une préparation de l'intestin est nécessaire avant l'examen.
- Environ 2 personnes sur 1000 auront une perforation de l'intestin ou hémorragie importante.
- Si on vous donne un médicament sédatif (calmant), vous ne pouvez pas conduire.

Examen à refaire tous les 10 ans.

À quoi devez-vous faire attention ?

Le risque du cancer du côlon augmente avec l'âge. Nous vous recommandons de discuter régulièrement de votre niveau de risque avec votre médecin. Il est toutefois possible de réduire le risque de développer le cancer du côlon grâce à un mode vie sain :

- **Manger quotidiennement des fruits et légumes**
- **Limiter la consommation de viande rouge**
- **Avoir une activité physique suffisante et régulière**
- **Contrôler votre poids**
- **Éviter le tabac et l'excès d'alcool**

Vous décidez de faire :

Un test FIT

1

Allez dans une pharmacie :
Un pharmacien vous explique le test. Le pharmacien vous donne le test et son mode d'emploi.
ou

Prenez rendez-vous chez votre médecin de famille :
Votre médecin vous donne une ordonnance. Vous allez ensuite chercher le test et son mode d'emploi dans une pharmacie.

2

Faire mon test FIT à domicile
— Vous collectez d'abord les selles sur un papier spécial à coller à la lunette des toilettes.
— Vous grattez ensuite les selles plusieurs fois avec la tige du tube de collecte.
— Vous envoyez le tube par la poste au laboratoire.

3

Vous et votre médecin recevrez le résultat du test par courrier en moins d'une semaine.

Une coloscopie

1

Prenez rendez-vous chez votre médecin de famille.
Il ou elle vous conseillera et vous prescrira une coloscopie.

2

AGENDA

On vous donnera ensuite un rendez-vous chez un gastroentérologue.

3

Avant l'examen, préparez soigneusement votre intestin.
C'est une étape peu agréable mais très importante.
— Suivez le régime recommandé.
— Buvez le liquide recommandé (purge) afin de nettoyer l'intestin (généralement le soir avant l'examen et le jour de l'examen).

4

Le gastroentérologue fera l'examen et vous donnera un résultat de vive voix le jour même.

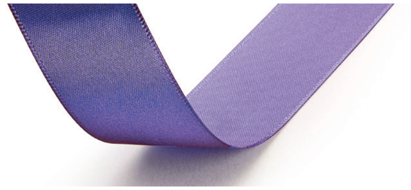

Quand devrez-vous consulter un médecin ?

Il n'existe pas de test qui détecte à 100% tous les cancers à un stade précoce.
Si vous avez un des symptômes listés ci-dessous, prenez un rendez-vous avec votre médecin de famille :

- Sang dans les selles,
- Troubles digestifs,
- Douleurs dans le ventre, diarrhée ou constipation,
- Selles plus ou moins fréquentes que d'habitude,
- Perte de poids inexpliquée,
- Fatigue durable.

Comment le dépistage est-il pris en charge ?

Les deux tests sont pris en charge par les caisses maladie pour les personnes entre 50 et 69 ans habitant dans le canton de Vaud.
Vous ne payerez pas de franchise. Il vous restera à payer la quotepart de 10%, c'est-à-dire environ CHF 5.– pour le test FIT et entre CHF 80.– et CHF 160.– pour la coloscopie.
Si vous avez plus de 69 ans, demandez à votre médecin pour savoir si le dépistage est toujours adapté pour vous.

Pour plus d'informations

Programmes vaudois de dépistage du cancer : www.pvdc.ch
Swiss Cancer Screening : www.swisscancerscreening.ch
Votre médecin de famille ou votre pharmacien.

v3.0_avril_2022

unisanté
Centre universitaire de médecine générale et santé publique • Lausanne

Unisanté
Route de Berne 113, 1010 Lausanne
Tel: 0848 990 990, Fax: 021 314 14 46
depistage.colon@unisante.ch, wwwpvdc.ch

Dépistage du cancer du côlon
Canton de Vaud

DÉPISTAGE DU CANCER DU CÔLON

À partir de 50 ans je m'informe

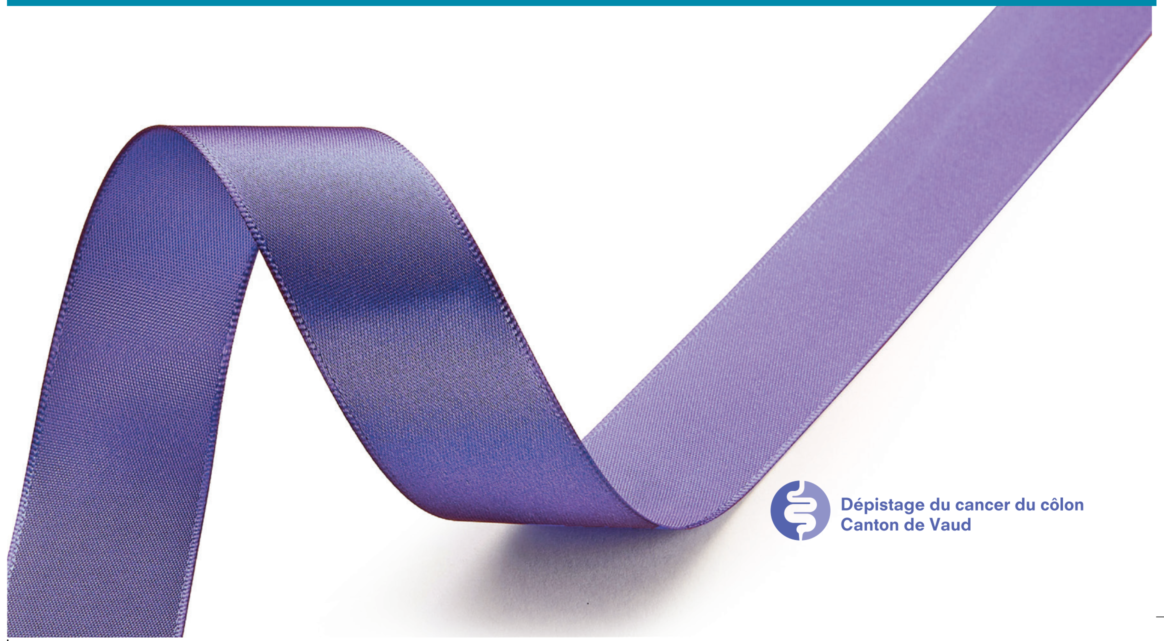

Dépistage du cancer du côlon
Canton de Vaud

12479_UNISANTE_Depliant_Colon_risquemodere.indd 1
04.05.22 10:24

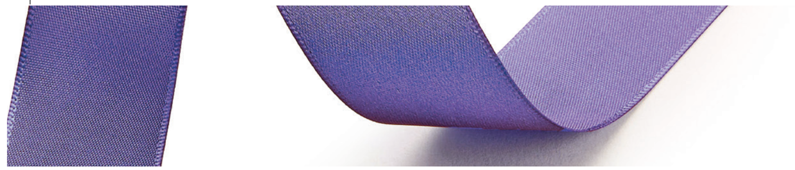

Que dois-je savoir sur le cancer du côlon ?

La plupart des cancers du côlon apparaissent après l'âge de 50 ans. De petites grosseurs (bosses) peuvent apparaître dans le côlon : on les appelle polypes. La plupart de ceux-ci sont sans danger, mais un faible nombre d'entre eux peut lentement évoluer en cancer.

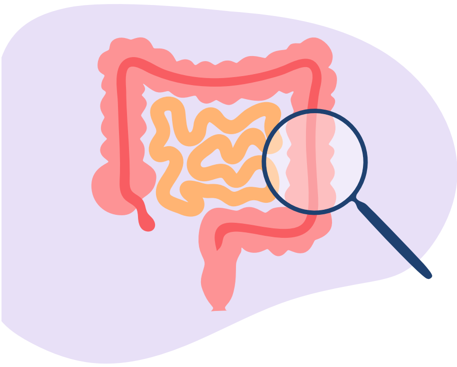

Quels sont les effets positifs du dépistage ?

- Découverte de cancers débutants : traitements allégés avec de meilleures chances de guérison.
- Moins de risque d'avoir un cancer du côlon.

Mon risque de développer un cancer du côlon

Actuellement, le risque d'avoir un cancer du côlon peut être calculé pour chaque personne.
Pour calculer votre niveau de risque, nous avons utilisé les réponses au questionnaire que vous avez rempli. Cela permet de faire une recommandation sur la méthode de dépistage qui est adaptée pour vous.

Selon nos calculs, vous êtes à risque modéré

Nous vous recommandons un test FIT ou une coloscopie

D'après nos estimations 4 personnes sur 100 ayant le même profil que vous, auront un cancer du côlon dans les 15 prochaines années.

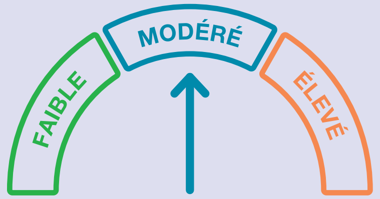

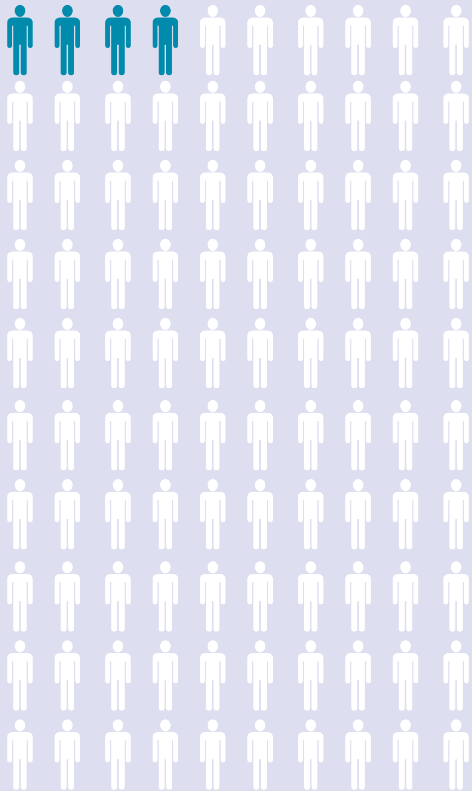

Comment diminuer votre risque d'avoir un cancer du côlon ?

Prenez un rendez-vous avec votre médecin pour en parler et faites-vous régulièrement dépister.

90 personnes sur 100 guérissent si le cancer est trouvé tôt.

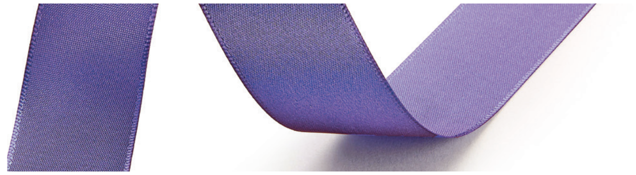

2 options sont adaptées pour vous

Test FIT
Pour chercher le sang invisible à l'œil nu dans les selles.
— Se fait facilement et rapidement à la maison.
— Pas de préparation nécessaire.
— Si le résultat est positif, il faudra faire une coloscopie.
— L'attente de la coloscopie peut être stressante.
Analyse à refaire tous les 2 ans.

Coloscopie
Pour examiner votre intestin avec un tube en passant par l'anus.
— C'est la méthode de référence pour trouver les polypes.
— Le médecin peut enlever les polypes pendant l'examen.
— Une préparation de l'intestin est nécessaire avant l'examen.
— Environ 2 personnes sur 1000 auront une perforation de l'intestin ou hémorragie importante.
— Si on vous donne un médicament sédatif (calmant), vous ne pouvez pas conduire.
Examen à refaire tous les 10 ans.

À quoi devez-vous faire attention ?

Le risque du cancer du côlon augmente avec l'âge. Nous vous recommandons de discuter régulièrement de votre niveau de risque avec votre médecin. Il est toutefois possible de réduire le risque de développer le cancer du côlon grâce à un mode vie sain :

- **Manger quotidiennement des fruits et légumes**
- **Limiter la consommation de viande rouge**
- **Avoir une activité physique suffisante et régulière**
- **Contrôler votre poids**
- **Éviter le tabac et l'excès d'alcool**

12479_UNISANTE_Depliant_Colon_risquemodere.indd 3
04.05.22 10:25

Vous décidez de faire :

Un test FIT

1

Allez dans une pharmacie :
Un pharmacien vous explique le test. Le pharmacien vous donne le test et son mode d'emploi.
ou

Prenez rendez-vous chez votre médecin de famille :
Votre médecin vous donne une ordonnance. Vous allez ensuite chercher le test et son mode d'emploi dans une pharmacie.

2

Faire mon test FIT à domicile
— Vous collectez d'abord les selles sur un papier spécial à coller à la lunette des toilettes.
— Vous grattez ensuite les selles plusieurs fois avec la tige du tube de collecte.
— Vous envoyez le tube par la poste au laboratoire.

3

Vous et votre médecin recevrez le résultat du test par courrier en moins d'une semaine.

Une coloscopie

1

Prenez rendez-vous chez votre médecin de famille.
Il ou elle vous conseillera et vous prescrira une coloscopie.

2

AGENDA

On vous donnera ensuite un rendez-vous chez un gastroentérologue.

3

Avant l'examen, préparez soigneusement votre intestin.
C'est une étape peu agréable mais très importante.
— Suivez le régime recommandé.
— Buvez le liquide recommandé (purge) afin de nettoyer l'intestin (généralement le soir avant l'examen et le jour de l'examen).

4

Le gastroentérologue fera l'examen et vous donnera un résultat de vive voix le jour même.

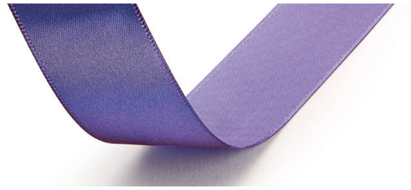

Quand devrez-vous consulter un médecin ?

Il n'existe pas de test qui détecte à 100% tous les cancers à un stade précoce.
Si vous avez un des symptômes listés ci-dessous, prenez un rendez-vous avec votre médecin de famille :

- Sang dans les selles,
- Troubles digestifs,
- Douleurs dans le ventre, diarrhée ou constipation,
- Selles plus ou moins fréquentes que d'habitude,
- Perte de poids inexpliquée,
- Fatigue durable.

Comment le dépistage est-il pris en charge ?

Les deux tests sont pris en charge par les caisses maladie pour les personnes entre 50 et 69 ans habitant dans le canton de Vaud.
Vous ne payerez pas de franchise. Il vous restera à payer la quotepart de 10%, c'est-à-dire environ CHF 5.– pour le test FIT et entre CHF 80.– et CHF 160.– pour la coloscopie.
Si vous avez plus de 69 ans, demandez à votre médecin pour savoir si le dépistage est toujours adapté pour vous.

Pour plus d'informations

Programmes vaudois de dépistage du cancer : www.pvdc.ch
Swiss Cancer Screening : www.swisscancerscreening.ch
Votre médecin de famille ou votre pharmacien.

v3.0_avril_2022

unisanté
Centre universitaire de médecine générale et santé publique • Lausanne

Unisanté
Route de Berne 113, 1010 Lausanne
Tel: 0848 990 990, Fax: 021 314 14 46
depistage.colon@unisante.ch, wwwpvdc.ch

Dépistage du cancer du côlon
Canton de Vaud

DÉPISTAGE DU CANCER DU CÔLON

À partir de 50 ans je m'informe

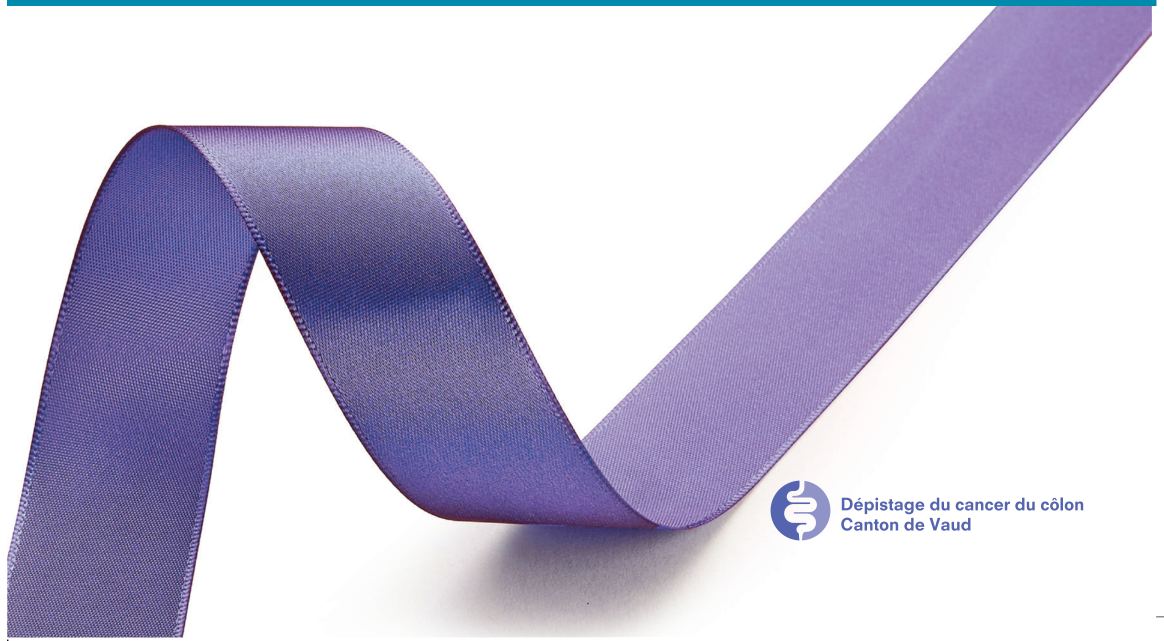

Dépistage du cancer du côlon
Canton de Vaud

12479_UNISANTE_Depliant_Colon_risquemodere.indd 1 04.05.22 10:25

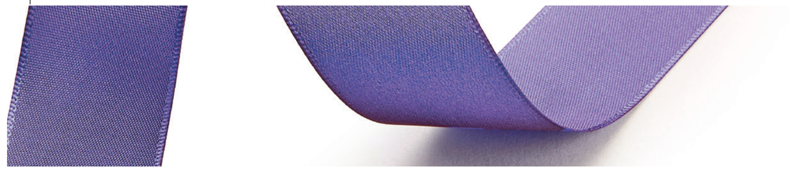

Que dois-je savoir sur le cancer du côlon ?

La plupart des cancers du côlon apparaissent après l'âge de 50 ans. De petites grosseurs (bosses) peuvent apparaître dans le côlon : on les appelle polypes. La plupart de ceux-ci sont sans danger, mais un faible nombre d'entre eux peut lentement évoluer en cancer.

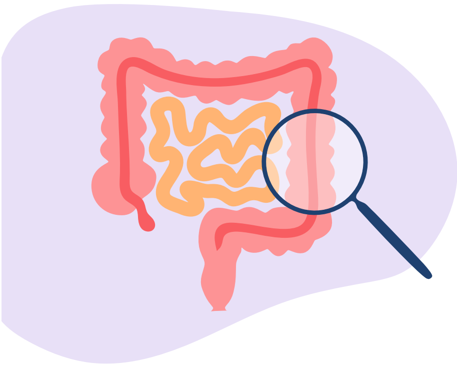

Quels sont les effets positifs du dépistage ?

- Découverte de cancers débutants : traitements allégés avec de meilleures chances de guérison.
- Moins de risque d'avoir un cancer du côlon.

Mon risque de développer un cancer du côlon

Actuellement, le risque d'avoir un cancer du côlon peut être calculé pour chaque personne.
Pour calculer votre niveau de risque, nous avons utilisé les réponses au questionnaire que vous avez rempli. Cela permet de faire une recommandation sur la méthode de dépistage qui est adaptée pour vous.

Selon nos calculs, vous êtes à risque modéré

Nous vous recommandons un test FIT ou une coloscopie

D'après nos estimations 5 personnes sur 100 ayant le même profil que vous, auront un cancer du côlon dans les 15 prochaines années.

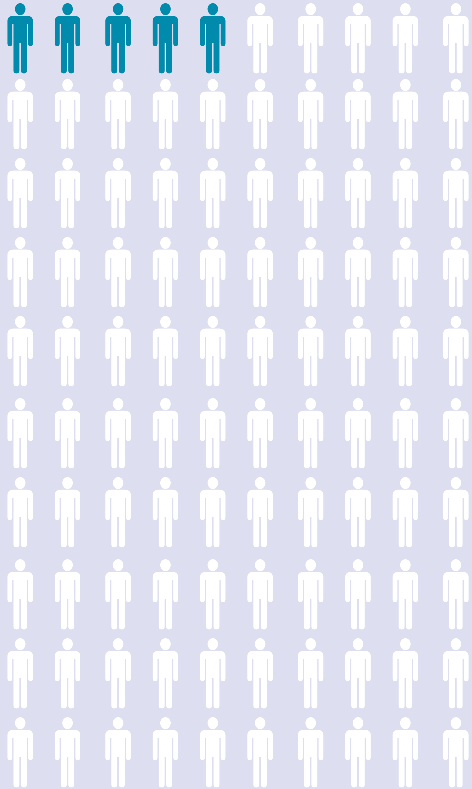

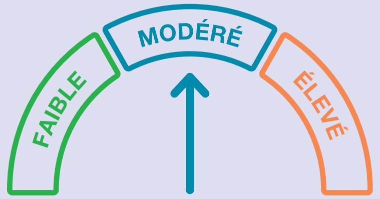

Comment diminuer votre risque d'avoir un cancer du côlon ?

Prenez un rendez-vous avec votre médecin pour en parler et faites-vous régulièrement dépister.

90 personnes sur 100 guérissent si le cancer est trouvé tôt.

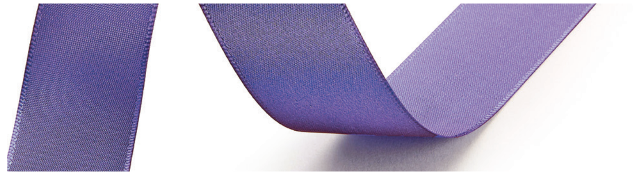

2 options sont adaptées pour vous

Test FIT

Pour chercher le sang invisible à l'œil nu dans les selles.
- Se fait facilement et rapidement à la maison.
- Pas de préparation nécessaire.
- Si le résultat est positif, il faudra faire une coloscopie.
- L'attente de la coloscopie peut être stressante.

Analyse à refaire tous les 2 ans.

Coloscopie

Pour examiner votre intestin avec un tube en passant par l'anus.
- C'est la méthode de référence pour trouver les polypes.
- Le médecin peut enlever les polypes pendant l'examen.
- Une préparation de l'intestin est nécessaire avant l'examen.
- Environ 2 personnes sur 1000 auront une perforation de l'intestin ou hémorragie importante.
- Si on vous donne un médicament sédatif (calmant), vous ne pouvez pas conduire.

Examen à refaire tous les 10 ans.

À quoi devez-vous faire attention ?

Le risque du cancer du côlon augmente avec l'âge. Nous vous recommandons de discuter régulièrement de votre niveau de risque avec votre médecin. Il est toutefois possible de réduire le risque de développer le cancer du côlon grâce à un mode vie sain :

- **Manger quotidiennement des fruits et légumes**
- **Limiter la consommation de viande rouge**
- **Avoir une activité physique suffisante et régulière**
- **Contrôler votre poids**
- **Éviter le tabac et l'excès d'alcool**

12479_UNISANTE_Depliant_Colon_risquemodere.indd 4
04.05.22 10:25

Quelles sont les démarches pour faire une coloscopie ?

1 **Prenez rendez-vous chez votre médecin de famille :**
Il ou elle vous conseillera et vous prescrira une coloscopie.

2 On vous donnera ensuite un rendez-vous chez un gastroentérologue.

3 Avant l'examen, préparez soigneusement votre intestin. C'est une étape peu agréable mais très importante.
— Suivez le régime recommandé.
— Buvez le liquide recommandé (purge) afin de nettoyer l'intestin (généralement le soir avant l'examen et le jour de l'examen).

4 **Le gastroentérologue fera l'examen et vous donnera un résultat de vive voix le jour même.**

La coloscopie n'a rien montré

— Important : la coloscopie doit être refaite 10 ans plus tard.
— Si vous avez des symptômes entre-temps, consultez votre médecin sans tarder.

La coloscopie montre des polypes

— Le médecin enlève généralement les polypes pendant la coloscopie.
— Si le médecin suspecte un cancer, il vous expliquera le jour même la marche à suivre.

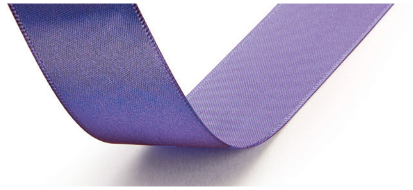

Quand devrez-vous consulter un médecin ?

Il n'existe pas de test qui détecte à 100% tous les cancers à un stade précoce.
Si vous avez un des symptômes listés ci-dessous, prenez un rendez-vous avec votre médecin de famille :

- Sang dans les selles,
- Troubles digestifs,
- Douleurs dans le ventre, diarrhée ou constipation,
- Selles plus ou moins fréquentes que d'habitude,
- Perte de poids inexpliquée,
- Fatigue durable.

Comment le dépistage est-il pris en charge ?

Les deux tests sont pris en charge par les caisses maladie pour les personnes entre 50 et 69 ans habitant dans le canton de Vaud. Vous ne payerez pas de franchise. Il vous restera à payer la quotepart de 10%, c'est-à-dire CHF 80.– à CHF 160.– pour la coloscopie.
Si vous avez plus de 69 ans, demandez à votre médecin pour savoir si le dépistage est toujours adapté pour vous.

Pour plus d'informations

Programmes vaudois de dépistage du cancer : www.pvdc.ch
Swiss Cancer Screening : www.swisscancerscreening.ch
Votre médecin de famille ou votre pharmacien.

v3.0_avril_2022

unisanté
Centre universitaire de médecine générale et santé publique · Lausanne

Unisanté
Route de Berne 113, 1010 Lausanne
Tel: 0848 990 990, Fax: 021 314 14 46
depistage.colon@unisante.ch, wwwpvdc.ch

Dépistage du cancer du côlon
Canton de Vaud

DÉPISTAGE DU CANCER DU CÔLON

À partir de 50 ans je m'informe

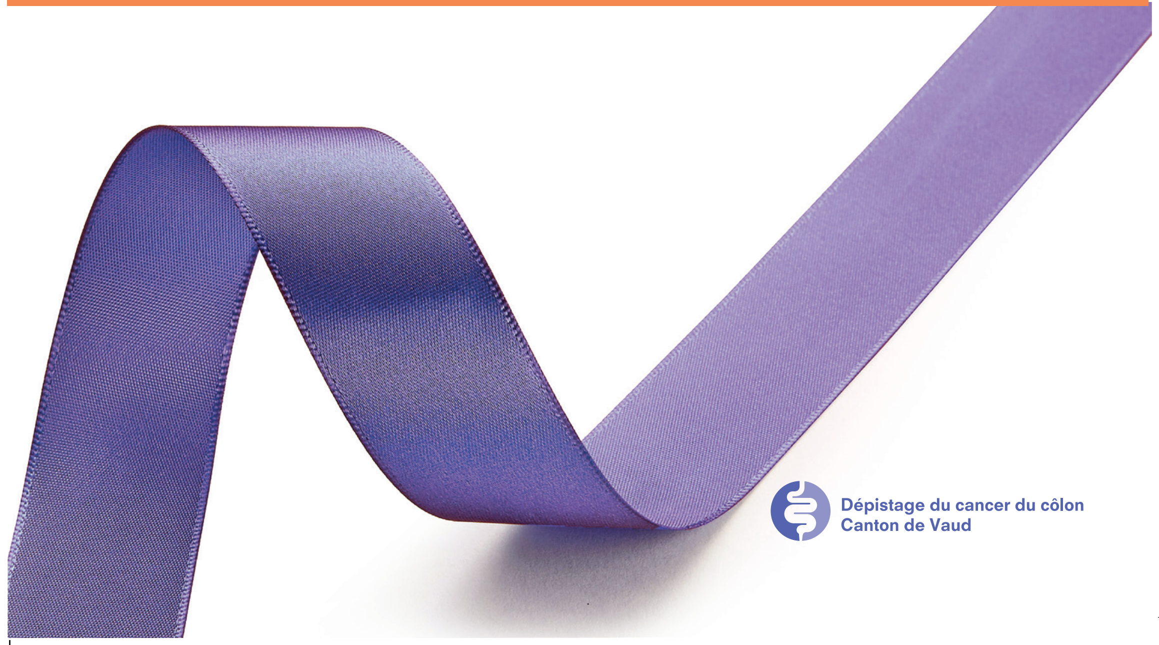

Dépistage du cancer du côlon
Canton de Vaud

12479_UNISANTE_Depliant_Colon_risqueeleve.indd 1 04.05.22 10:23

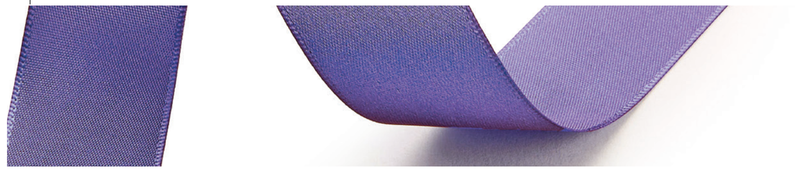

Que dois-je savoir sur le cancer du côlon ?

La plupart des cancers du côlon apparaissent après l'âge de 50 ans. De petites grosseurs (bosses) peuvent apparaître dans le côlon : on les appelle polypes. La plupart de ceux-ci sont sans danger, mais un faible nombre d'entre eux peut lentement évoluer en cancer.

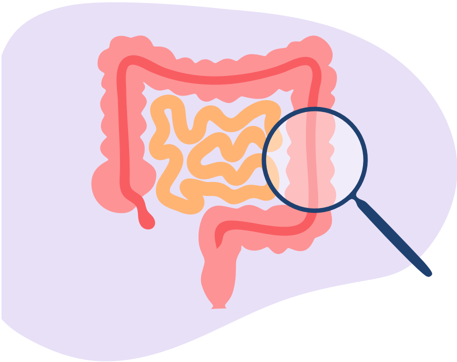

Quels sont les effets positifs du dépistage ?

— Découverte de cancers débutants : traitements allégés avec de meilleures chances de guérison.
— Moins de risque d'avoir un cancer du côlon.

Mon risque de développer un cancer du côlon

Actuellement, le risque d'avoir un cancer du côlon peut être calculé pour chaque personne.
Pour calculer votre niveau de risque, nous avons utilisé les réponses au questionnaire que vous avez rempli. Cela permet de faire une recommandation sur la méthode de dépistage qui est adaptée pour vous.

Selon nos calculs, vous êtes à risque élevé

Nous vous recommandons une coloscopie

D'après nos estimations **6 personnes sur 100** ayant le même profil que vous, auront un cancer du côlon **dans les 15 prochaines années.**

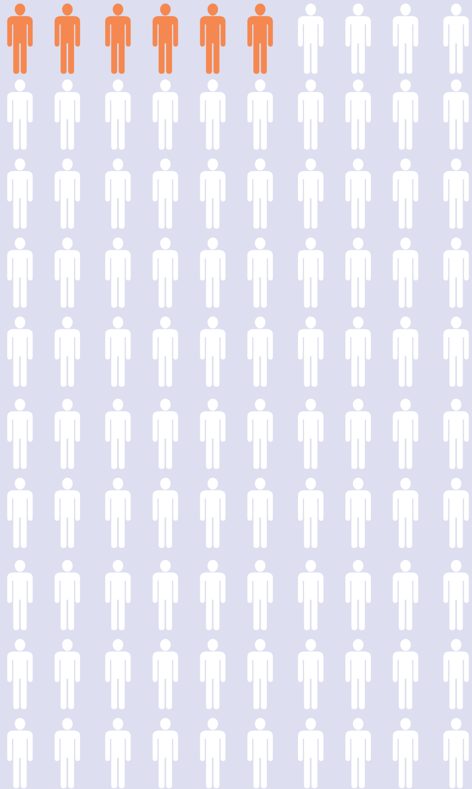

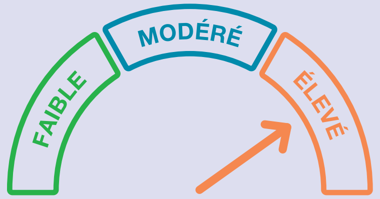

Comment diminuer votre risque d'avoir un cancer du côlon ?

Prenez un rendez-vous avec votre médecin pour en parler.
Faites-vous régulièrement dépister avec coloscopie.

90 personnes sur 100 guérissent si le cancer est trouvé tôt.

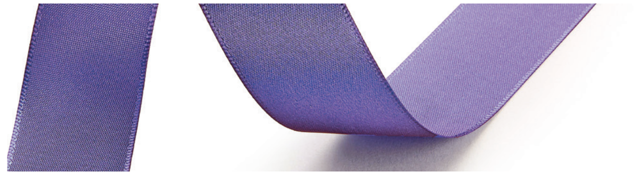

Pourquoi une coloscopie est adaptée pour vous ?

Une coloscopie est faite par un médecin gastroentérologue. Lors de l'examen, le médecin introduit un tube dans l'intestin en passant par l'anus. Une petite caméra située au bout du tube permet d'observer tout l'intestin. Si le médecin trouve des polypes, il les enlève pendant l'examen. Cela diminue fortement le risque d'avoir un cancer.
Le dépistage avec coloscopie se fait tous les 10 ans. Il est couvert par l'assurance maladie de base sans franchise.

Quelle est l'autre option de dépistage ?

L'autre option de dépistage est le test FIT qui sert à chercher du sang invisible à l'œil nu dans les selles. Ce test se fait tous les deux ans et permet de trouver la grande majorité de cancers débutants. Cependant, il ne permet pas de trouver et d'enlever les polypes. Ce test est conseillé aux personnes à faible risque.

À quoi devez-vous faire attention ?

Le risque du cancer du côlon augmente avec l'âge. Nous vous recommandons de discuter régulièrement de votre niveau de risque avec votre médecin. Il est toutefois possible de réduire le risque de développer le cancer du côlon grâce à un mode vie sain :

- **Manger quotidiennement des fruits et légumes**
- **Limiter la consommation de viande rouge**
- **Avoir une activité physique suffisante et régulière**
- **Contrôler votre poids**
- **Éviter le tabac et l'excès d'alcool**

Quelles sont les démarches pour faire une coloscopie ?

1 **Prenez rendez-vous chez votre médecin de famille :**
Il ou elle vous conseillera et vous prescrira une coloscopie.

2 On vous donnera ensuite un rendez-vous chez un gastroentérologue.

3 Avant l'examen, préparez soigneusement votre intestin. C'est une étape peu agréable mais très importante.
— Suivez le régime recommandé.
— Buvez le liquide recommandé (purge) afin de nettoyer l'intestin (généralement le soir avant l'examen et le jour de l'examen).

4 **Le gastroentérologue fera l'examen et vous donnera un résultat de vive voix le jour même.**

La coloscopie n'a rien montré

— Important : la coloscopie doit être refaite 10 ans plus tard.
— Si vous avez des symptômes entre-temps, consultez votre médecin sans tarder.

La coloscopie montre des polypes

— Le médecin enlève généralement les polypes pendant la coloscopie.
— Si le médecin suspecte un cancer, il vous expliquera le jour même la marche à suivre.

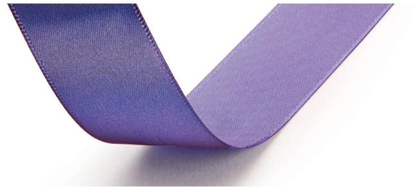

Quand devrez-vous consulter un médecin ?

Il n'existe pas de test qui détecte à 100% tous les cancers à un stade précoce.
Si vous avez un des symptômes listés ci-dessous, prenez un rendez-vous avec votre médecin de famille :

- Sang dans les selles,
- Troubles digestifs,
- Douleurs dans le ventre, diarrhée ou constipation,
- Selles plus ou moins fréquentes que d'habitude,
- Perte de poids inexpliquée,
- Fatigue durable.

Comment le dépistage est-il pris en charge ?

Les deux tests sont pris en charge par les caisses maladie pour les personnes entre 50 et 69 ans habitant dans le canton de Vaud. Vous ne payerez pas de franchise. Il vous restera à payer la quotepart de 10%, c'est-à-dire CHF 80.– à CHF 160.– pour la coloscopie.
Si vous avez plus de 69 ans, demandez à votre médecin pour savoir si le dépistage est toujours adapté pour vous.

Pour plus d'informations

Programmes vaudois de dépistage du cancer : www.pvdc.ch
Swiss Cancer Screening : www.swisscancerscreening.ch
Votre médecin de famille ou votre pharmacien.

v3.0_avril_2022

unisanté
Centre universitaire de médecine générale et santé publique • Lausanne

Unisanté
Route de Berne 113, 1010 Lausanne
Tel: 0848 990 990, Fax: 021 314 14 46
depistage.colon@unisante.ch, wwwpvdc.ch

Dépistage du cancer du côlon
Canton de Vaud

DÉPISTAGE DU CANCER DU CÔLON

À partir de 50 ans je m'informe

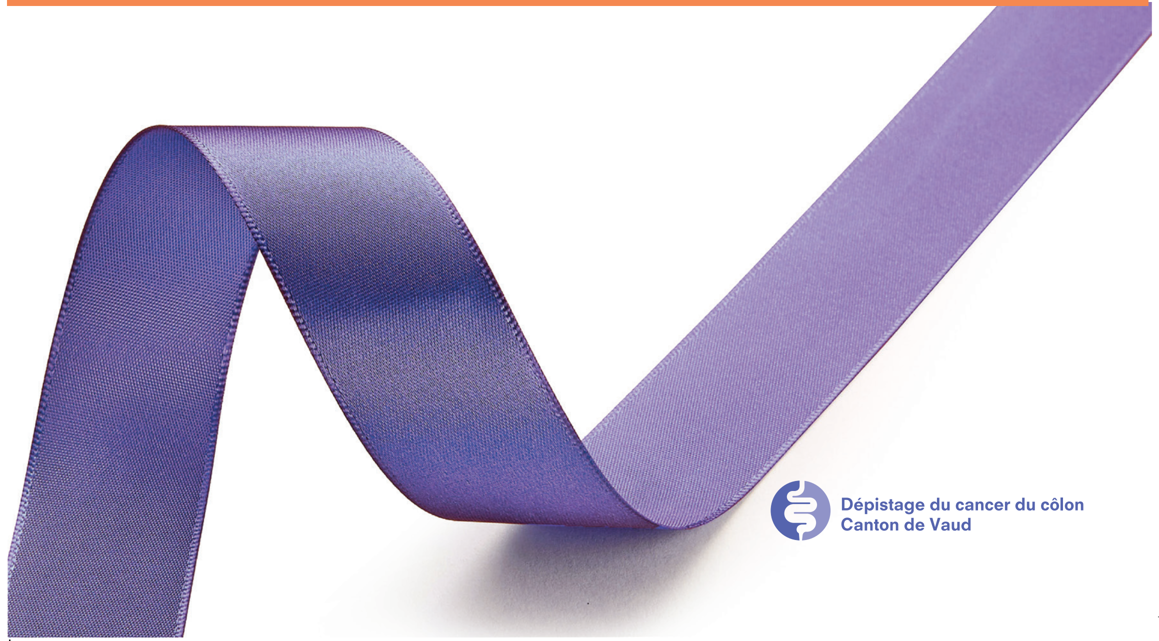

Dépistage du cancer du côlon
Canton de Vaud

12479_UNISANTE_Depliant_Colon_risqueeleve.indd 1
04.05.22 10:24

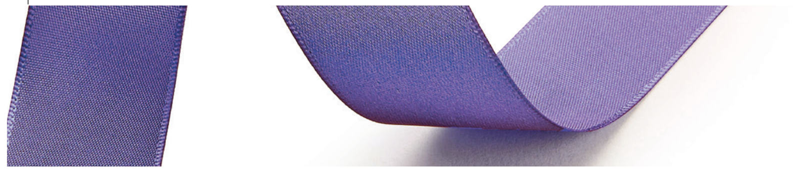

Que dois-je savoir sur le cancer du côlon ?

La plupart des cancers du côlon apparaissent après l’âge de 50 ans. De petites grosseurs (bosses) peuvent apparaître dans le côlon : on les appelle polypes. La plupart de ceux-ci sont sans danger, mais un faible nombre d’entre eux peut lentement évoluer en cancer.

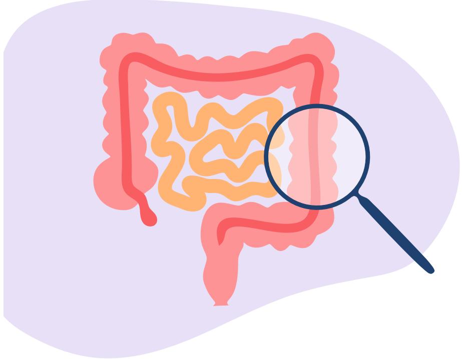

Quels sont les effets positifs du dépistage ?

- Découverte de cancers débutants : traitements allégés avec de meilleures chances de guérison.
- Moins de risque d’avoir un cancer du côlon.

Mon risque de développer un cancer du côlon

Actuellement, le risque d’avoir un cancer du côlon peut être calculé pour chaque personne.
Pour calculer votre niveau de risque, nous avons utilisé les réponses au questionnaire que vous avez rempli. Cela permet de faire une recommandation sur la méthode de dépistage qui est adaptée pour vous.

Selon nos calculs, vous êtes à risque élevé

Nous vous recommandons une coloscopie

D’après nos estimations **7 personnes sur 100** ayant le même profil que vous, auront un cancer du côlon **dans les 15 prochaines années.**

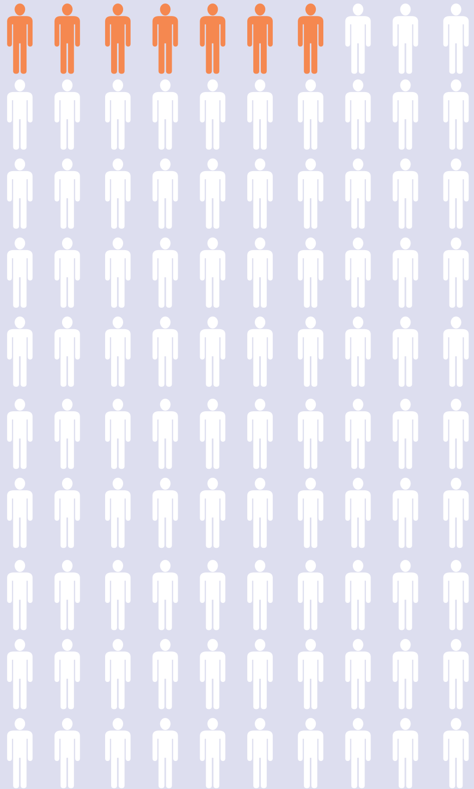

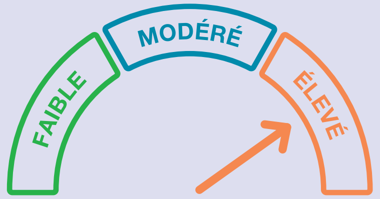

Comment diminuer votre risque d’avoir un cancer du côlon ?

Prenez un rendez-vous avec votre médecin pour en parler.
Faites-vous régulièrement dépister avec coloscopie.

90 personnes sur 100 guérissent si le cancer est trouvé tôt.

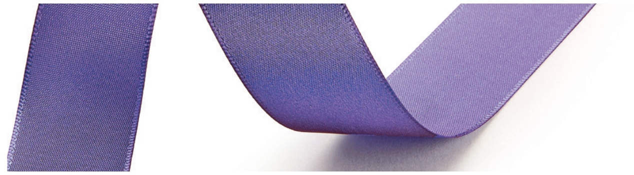

Pourquoi une coloscopie est adaptée pour vous ?

Une coloscopie est faite par un médecin gastroentérologue. Lors de l’examen, le médecin introduit un tube dans l’intestin en passant par l’anus. Une petite caméra située au bout du tube permet d’observer tout l’intestin. Si le médecin trouve des polypes, il les enlève pendant l’examen. Cela diminue fortement le risque d’avoir un cancer.
Le dépistage avec coloscopie se fait tous les 10 ans. Il est couvert par l’assurance maladie de base sans franchise.

Quelle est l’autre option de dépistage ?

L’autre option de dépistage est le test FIT qui sert à chercher du sang invisible à l’œil nu dans les selles. Ce test se fait tous les deux ans et permet de trouver la grande majorité de cancers débutants. Cependant, il ne permet pas de trouver et d’enlever les polypes. Ce test est conseillé aux personnes à faible risque.

À quoi devez-vous faire attention ?

Le risque du cancer du côlon augmente avec l’âge. Nous vous recommandons de discuter régulièrement de votre niveau de risque avec votre médecin. Il est toutefois possible de réduire le risque de développer le cancer du côlon grâce à un mode vie sain :

- **Manger quotidiennement des fruits et légumes**
- **Limiter la consommation de viande rouge**
- **Avoir une activité physique suffisante et régulière**
- **Contrôler votre poids**
- **Éviter le tabac et l’excès d’alcool**

12479_UNISANTE_Depliant_Colon_risqueeleve.indd 3 04.05.22 10:24

Le dépistage du cancer du côlon

Dépliant d'information

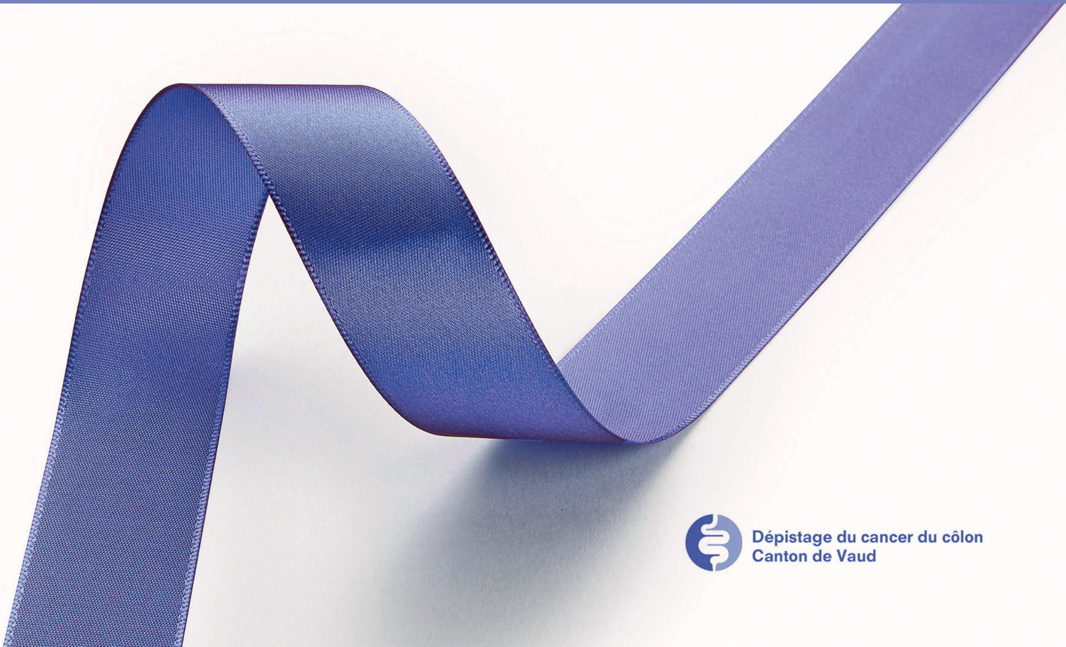

Dépistage du cancer du côlon
Canton de Vaud

Qu’est-ce que le cancer du côlon ?

La plupart des cancers du côlon apparaissent après l’âge de 50 ans.
De petites boules (bosses) peuvent apparaître dans le côlon : on les appelle polypes. La plupart des polypes sont sans danger. Un faible nombre d’entre eux peuvent lentement évoluer en cancer.

Quels sont les effets positifs du dépistage ?

- Moins de personnes auront un cancer du côlon.
- Découverte de cancers débutants : traitements moins lourds avec de meilleures chances de guérison.
- Moins de personnes mourront d’un cancer du côlon.

Nombre de personnes qui meurent du cancer du côlon avant l‘âge de 80 ans

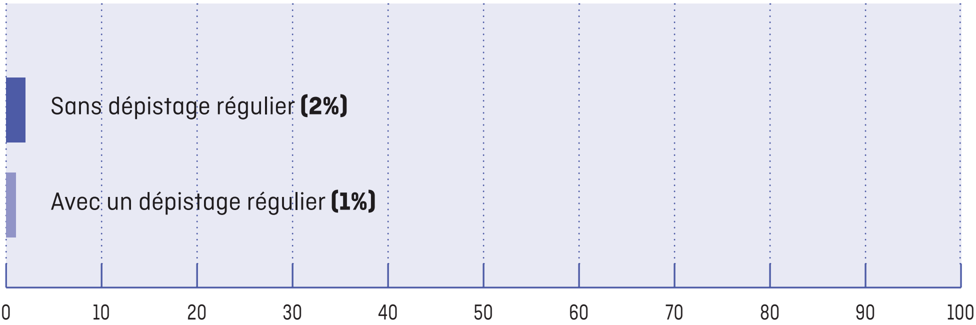

Les examens de dépistage

Il existe deux examens principaux : le test FIT (test de recherche de sang dans les selles) et la coloscopie. Vous pouvez choisir de faire le test FIT **ou** la coloscopie.

**Test FIT**<br>**Test de recherche de sang dans les selles**	**Coloscopie**<br>**Examen endoscopique du côlon**
Dès le début, le cancer du côlon cause généralement de petits saignements dans les selles. Le sang n’est pas visible à l’œil nu. Le test FIT permet de détecter ces traces de sang dans les selles.	Un médecin spécialisé (gastroentérologue) fait la coloscopie. Lors de l’examen, le médecin introduit un tube dans l’intestin en passant par l’anus. Une petite caméra au bout du tube permet d’observer tout le gros intestin.

Vous décidez de faire le test FIT

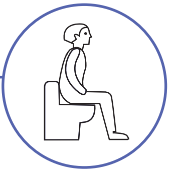

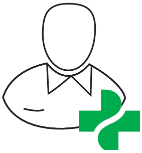

Allez dans une pharmacie :

- Un pharmacien vous explique le test.
- Le pharmacien vous donne le test et son mode d'emploi.

OU

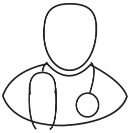

Prenez rendez-vous chez votre médecin de famille :

- Votre médecin vous donne une ordonnance.
- Vous allez ensuite chercher le test et son mode d'emploi dans une pharmacie.

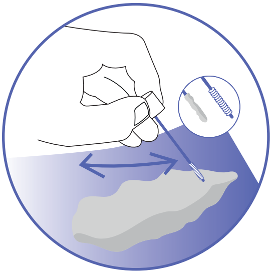

Comment faire le test FIT à mon domicile ?

- Vous collectez d'abord les selles sur un papier spécial, à coller à la lunette des toilettes.
- Vous grattez ensuite les selles plusieurs fois avec la tige du tube de collecte.
- Vous envoyez le tube par la poste au laboratoire.

Vous et votre médecin recevrez le résultat du test par courrier en moins d'une semaine.

Le test n'a rien montré :
Le test est négatif. Cela veut dire qu'il n'y a pas de signe de cancer dans les selles.

- Le test doit être refait dans 2 ans.
- Si vous avez des symptômes entre-temps, consultez votre médecin sans tarder.

Le test montre du sang dans les selles :
Le test est positif. Ceci ne veut pas forcément dire que vous avez un cancer. Mais il faut rechercher la cause du sang dans les selles.

- Prenez rendez-vous chez votre médecin de famille pour organiser une coloscopie.

Vous décidez de faire la coloscopie

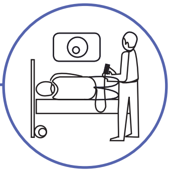

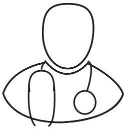

Prenez rendez-vous chez votre médecin de famille.
Il ou elle vous conseillera et vous prescrira une coloscopie.

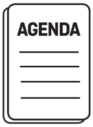

On vous donnera ensuite un rendez-vous de coloscopie chez un gastroentérologue.

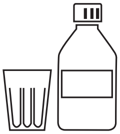

Avant l'examen, préparez soigneusement votre intestin.
C'est une étape peu agréable mais très importante.

- Suivez le régime recommandé.
- Buvez le liquide recommandé (purge) afin de nettoyer l'intestin (généralement le soir avant l'examen et le jour de l'examen).

Vous recevrez un médicament sédatif. Le gastroentérologue fera l'examen et vous donnera un résultat de vive voix le jour même.

La coloscopie n'a rien montré :

- Important : la coloscopie doit être refaite 10 ans plus tard.
- Si vous avez des symptômes entre-temps, consultez votre médecin sans tarder.

La coloscopie montre des polypes ou un cancer :

- Le médecin enlève généralement les polypes pendant la coloscopie.
- Si le médecin trouve un cancer, il vous expliquera le jour-même la marche à suivre.

Quels sont les avantages et les inconvénients des deux tests ?

	**Test FIT** 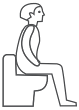	**Coloscopie** 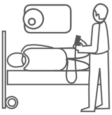
**Quels sont les avantages ?** +	• Le test se fait facilement et rapidement à la maison.<br>• Pas de préparation nécessaire.<br>• Vous évitez de faire une coloscopie si le FIT est négatif.	• C'est la méthode de référence pour trouver les polypes.<br>• Le médecin peut enlever les polypes pendant l'examen.
**Quels sont les inconvénients ?** –	• Si le résultat est positif il faudra quand même faire une coloscopie.<br>• L'attente de la coloscopie peut être stressante.	• Un régime et une préparation de l'intestin sont nécessaires avant l'examen.<br>• Environ 2 personnes sur 1000 (0,2 %) auront un problème sévère (perforation de l'intestin ou hémorragie importante).<br>• Si on vous donne un médicament sédatif (calmant), vous ne pouvez pas conduire pendant 12 à 24 heures.
**À quelle fréquence devez-vous faire le test ?**	• Tous les deux ans	• Tous les dix ans.
**Peut-on faire confiance à ce test ?**	• Le test est fiable s'il est refait tous les deux ans.	• Le test est fiable si vous respectez la préparation chez vous.

Limites du dépistage

Il reste toujours un risque de cancer entre deux tests de dépistage. Il est donc important d'aller chez votre médecin sans tarder si vous avez les symptômes suivants :

- Du sang dans les selles.
- Des troubles digestifs (douleurs, diarrhée ou constipation …).
- Aller à la selle plus souvent ou moins souvent que d'habitude
- Avoir une perte de poids, sans raison connue.
- Ressentir une fatigue qui dure, sans raison connue.

Comment faire le dépistage ?

Si vous avez entre 50 et 69 ans, complétez d'abord ce tableau :

Questions	Oui	Non
Avez-vous l'un ou plusieurs des symptômes suivants : sang dans les selles, troubles digestifs, un transit inhabituel (douleurs, diarrhée ou constipation), selles plus ou moins fréquentes que d'habitude, perte de poids inexpliquée, fatigue durable ?	☐	☐
Avez-vous déjà eu un cancer du côlon ou des polypes ?	☐	☐
Est-ce que quelqu'un dans votre famille directe (parents, frère, sœur) a eu un cancer du côlon avant l'âge de 60 ans ?	☐	☐
Êtes-vous suivi.e pour une maladie inflammatoire du côlon ?	☐	☐

Avez-vous répondu NON à toutes les questions ?

- Si vous souhaitez faire le test FIT, vous pouvez obtenir le test chez un pharmacien.
- Si vous souhaitez faire la coloscopie ou avoir un conseil de votre médecin pour vous décider, prenez rendez-vous avec lui.

Avez-vous répondu OUI à une ou plusieurs questions ?

- Prenez rendez-vous chez votre médecin pour connaître la marche à suivre.

Comment le dépistage est-il pris en charge ?

Les deux tests sont pris en charge par les caisses maladie pour les personnes entre 50 et 69 ans habitant dans le canton de Vaud. Vous ne payerez pas de franchise. Il vous restera à payer la quote-part de 10 %.

Pour plus d'informations

Brochure complète : **www.unisante.ch/colon/brochure.pdf**
Programmes vaudois de dépistage du cancer : **www.pvdc.ch**
Swiss Cancer Screening : **www.swisscancerscreening.ch**
Votre médecin de famille ou votre pharmacien

Dépistage du cancer du côlon
Canton de Vaud

Unisanté
Route de Berne 113, 1010 Lausanne
Tél : 0848 990 990, Fax : 021 314 14 46
depistage.colon@unisante.ch, www.pvdc.ch

unisanté
Centre universitaire de médecine générale et santé publique · Lausanne

Conception/rédaction de cette brochure :
Unisanté – Lausanne en partenariat avec Swiss Cancer Screening, Juin 2021
